# Childhood trajectories of emotional and behavioral difficulties are related to polygenic liability for mood and anxiety disorders

**DOI:** 10.1111/jcpp.14063

**Published:** 2024-10-27

**Authors:** Nora R. Bakken, Nadine Parker, Laurie J. Hannigan, Espen Hagen, Pravesh Parekh, Alexey Shadrin, Piotr Jaholkowski, Evgeniia Frei, Viktoria Birkenæs, Guy Hindley, Laura Hegemann, Elizabeth C. Corfield, Martin Tesli, Alexandra Havdahl, Ole A. Andreassen

**Affiliations:** ^1^ Centre for Precision Psychiatry, Institute of Clinical Medicine University of Oslo and Division of Mental Health and Addiction, Oslo University Hospital Oslo Norway; ^2^ Nic Waals Institute, Lovisenberg Diaconal Hospital Oslo Norway; ^3^ PsychGen Center for Genetic Epidemiology and Mental Health, Norwegian Institute of Public Health Oslo Norway; ^4^ Population Health Sciences, Bristol Medical School University of Bristol Bristol UK; ^5^ Department of Psychology University of Oslo Oslo Norway; ^6^ Centre of Research and Education in Forensic Psychiatry (SIFER) Oslo University Hospital Oslo Norway

**Keywords:** Emotional problems, behavioral problems, polygenic risk (PRS), mood disorder, anxiety disorder, development, MoBa, MBRN

## Abstract

**Background:**

Symptoms related to mood and anxiety disorders (emotional disorders) often present in childhood and adolescence. Some of the genetic liability for mental disorders, and emotional and behavioral difficulties seems to be shared. Yet, it is unclear how genetic liability for emotional disorders and related traits influence trajectories of childhood behavioral and emotional difficulties, and if specific developmental patterns are associated with higher genetic liability for these disorders.

**Methods:**

This study uses data from a genotyped sample of children (*n* = 54,839) from the Norwegian Mother, Father, and Child Cohort Study (MoBa). We use latent growth models (1.5–5 years) and latent profile analyses (1.5–8 years) to quantify childhood trajectories and profiles of emotional and behavioral difficulties and diagnoses. We examine associations between these trajectories and profiles with polygenic scores for bipolar disorder (PGS_BD_), anxiety (PGS_ANX_), depression (PGS_DEP_), and neuroticism (PGS_NEUR_).

**Results:**

Associations between PGS_DEP_, PGS_ANX_, and PGS_NEUR_, and emotional and behavioral difficulties in childhood were more persistent than age‐specific across early childhood (1.5–5 years). Higher PGS_ANX_ and PGS_DEP_ were associated with steeper increases in behavioral difficulties across early childhood. Latent profile analyses identified five profiles with different associations with emotional disorder diagnosis. All PGS were associated with the probability of classification into profiles characterized by some form of difficulties (vs. a normative reference profile), but only PGS_BD_ was uniquely associated with a single developmental profile.

**Conclusions:**

Genetic risk for mood disorders and related traits contribute to both a higher baseline level of, and a more rapid increase in, emotional and behavioral difficulties across early and middle childhood, with some indications for disorder‐specific profiles. Our findings may inform research on developmental pathways to emotional disorders and the improvement of initiatives for early identification and targeted intervention.

## Introduction

Mood and anxiety disorders, often collectively referred to as emotional disorders (Bullis, Boettcher, Sauer‐Zavala, Farchione, & Barlow, 2019), are common and account for a large global burden of disease (Vos et al., 2020). Despite considerable research efforts, the disease mechanisms remain largely unknown (Bennett & Walkup, 2023). The disorders are regarded as multifactorial, influenced by multiple genes in combination with lifestyle and environmental factors (Malhi & Mann, 2018; Penninx, Pine, Holmes, & Reif, 2021; Thapar, Eyre, Patel, & Brent, 2022). Twin and family studies have revealed significant heritability for emotional disorders and related traits, estimated at 60%–90% for bipolar disorders (O'Connell & Coombes, 2021), 30%–50% for major depressive disorder (Kendall et al., 2021), 20%–60% for anxiety disorders (Ask et al., 2021), and 30%–50% for neuroticism (Boomsma et al., 2018; Calboli et al., 2010; Vukasović & Bratko, 2015), which is genetically (Grotzinger et al., 2022; Hindley et al., 2022; Nagel et al., 2018) and phenotypically (Widiger & Oltmanns, 2017) linked to emotional disorders. Recent genome‐wide association studies (GWAS) have shown that common genetic variants account for a substantial part of their heritability (Howard et al., 2019; Mullins et al., 2021; Purves et al., 2020), confirming a polygenic architecture (Andreassen, Hindley, Frei, & Smeland, 2023). The discoveries from GWAS have made it possible to derive polygenic scores (PGS) that capture an individual's genetic liability to these disorders.

Childhood is a critical period in the development of mood and anxiety disorders. Studies in high‐risk populations show that children of parents with an affective disorder are more likely to struggle with mental health issues such as emotional and behavioral problems than those without this family history (Biederman et al., 2004; Lau et al., 2018; Rasic, Hajek, Alda, & Uher, 2013; Weissman et al., 2005). Furthermore, population studies suggest that PGS for emotional disorders and related traits, such as neuroticism, are associated with childhood internalizing symptoms, ADHD symptoms, and social problems (Akingbuwa et al., 2020; Akingbuwa, Hammerschlag, Bartels, & Middeldorp, 2022). However, how genetic liability is linked to developmental trajectories of emotional and behavioral difficulties is unclear (Moyakhe, Dalvie, Mufford, Stein, & Koen, 2023). Better characterization of this relationship is key to understand not only if, but also how and when, genetic liability for mood and anxiety disorders present during childhood development.

Defining robust patterns of mental traits during childhood that may predict emotional disorders is needed to advance treatment options and prevention strategies (Hickie et al., 2013). Despite large advancements during the past decades, treatment options for mental disorders in general, and emotional disorders specifically, are limited (Cuijpers, Stringaris, & Wolpert, 2020). Only 38% of young people receiving treatment for depression and/or anxiety in routine specialist mental health care report a reliable improvement (Bear, Edbrooke‐Childs, Norton, Krause, & Wolpert, 2020), and earlier onset is associated with risk of a more severe course of disorder (Joslyn, Hawes, Hunt, & Mitchell, 2016; Ramsawh, Weisberg, Dyck, Stout, & Keller, 2011; Zisook et al., 2007). This highlights the need for advanced tailored prevention and intervention initiatives targeting youth where current treatment effects are modest and consequences severe. High genetic risk for mood and anxiety disorders have been associated with treatment resistance (Fanelli et al., 2022; Foo et al., 2019; Ward et al., 2018), earlier onset of the disorders, and higher symptom‐burden (Kwong et al., 2021; Wray et al., 2018). Thus, a better understanding of the genetic risk for emotional disorders in relation to trajectories of emotional and behavioral difficulties in childhood can inform the development of more targeted preventive interventions.

In the current study, we aim to identify how polygenic liability for depression (Howard et al., 2019), anxiety (Purves et al., 2020), bipolar disorder (Mullins et al., 2021), and neuroticism (Nagel et al., 2018) associate with developmental trajectories and profiles of childhood emotional and behavioral difficulties (measured at 1.5–8 years of age). We evaluate if these associations are persistent across ages or show age‐specific association patterns. Furthermore, we evaluate if PGS mainly influences the baseline level of emotional and behavioral difficulties or the rate of change in difficulties across childhood. Lastly, we investigate if higher genetic liabilities are associated with the likelihood of being assigned to specific developmental profiles and how these further are related to the risk of clinical diagnosis of emotional disorders. Collectively, we expect that these results will contribute to a deeper understanding of how polygenic liability is involved in the development of mood and anxiety disorders.

## Methods

### Study population

The Norwegian Mother, Father, and Child Cohort Study (MoBa) is a population‐based pregnancy cohort study conducted by the Norwegian Institute of Public Health (NIPH). Participants were recruited from across Norway during the recruitment period of 1999–2008. The women consented to participation in 41% of the pregnancies (Magnus et al., 2006, 2016). Blood samples obtained from the children's umbilical cord at birth were used for genotyping (Paltiel et al., 2014). The current study is based on version 12 of the quality‐assured maternally filled‐out questionnaires released for research in January 2019. Out of the overall sample of 113,530 children, we selected a subset (*n* = 54,839) based on the following criteria: available quality‐controlled (Corfield et al., 2022) genotyping information, coupling to the Medical Birth Registry of Norway (MBRN), and pruning for genetic relatedness using KING version 2.2.5 (Manichaikul et al., 2010). The unrelated function (Manichaikul et al., 2012) was used to extract the maximal list of individuals that contains no first‐ or second‐degree relatives (kinship coefficient threshold < 0.0885). For an overview of the complete sample selection process see Figure S1.

The establishment of MoBa and initial data collection was based on a license from the Norwegian Data Protection Agency and approval from The Regional Committees for Medical and Health Research Ethics (REC). MoBa follows regulations from the Norwegian Health Registry Act. The current study was approved by the administrative board of MoBa led by NIPH and REC (2016/1226/REK Sør‐Øst C).

### Childhood emotional and behavioral difficulties

Measures of emotional and behavioral difficulties were based on questionnaires filled out by the mothers when their children were 1.5, 3, 5, and 8 years old. Emotional and behavioral difficulties in early childhood (1.5, 3, and 5 years) were measured using items from the corresponding subscales of The Child Behavior Checklist (CBCL) that were consistent across the three time points (repeated measures) (Achenbach & Ruffle, 2000). At the age of 8 years (middle childhood), more comprehensive measures of emotional and behavioral difficulties were used: a 13‐item Short Mood and Feelings Questionnaire (SMFQ) measures depressive symptoms (Angold, Costello, Messer, & Pickles, 1995), a 5‐item short form of the Screen for Child Anxiety‐Related Disorders (SCARED) (Birmaher et al., 1999) measures symptoms of anxiety, and a 34‐item Rating Scale for Disruptive Behavior Disorders (RS‐DBD) (Silva et al., 2005) measures symptoms of conduct disorders (8 items), oppositional defiant disorders (8 items), hyperactivity (9 items), and inattention (9 items). The R‐package “Phenotools v0.2.7” (Hannigan et al., 2023) was used to compute scale scores from the individual items. To be included in the computation of a scale score, at least half the subscales' items were required to be nonmissing. Individuals with less than half of the items within a scale were excluded from the analysis. Scale scores were computed on a per‐individual basis by taking the mean of all available individual items for each scale and multiplying by the total number of items in the scale. This was done to reflect the values of the pre‐validated subscales for emotional and behavioral problems. An overview of the individual items included in each measure is presented in Table S1.

### Emotional (mood or anxiety) disorder

Diagnostic information was retrieved from the Norwegian Patient Registry (NPR), where diagnoses from the International Classification of Diseases Tenth Revision (ICD‐10) (World Health Organization, 2004) were registered in specialist health care services from 2008 through 2022. As anxiety and mood disorders commonly co‐occur and have overlapping symptomatology, we examined the disorders as a combined category of emotional disorders, optimizing utility for prevention and early detection (Bullis et al., 2019). The emotional disorders category comprised mood disorders (F30–F39), anxiety disorders (F40–F41), and emotional disorders with onset occurring in childhood and adolescence (F92–F93).

### Covariates

Given known sex differences in the prevalence of emotional disorders and related traits, and the wide range of birth years in MoBa, we included sex and birth year (categorized into three levels, ordered [numeric]) as covariates in all analyses. To account for population stratification effects, the first 10 genetic principal components (PCs) and genotyping batch identifiers were also included as covariates. Parental education level measured at 15 weeks of gestation (categorized into two levels: mother or father with higher education, defined as completed at least regional technical college/4‐year university degree (bachelor's degree), versus no parents with higher education) was included in post hoc sensitivity analyses.

### GWAS summary statistics

This study relied on summary statistics for common emotional disorders and traits that were acquired from the largest GWAS on bipolar disorder (Mullins et al., 2021), anxiety (Purves et al., 2020), depression (Howard et al., 2019), and neuroticism (Nagel et al., 2018). As the bipolar disorder GWAS included MoBa study participants, summary statistics excluding these participants were used. The GWAS for neuroticism was computed on the UK Biobank genotypic samples. A summary table of key descriptives for the GWA studies used in our analyses is presented in the Table S2.

### Statistical analyses

Polygenic scores (PGS): We used the LDpred2 “auto” model (Privé, Arbel, & Vilhjálmsson, 2021) to compute PGS_BD_, PGS_ANX_, PGS_DEP_, and PGS_NEUR_, using their respective GWAS summary statistics and the correlation matrix between genetic variants (Privé, 2022). The four selected PGSs reflect the clinical spectrum of emotional challenges (Bullis et al., 2019), including diagnoses of bipolar disorders, major depressive disorder, anxiety disorders, and strong clinically (Widiger & Oltmanns, 2017) and genetically (Grotzinger et al., 2022; Hindley et al., 2022; Lo et al., 2017; Nagel et al., 2018) correlated trait of neuroticism.

To account for population stratification and batch effects, we performed linear regression with the standardized PGS from LDpred2 as outcome variables and the first 10 genetic PCs and dummy‐encoded genotyping batches (26 batches). We used the standardized residuals from these models for further analyses (henceforth referred to as PGS). For a detailed description of PGS calculation see Appendix S1. The PGS associations to each corresponding trait in our sample are provided in Tables S3 and S4.

### Linear regression models (cross‐sectional associations)

We performed linear regression analyses to study the association between each measure of childhood emotional and behavioral difficulties and the polygenic scores. Separate linear regression models were fit for each of the measures of emotional and behavioral difficulties with the independent variables being PGS_ANX_, PGS_DEP_, PGS_BD_, and PGS_NEUR_ (considered one at a time). To account for multiple testings, we performed a false discovery rate (Benjamini & Hochberg, 1995) correction for the 48 multiple comparisons (12 emotional and behavioral difficulties, four PGS). Prior to running these linear regression models, we standardized each of the 12 measures to have zero mean and unit standard deviations.

### Latent growth models (developmental trajectories in early childhood)

Linear latent growth models were used to assess the influence of polygenic scores on developmental trajectories of emotional and behavioral difficulties across early childhood. First, we defined the latent growth models to model the developmental trajectories of emotional and behavioral difficulties across early childhood. We evaluated the Comparative Fit Index (CFI), Tucker Lewis Index (TLI), and Root Mean Square Error of Approximation (RMSEA) values to determine if a linear growth model was adequate. Next, to assess the association between PGS and these developmental trajectories, we specified five models having different effects of PGS on the developmental trajectories: PGS effect on age‐specific residuals (PGS effects are *age specific*), PGS effect on intercept, slope, or both growth factors (PGS effects are *persistent across age*; three models), and PGS effect fixed to null (PGS effects are *not present*). These models were compared in a “stepwise” manner using change in Akaike Information Criterion (AIC) and *p*‐values from the Chi‐square test at *α* = 0.05, choosing the least complex model when we observed nonsignificant differences (see Appendix S2 for model specification and Appendix S3 for details on this stepwise procedure). At the end of this procedure, we selected eight models (four PGS for two trajectories of emotional and behavioral difficulties). To account for multiple comparisons, we applied a false discovery rate (Benjamini & Hochberg, 1995) correction for the number of PGS effects identified across these eight models. The growth models were constructed using scripts adapted from Hannigan et al. (2021).

### Latent profile analyses (developmental profiles across early and middle childhood)

To extract characteristic developmental patterns of emotional and behavioral difficulties across early and middle childhood (ages 1.5, 3, 5, and 8 years), we used latent profile analyses. We adapted latent profile models from our previous study (Bakken et al., 2023) and additionally included PGS_ANX_, PGS_DEP_, PGS_BD_, and PGS_NEUR_ as covariates. Here a 3‐step maximum likelihood approach (Nylund‐Gibson, Grimm, & Masyn, 2019; Vermunt, 2010) was used to generate and assign profile membership to each individual. Specifically, for each individual, we used their estimated scores on the continuous latent growth factors (from the growth models), scores on emotional and behavioral difficulties at 8 years of age, emotional disorder diagnosis (distal outcome to validate profiles against clinical disorders), and PGS (as covariates; each PGS was used separately) (see Figure S2). We specified models with 2–8 profiles and selected the model (i.e., the number of profiles) by loglikelihood value (LL), first‐order AIC (AIC), second‐order AIC (AIC_c_), Vuong–Lo–Mendell–Rubin likelihood ratio test (VLMR), entropy, the substantive interpretability of each profile, and the proportion of individuals assigned to the smallest profile (we used a threshold of >1%) (Bakken et al., 2023; Petersen, Qualter, & Humphrey, 2019). This model selection procedure was chosen to identify profiles that might be transferable to the general population.

### Sensitivity analyses

We performed a post hoc sensitivity analysis including parental education level as a covariate in all our analyses. This was done to evaluate if our findings were robust to adjustment for socioeconomic status. A post hoc sensitivity analysis constraining covariate effects in the latent growth models to equal effects, instead of letting them vary with age, was also performed.

All analyses were conducted using R version 4.0.3 and Mplus version 8.3 (Muthén & Muthén, 1998–2017). Linear regression models and latent profile analyses were run with the R package “lavaan” version 0.6‐7 (Rosseel, 2012). “MplusAutomation” version 1.0.0 (Hallquist & Wiley, 2018) was used to integrate Mplus and R in the latent profile analyses. Missing data were handled by full information maximum likelihood (FIML). R and Mplus scripts are made openly available: https://github.com/precimed/emo_behav_pgs_traject.

## Results

Demographic and descriptive information for key study variables is presented in Table 1. A flow diagram illustrating the sample population at each stage of analysis is presented in Figure S1. Ordinal Cronbach's alpha for measures of childhood emotional and behavioral difficulties is presented in Table S5.

**Table 1:** Demographic characteristics and descriptive information on key study variables

	Emotional disorder (*n* = 4,968)	No emotional disorder (*n* = 49,871)	Total (*n* = 54,839)
Sex, male, *n* (%)	1827 (36.78%)	26,281 (52.70%)	28,108 (51.26%)
Age end of follow‐up, years, mean (*SD*)	17.60 (2.14)	16.90 (2.15)	16.97 (2.16)
Age first emotional disorder, years, mean, (*SD*)	14.20 (3.54)	–	14.20 (3.54)
Highly educated mother or father, *n* (%)	2,749 (63.55%)	32,636 (73.21%)	35,385 (72.35%)
Maternal age, mean, (*SD*)^a^	29.76 (4.81)	30.25 (4.59)	30.21 (4.61)
Parity, *n* (%) primiparous	22,652 (45.42%)	2,227 (44.83%)	24,879 (45.37%)
Mother's country of birth			
Norway, *n* (%)	4,611 (94.70%)	46,318 (94.48%)	50,929 (94.50%)
Other high‐income‐country, *n* (%)	205 (4.21%)	2,132 (4.35%)	2,337 (4.34%)
Other GDB 7 super region country, *n* (%)	53 (1.09%)	573 (1.17%)	626 (1.16%)
Depressive disorder, *n* (%)	1954 (39.33%)	0 (0.00%)	1954 (3.56%)
Anxiety disorder, *n* (%)	2,853 (57.43%)	0 (0.00%)	2,853 (5.20%)
Bipolar disorder, *n* (%)	118 (2.38%)	0 (0.00%)	118 (0.22%)
ADHD, *n* (%)	1,005 (20.23%)	2,676 (5.37%)	3,681 (6.71%)
Obsessive‐compulsive disorder, *n* (%)	243 (4.89%)	323 (0.65%)	566 (1.03%)
Conduct disorder, *n* (%)	102 (2.05%)	251 (0.50%)	353 (0.64%)
Key study variables, mean, (*SD*)			
Emotional difficulties			
1.5 years	1.45 (1.29)	1.30 (1.21)	1.31 (1.21)
3 years	1.61 (1.52)	1.39 (1.38)	1.41 (1.40)
5 years	1.48 (1.59)	1.05 (1.27)	1.08 (1.30)
Behavioral difficulties			
1.5 years	4.10 (2.32)	3.92 (2.25)	3.94 (2.25)
3 years	4.29 (2.60)	3.83 (2.40)	3.86 (2.42)
5 years	2.97 (2.54)	2.45 (2.23)	2.48 (2.26)
Emotional and behavioral difficulties at age 8 years			
Depressive symptoms	2.77 (3.14)	1.78 (2.36)	1.86 (2.44)
Anxiety symptoms	1.44 (1.57)	0.98 (1.14)	1.02 (1.19)
Inattention	5.80 (4.66)	4.89 (4.06)	4.96 (4.11)
Hyperactivity	4.20 (4.54)	3.52 (3.86)	3.57 (3.92)
Conduct disorder symptoms	0.98 (1.78)	0.73 (1.46)	0.75 (1.49)
Oppositional defiant disorder symptoms	4.33 (3.76)	3.31 (3.06)	3.39 (3.14)

ADHD, attention‐deficit/hyperactivity disorder.

^a^ Mean maternal age at pregnancy is calculated for mothers between ages 17 and 45 due to a lack of information on the age of mothers above or below this age range.

### Cross‐sectional associations between polygenic scores and emotional and behavioral difficulties in early and middle childhood

**Figure 1:**
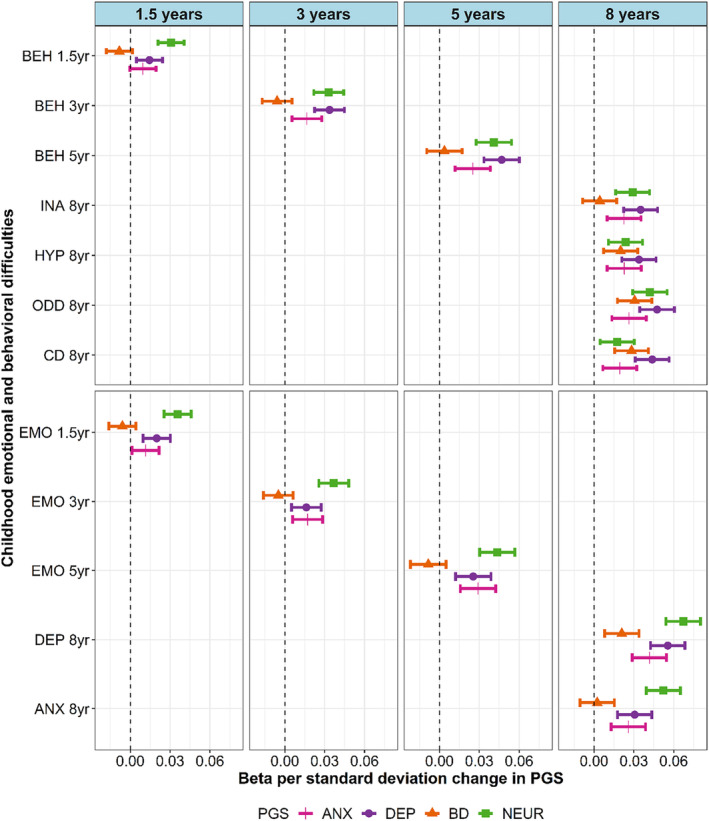
Cross‐sectional associations between polygenic scores (PGS) and emotional and behavioral difficulties across early and middle childhood. *emo1.5 yrs* = emotional difficulties at 1.5 years, *emo3yr* = emotional difficulties at 3 years, *emo5yr* = emotional difficulties at 5 years, *beh1.5 yrs* = behavioral difficulties at 1.5 years, *beh3yr* = behavioral difficulties at 3 years, *beh5yr* = behavioral difficulties at 5 years, *dep8yr* = depressive symptoms at 8 years, *anx8yr* = anxiety symptoms at 8 years, *ina8yr* = inattention symptoms at 8 years, *odd8yr* = oppositional defiant disorder symptoms at 8 years, and *cd8yr* = conduct disorder symptoms at 8 years. ANX, anxiety; BD, bipolar disorder; DEP, depression; NEUR, neuroticism

PGS for depression (PGS_DEP_), neuroticism (PGS_NEUR_), and anxiety (PGS_ANX_) were positively associated with all measures of emotional and behavioral difficulties across early and middle childhood (ages 1.5–8 years) (Figure 1 and Tables S6–S9). PGS for bipolar disorder (PGS_BD_) was only significantly associated with symptoms of depression and oppositional defiant disorder, hyperactivity, and conduct disorder at 8 years (Figure 1 and Table S9).

### Polygenic scores association with developmental trajectories of emotional and behavioral difficulties in early childhood

The results from the latent growth models are presented in Tables 2, S10–S14, and S15–S22. Linear latent growth models provided a good fit for measures of emotional and behavioral difficulties across early childhood (CFI > 0.9, TIL > 0.9, RMSEA < 0.05) (Marsh et al., 2009) (Table S10, Figures S3 and S4). Due to the good fit of the linear model and the advantages of an easily inferable covariate effect we did not explore other model specifications such as a latent basis model. We found PGS_DEP_, PGS_ANX_, and PGS_NEUR_ to have more persistent than age‐specific effects on emotional and behavioral difficulties across early childhood (1.5–5 years). We further found that for PGS_DEP_ the association with emotional difficulties was primarily related to baseline levels of difficulties (*β*_intercept_ = 0.029, 95% CI: 0.018–0.041), while for behavioral difficulties PGS_DEP_ were related to both baseline level and rate of change in difficulties (*β*_intercept_ = 0.021, 95% CI: 0.009–0.034 and *β*_slope_ = 0.041, 95% CI: 0.024–0.058). Higher PGS_ANX_ were related to a more rapid increase in emotional and behavioral difficulties (*β*_slope_ = 0.018, 95% CI: 0.001–0.035 and *β*_slope_ = 0.018, 95% CI: 0.001–0.035), and higher baseline levels of emotional and behavioral difficulties (*β*_intercept_ = 0.015, 95% CI: 0.002–0.029 and *β*_intercept_ = 0.013, 95% CI: 0.001–0.026). PGS_NEUR_ were associated with a higher baseline level of emotional (*β*_intercept_ = 0.055, 95% CI: 0.043–0.066) and behavioral difficulties (*β*_intercept_ = 0.045, 95% CI: 0.035–0.056). We did not find an effect of PGS_BD_ on the trajectories of emotional or behavioral difficulties in our latent growth models (the null effect model was no worse fitting than the simplest model including PGS_BD_ effects). Sensitivity analysis constraining covariate effects to be equal gave the same conclusion for model selection, as in the main analyses where the covariates were freely regressed on the manifest variables (Tables S23–S26).

**Table 2:** Standardized beta for PGS on best performing latent growth model for emotional and behavioral difficulties across early childhood

Model	*β*	95% CI	*p*‐Value	*p*‐FDR
PGS_DEP_ effect on the intercept of emotional difficulties	0.029	(0.018, 0.041)	<.001	<.001
PGS_DEP_ effect on the intercept of behavioral difficulties	0.021	(0.009, 0.034)	.001	.001
PGS_DEP_ effect on the slope of behavioral difficulties	0.041	(0.024, 0.058)	<.001	<.001
PGS_NEUR_ effect on the intercept of emotional difficulties	0.055	(0.043, 0.066)	<.001	<.001
PGS_NEUR_ effect on the intercept of behavioral difficulties	0.045	(0.035, 0.056)	<.001	<.001
PGS_BD_ effect on emotional difficulties	N/A	N/A	N/A	N/A
PGS_BD_ effect on behavioral difficulties	N/A	N/A	N/A	N/A
PGS_ANX_ effect on the intercept of emotional difficulties	0.015	(0.002, 0.029)	.026	.039
PGS_ANX_ effect on the slope of emotional difficulties	0.018	(0.001, 0.035)	.043	.043
PGS_ANX_ effect on the intercept of behavioral difficulties	0.013	(0.001, 0.026)	.034	.042
PGS_ANX_ effect on the slope of behavioral difficulties	0.018	(0.001, 0.035)	.037	.042

p‐FDR = false discovery rate correction for multiple testing using Benjamini–Hochberg false discovery rate procedure. As the model with PGS_BD_ effect fixed to null was not worse performing than growth models including PGS_BD_ effects, no effect estimates are reported for this PGS. 95% CI, 95% confidence interval; ANX, anxiety; BD, bipolar disorder; DEP, depression; NEUR, neuroticism; *p*‐Value, unadjusted *p*‐value; *β*, standardized beta.

### Developmental profiles of emotional and behavioral difficulties across early and middle childhood and their relation to emotional disorders and polygenic scores

**Figure 2:**
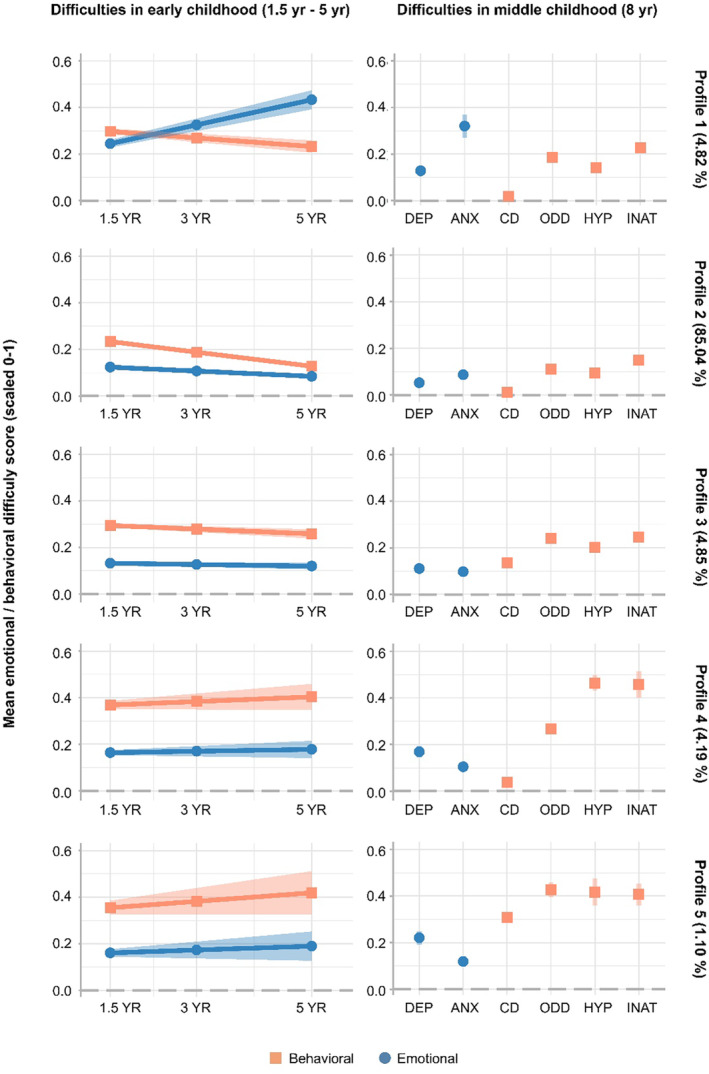
Developmental profiles of emotional and behavioral difficulties across early and middle childhood (1.5–8 years of age). Developmental profiles of childhood emotional and behavioral difficulties. Each row of panels shows developmental profiles of symptoms across early and middle childhood (profiles 1–5). The left panels show the development in early childhood emotional (blue, circle) and behavioral (red, square) difficulties measured by CBCL at 1.5, 3, and 5 years of age. The right panels show further middle childhood mental health traits with specific difficulties specified on the *x*‐axis. 95% confidence intervals are illustrated in shaded colors. ANX, anxiety symptoms; CD, conduct disorder symptoms; DEP, depressive symptoms; HYP, hyperactivity symptoms; INAT, inattention symptoms.; ODD, oppositional defiant disorder symptoms

Based on LL, AIC, AIC_c_, VLMR, entropy, distribution, and the substantive interpretability of each profile we identified 5 developmental profiles of emotional and behavioral difficulties across early and middle childhood (Figure 2, Tables S27–S33). The identified profiles were consistent with the profiles derived from the complete MoBa sample (Bakken et al., 2023).

*Profile 1 (4.82% of the sample)*: characterized by elevated and increasing emotional difficulties and slightly elevated behavioral difficulties across early childhood, and elevated levels of anxiety at the age of 8 years (“Anxiety profile”). *Profile 2 (85.04%):* characterized by low and decreasing levels of emotional and behavioral difficulties in early childhood (1.5–5 years), and low levels of emotional and behavioral difficulties at the age of 8 years (“Reference profile”). *Profile 3 (4.85%):* characterized by slower decreasing behavioral symptoms across early childhood, and slightly elevated behavioral difficulties at the age of 8 years (“Delayed decreasing profile”). *Profile 4 (4.19%):* characterized by elevated levels of emotional and behavioral difficulties across early childhood, and elevated levels of hyperactivity, inattention, and oppositional defiant disorder symptoms at 8 years (“ADHD profile”). *Profile 5 (1.10%)*: characterized by increasing and elevated behavioral and emotional difficulties across early childhood and elevated levels of depression, hyperactivity, inattention, oppositional defiant, and conduct disorder symptoms at 8 years (“Behavioral dysregulation profile”).

Profiles 5 and 4, and profiles 2 and 3 are similar in appearance. However, as statistical fit indices indicated a 5 profile model to be the best fitting and we believe these profiles encompass important phenotypical differences of potential clinical importance we decided to go further with a 5 profile model.

Profiles 5 and 1, dominated by increasing and/or high levels of behavioral and emotional difficulties in childhood, had the strongest relationship with later diagnoses of emotional disorders (Table S29).

**Figure 3:**
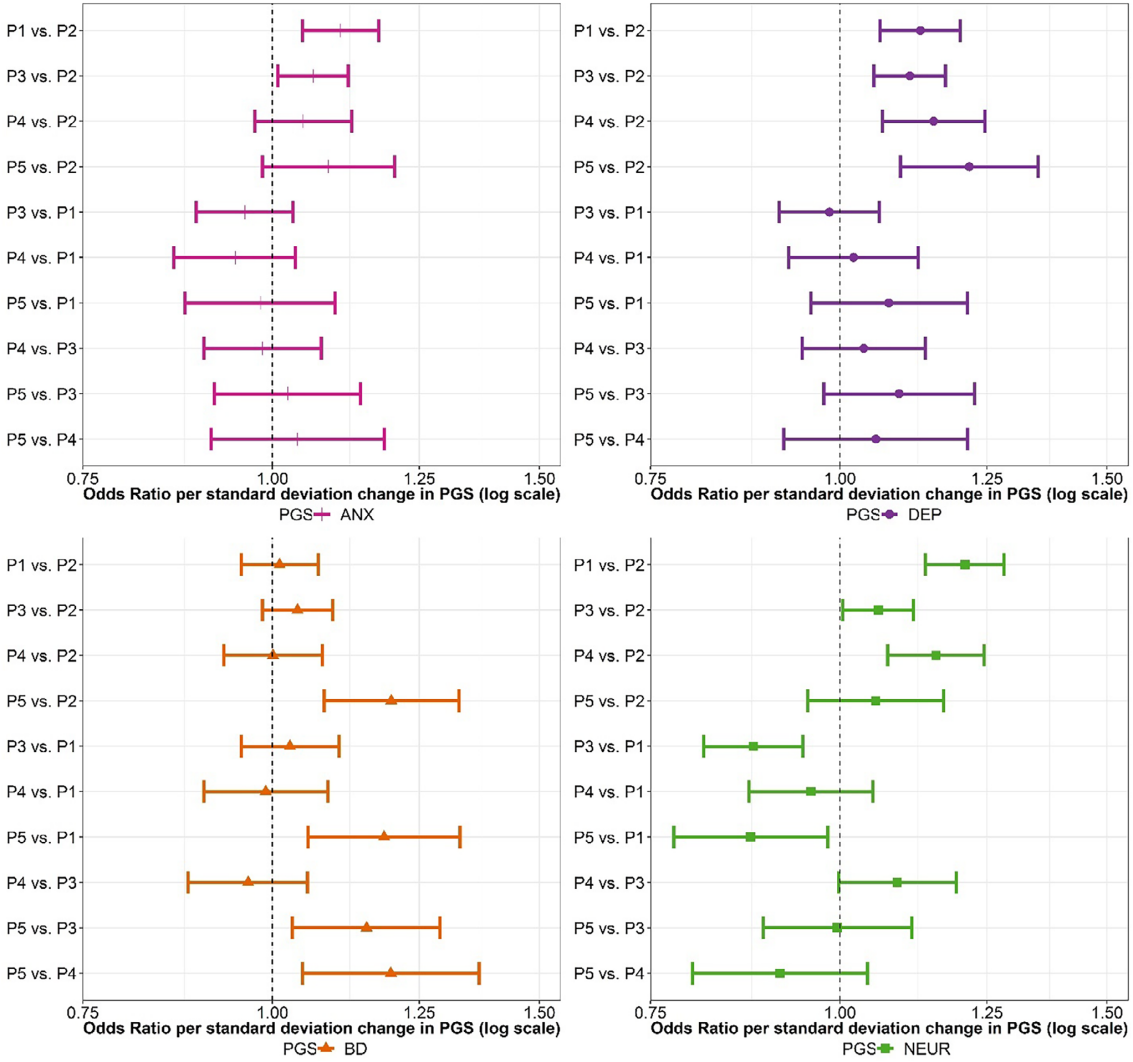
Relationship between polygenic scores and developmental profiles of emotional and behavioral difficulties across early and middle childhood (1.5–8 years of age). Odds ratio comparing odds of being assigned to individual profiles per standard deviation increase in polygenic score (PGS) showed with 95% confidence intervals. ANX, anxiety; BD, bipolar disorder; DEP, depression; NEUR, neuroticism; P1, Profile 1; P2, Profile 2; P3, Profile 3; P4, Profile 4; P5, Profile 5

All PGS were associated with the likelihood of being assigned to a symptomatic profile rather than the reference profile (profile 2) in which symptoms were low and decreasing throughout childhood (Figure 3, Tables S30–S34). Individuals with higher PGS_DEP_ were more likely to be assigned to profile 1 (“Anxiety”), 3 (“Delayed decreasing”), 4 (“ADHD”), or 5 (“Behavioral dysregulation”), compared to the reference profile (Figure 3, Table S30). Individuals with higher PGS_ANX_ were more likely to be assigned to profile 1 (“Anxiety”) or 3 (“Delayed decreasing”) compared to the reference profile (Figure 3, Table S31). Individuals with higher PGS_BD_ were most likely to be assigned to profile 5 (“Behavioral dysregulation”) (Figure 3, Table S31). Individuals with higher PGS_NEUR_ were more likely to be assigned to profile 1 (“Anxiety”), 3 (“Delayed decreasing”), or 4 (“ADHD”) compared to the reference profile. PGS_NEUR_ were more strongly associated with profile 1 (“Anxiety”), than profile 3 (“Delayed decreasing”) and profile 5 (“Behavioral dysregulation”), but no differences were found when compared to profile 4 (“ADHD”) (Figure 3, Table S32). No differences between the odds of assignment to the different high‐symptom profiles (Profiles 1, 3, 4, and 5) were found for PGS_DEP_ and PGS_ANX_ (Figure 3, Tables S30 and S31).

### Sensitivity analysis including parental education as a covariate

Sensitivity analyses including parental education as a covariate are presented in Tables S34–S56. Positive associations between PGS_DEP_, PGS_ANX_, PGS_NEUR_, and PGS_BD_ as identified in the main cross‐sectional analyses were replicated except for significance in the relation between PGS_DEP_ and behavioral difficulties at 1.5 years of age, and PGS_ANX_ and emotional and behavioral difficulties at the age of 1.5 years. Results from the latent growth models were replicated, except for the relation between PGS_ANX_ and baseline level (intercept) of emotional and behavioral difficulties. Relations between PGS_DEP_, PGS_ANX_, PGS_BD_, and PGS_NEUR_ and latent profiles were also robust to the inclusion of parental education as a covariate, except for an identified difference in PGS_NEUR_ relation to profile 3 versus the reference profile (profile 2).

## Discussion

The main findings of the current study were that genetic liability for emotional disorders is associated with both higher baseline and more rapidly increasing levels of emotional and behavioral difficulties across early and middle childhood, and that the genetic effect on developmental trajectories was mainly persistent across development (influence on latent growth factors) and less age specific. Furthermore, we find that PGS_DEP_ and PGS_ANX_ show a general association with developmental profiles with higher levels of difficulties, while PGS_BD_ is specifically linked to a developmental profile characterized by elevated and increasing emotional and behavioral difficulties across early childhood, followed by elevated levels of depression and behavioral difficulties at 8 years of age (“Behavioral dysregulation,” profile 5). Overall, we find that children with higher genetic liability for emotional disorders are more likely to present with higher and/or more rapidly increasing levels of difficulties across childhood, difficulties that here and previously (Bakken et al., 2023) have been linked to the likelihood of receiving a clinical diagnosis.

Our findings indicate that PGS_DEP_, PGS_ANX_, and PGS_NEUR_ show a persistent association with emotional and behavioral difficulties across development. This is consistent with a cross‐cohort meta‐analysis showing no evidence of age as a moderator for the association between PGS_DEP_ or PGS_NEUR_ in relation to childhood ADHD problems or internalizing problems (Akingbuwa et al., 2020) and the association between PGS_SCZ_ and similar childhood measures as in the present study (Hannigan et al., 2021). The identified stability across age is a novel finding, which can be interpreted in several ways. The persistence in genetic association with emotional and behavioral difficulties can suggest that the genetic variants in combination influence underlying pathophysiological processes, such as neurobiological influences across the early childhood years (1.5–5 years). In addition, it is possible that the relevant variants contribute to pathophysiological processes in utero and/or infancy, which could lead to difficulties later in life. From a clinical point of view, the persistence of genetic associations to emotional and behavioral difficulties implies that genetic risk is a consistent risk factor for symptoms across early childhood, with no indications of a narrow, time‐limited, sensitive period to tailor preventive or early detection initiatives after. From a conceptual etiological point of view, our study is coherent with studies indicating genetic contribution to continuity in emotional symptoms across childhood and the genetic relation between childhood internalizing symptoms and adult emotional disorders (Jami et al., 2022).

Nonetheless, as indicated from the cross‐sectional analyses, the results from the latent growth models only show that the genetic effects are primarily persistent across early childhood, not that there are no age‐specific effects at all. Age‐specific genetic effects are indicated in twin studies of internalizing and externalizing behaviors (Van Beijsterveldt, Bartels, Hudziak, & Boomsma, 2003) across early to late childhood (Bartels et al., 2004). Age‐specific genetic associations are also coherent with findings of SNP heritability estimates varying with age (Akingbuwa et al., 2022). However, longitudinal analyses on age‐specific heritability are lacking and one should be cautious in bridging inferences from cross‐sectional versus longitudinal studies as these do not capture the same effects (Kievit, Logan, & Hart, 2022). Methodological differences might induce differences in heritability estimates. Furthermore, differences in within‐person processes, such as the identified relation between PGS_ANX_ and PGS_DEP_ and the slope of behavioral difficulties, may also influence the cross‐sectional estimates, which together with changes in environmental influence will affect the age‐restrained genetic relation to the trait.

In our main analyses, we find that polygenic liabilities for anxiety and depression are associated with both a steeper increase, and a higher baseline level of behavioral difficulties across early childhood. A genetically mediated acceleration in difficulties can be explained in several ways. First, these findings are coherent with theories of gene–environment correlations. Gene–environment correlations are the process in which an individual's genotype influences, or is linked to, their exposure to environmental factors (Knafo & Jaffee, 2013). In this study, we show that PGS_ANX_ and PGS_DEP_ are linked to higher levels of emotional and behavioral difficulties across childhood. The genetically linked elevated levels of difficulties could contribute to increased vulnerability or exposure to environmental stressors such as more negative attention from parents and/or other adult figures (Mcclowry et al., 2013), sensitivity to bullying, and problems with peer relationships (Klima & Repetti, 2008; Perren et al., 2006). Thus, further contributing to accelerated experience of emotional and behavioral difficulties, as identified in this study. Furthermore, neurobiological differences are associated with genetic risk for mood disorders and related traits (Alex et al., 2023; Alnæs et al., 2019; Cheng et al., 2022; Fernandez‐Cabello et al., 2022; Patel et al., 2021; Shen et al., 2020), indicating potential for a neurodevelopmental component directly or indirectly contributing to higher and increasing emotional and behavioral difficulties, as well as risk of disorder.

Including parental education as a covariate in our sensitivity analyses, removed the significance of the cross‐sectional association between PGS_ANX_ and difficulties at the age of 1.5 years, as well as the relation between PGS_ANX_ and baseline level (intercept) identified in the latent growth models. The reduced relation to baseline level when including parental education as a covariate could be because parental education acts as a confounding factor attenuating a weak association at model inclusion. However, parental education has also been identified as a moderator of genetic risk and emotional and behavioral difficulties (Badini et al., 2024), has been linked to changes in methylation profiles (Uddin et al., 2013) and was shown to overlap with mental disorders on the genetic level (Marees et al., 2021), thus more complex relationships could be involved and should be properly investigated.

From a clinical and public health perspective, our findings indicate that genetic information may help identify the children who are more likely to follow a less favorable trajectory of higher symptom burden across childhood. First, the relationship between PGS and more rapidly increasing difficulties across early childhood is an important finding, illustrating that there are individuals with an inherent susceptibility to a more rapid increase in difficulties, and therefore may be in need of adjusted preventive efforts and/or intensified care. Furthermore, PGS_DEP_, PGS_ANX_, and PGS_NEUR_ were all related to developmental profiles with higher levels of emotional and/or behavioral difficulties across early to middle childhood (collectively encompassing 15% of the study population), highlighting that genetic information might help identify individuals with an increased likelihood of consistently higher levels of difficulties than the reference population. PGS_BD_ was specifically associated with profile 5 characterized by elevated and increasing behavioral symptoms, high levels of depression, conduct disorder symptoms, oppositional defiant disorder symptoms, and hyperactivity and inattention at 8 years of age (“Behavioral dysregulation profile”). The link between this developmental pattern and genetic risk for bipolar disorder is consistent with studies finding CBCL‐pediatric bipolar disorder phenotype (CBCL‐PBD) and measures of emotional dysregulation in childhood to be related to the later development of BD in adolescence and adulthood (Biederman et al., 2009; Disalvo et al., 2023; Meyer et al., 2009). Furthermore, in our analyses, these results seem to be driven by a pattern of difficulties at 8 years of age. The lack of association between emotional and behavioral difficulties, and PGS_BD_ in early childhood is consistent with findings from smaller and/or cross‐sectional studies (Akingbuwa et al., 2020; Biederman, Green, Disalvo, & Faraone, 2021; Duffy & Carlson, 2013). Our findings of a specific and general link between genetic liability and unfavorable developmental profiles of emotional and behavioral difficulties indicate that information on genetic risk may be an informative additional objective marker in risk evaluation of children presenting with mood‐related symptoms. However, it is important to emphasize that currently, genetic risk as captured by PGS, only explains a small amount of variation in symptom burden. Therefore, further studies are needed to evaluate the utility of PGS in clinical samples and for individual‐level predictions.

Strengths of the current study include a large number of individuals from a prospective population‐based cohort, providing the opportunity to detect small effects, typical in complex phenotypes with polygenic architectures. Access to repeated measures of symptoms provides the opportunity to investigate both cross‐sectional measures and trajectories. The longitudinal data enabled us to determine relationships between genetic risk and symptoms across time. Nevertheless, there are also several limitations to consider. First, genetic liability as measured by PGS only captures relative risk based on additive effects of a subset of identified SNPs. Thus, nonadditive effects and rare variants are not investigated, which may also influence the tendencies identified in this study (Kendall et al., 2019; Rees & Kirov, 2021). Second, PGSs are only available for individuals of European ancestry, thus reducing the generalizability to other ancestries. Further, the present measures of emotional and behavioral difficulties are based on questionnaires completed by mothers, which could bias the assessments. As the measures used at age 8 were different from those used at ages 1.5, 3, and 5 years, we could not evaluate growth trajectories and age‐specificity across the entire study period. Therefore, it is unknown if associations identified for bipolar disorder at age 8 alone may be a product of differing assessment tools. Last, the summary statistics from the GWAS are based on genetic risk for adult diagnoses. Some studies indicate that other variants may contribute to an earlier onset of the disorder (Harder et al., 2022; Kang et al., 2021; Power et al., 2012, 2017), but this is still not well established. In the future, as GWAS on children and adolescents will become available, our findings should be replicated with PGS derived from child and adolescent diagnosis alone and combined with diagnosis at an older age.

## Conclusion

In conclusion, we find that polygenic liability for anxiety, depression, and neuroticism present in emotional and behavioral difficulties in early childhood with a persistent, rather than age‐specific effect. The genetic liability for anxiety and depression showed a general association with developmental profiles with higher levels of difficulties, while the genetic risk for bipolar disorder was specifically related to symptoms consistent with behavioral dysregulation. Our results show that genetic risk for emotional disorders and related traits contribute to a collectively higher burden, and a faster increase in, emotional and behavioral difficulties across early and middle childhood. The findings suggest a potential presence of behavioral and emotional premorbid characteristics associated with genetic and clinical risk of disorders, and a potential role of PGS in improving efforts for early identification and targeted preventive interventions.

## Ethical considerations

The establishment of MoBa and initial data collection was based on a license from the Norwegian Data Protection Agency and approval from The Regional Committees for Medical and Health Research Ethics (REC). MoBa follows regulations from the Norwegian Health Registry Act. The current study was approved by the administrative board of MoBa led by NIPH and REC (2016/1226/REK Sør‐Øst C).

**Key points**

- There is a relationship between emotional and behavioral difficulties in childhood and genetic risk of mood and anxiety disorders (emotional disorders).
- The association between genetic liability for anxiety, depression, and neuroticism, and emotional and behavioral difficulties in childhood is persistent across early and middle childhood. Genetic risk for depression and anxiety is associated with higher baseline levels, and a more rapid increase, in emotional and behavioral difficulties during childhood.
- The trajectories of emotional and behavioral difficulties associated with genetic susceptibility for mental disorders indicate potential opportunities for improvement of early identification and targeted intervention efforts.

## Supporting information


**Figure S1.** Flow diagram for the study sample.
**Table S1.** Questions included in each childhood emotional and behavioral difficulty measure.
**Table S2.** Key descriptives for original GWAS.
**Appendix S1.** Detailed description of PGS calculation and evaluation procedure.
**Table S3.** PGS relation to depression, anxiety, and bipolar disorder in our sample.
**Table S4.** PGS relation to neuroticism in our sample.
**Appendix S2.** Lavaan model syntax for the latent growth models.
**Appendix S3.** Detailed description of the stepwise growth model selection procedure.
**Figure S2.** Latent profile analysis incorporating latent growth models for developmental profiles of emotional and behavioral difficulties and polygenic scores.
**Table S5.** Ordinal Cronbach's alpha for each childhood emotional and behavioral difficulty measure.
**Table S6.** Depression PGS. Linear association between standardized PGS score and standardized score of emotional and behavioral difficulties.
**Table S7.** Neuroticism PGS. Linear association between standardized PGS score and standardized score of emotional and behavioral difficulties.
**Table S8.** Anxiety disorder PGS. Linear association between standardized PGS score and standardized score of emotional and behavioral difficulties
**Table S9.** Bipolar disorder PGS. Linear association between standardized PGS score and standardized score of emotional and behavioral difficulties.
**Table S10.** Model fit for basic linear latent growth models across early childhood.
**Figure S3.** Trajectories of emotional difficulties across early childhood (1.5, 3, and 5 years).
**Figure S4.** Trajectories of behavioral difficulties across early childhood (1.5, 3, and 5 years).
**Table S11.** Evaluating the best fitting latent growth model for emotional and behavioral difficulties for PGS of depression.
**Table S12.** Evaluating the best fitting latent growth model for emotional and behavioral difficulties for PGS of anxiety.
**Table S13.** Evaluating the best fitting latent growth model for emotional and behavioral difficulties and PGS of neuroticism.
**Table S14.** Evaluating the best fitting latent growth model for emotional and behavioral difficulties for PGS of bipolar disorder.
**Table S15.** Full model output for best fitting latent growth model including trajectories of emotional difficulties and PGS of depression.
**Table S16.** Full model output for best fitting latent growth model including trajectories of behavioral difficulties and PGS of depression.
**Table S17.** Full model output for best fitting latent growth model including trajectories of emotional difficulties and PGS of anxiety.
**Table S18.** Full model output for best fitting latent growth model including trajectories of behavioral difficulties and PGS of anxiety.
**Table S19.** Full model output for best fitting latent growth model including trajectories of behavioral difficulties and PGS of neuroticism.
**Table S20.** Full model output for best fitting latent growth model including trajectories of emotional difficulties and PGS of neuroticism.
**Table S21.** Full model output for best fitting latent growth model including trajectories of emotional difficulties and PGS of bipolar disorder.
**Table S22.** Full model output for best fitting latent growth model including trajectories of behavioral difficulties and PGS of bipolar disorder.
**Table S23.** Evaluating best fitting latent growth model for emotional and behavioral difficulties for PGS of depression with covariate effects constrained to equal.
**Table S24.** Evaluating best fitting latent growth model for emotional and behavioral difficulties for PGS of anxiety with covariate effects constrained to equal.
**Table S25.** Evaluating best fitting latent growth model for emotional and behavioral difficulties and PGS of neuroticism with covariate effects constrained to equal.
**Table S26.** Evaluating best fitting latent growth model for emotional and behavioral difficulties for PGS of bipolar disorder with covariate effects constrained to equal.
**Table S27.** Model fit statistics for latent profile analyses.
**Table S28.** Distribution of individuals in each profile with a five‐profile model.
**Table S29.** Relative odds of any emotional disorder given the assignment to a specific developmental profile.
**Table S30.** Relative odds of assignment to specific developmental profile per standard deviation increase in polygenic score for depression.
**Table S31.** Relative odds of assignment to specific developmental profile per standard deviation increase in polygenic score for anxiety.
**Table S32.** Relative odds of assignment to specific developmental profile per standard deviation increase in polygenic score for bipolar disorder.
**Table S33.** Relative odds of assignment to specific developmental profile per standard deviation increase in polygenic score for neuroticism.
**Table S34.** Depression PGS. Linear association between standardized PGS score and standardized score of emotional and behavioral difficulties including parental education as covariate.
**Table S35.** Neuroticism PGS. Linear association between standardized PGS score and standardized score of emotional and behavioral difficulties including parental education as covariate.
**Table S36.** Anxiety disorder PGS. Linear association between standardized PGS score and standardized score of emotional and behavioral difficulties including parental education as covariate.
**Table S37.** Bipolar disorder PGS. Linear association between standardized PGS score and standardized score of emotional and behavioral difficulties including parental education as covariate.
**Table S38.** Model fit for basic linear latent growth models across early childhood including parental education as a covariate.
**Table S39.** Evaluating best fitting latent growth model for emotional and behavioral difficulties for PGS of depression including parental education as a covariate.
**Table S40.** Evaluating best fitting latent growth model for emotional and behavioral difficulties for PGS of anxiety including parental education as a covariate.
**Table S41.** Evaluating best fitting latent growth model for emotional and behavioral difficulties and PGS of neuroticism including parental education as a covariate.
**Table S42.** Evaluating best fitting latent growth model for emotional and behavioral difficulties for PGS of bipolar disorder including parental education as a covariate.
**Table S43.** Standardized beta for PGS on best performing latent growth model for emotional and behavioral difficulties across early childhood including parental education as a covariate.
**Table S44.** Full model output for best fitting latent growth model including trajectories of emotional difficulties and PGS of depression including parental education as a covariate.
**Table S45.** Full model output for best fitting latent growth model including trajectories of behavioral difficulties and PGS of depression including parental education as a covariate.
**Table S46.** Full model output for best fitting latent growth model including trajectories of emotional difficulties and PGS of anxiety including parental education as a covariate.
**Table S47.** Full model output for best fitting latent growth model including trajectories of behavioral difficulties and PGS of anxiety including parental education as a covariate.
**Table S48.** Full model output for best fitting latent growth model including trajectories of emotional difficulties and PGS of neuroticism including parental education as a covariate.
**Table S49.** Full model output for best fitting latent growth model including trajectories of behavioral difficulties and PGS of neuroticism including parental education as a covariate.
**Table S50.** Full model output for best fitting latent growth model including trajectories of emotional difficulties and PGS of bipolar disorder including parental education as a covariate.
**Table S51.** Full model output for best fitting latent growth model including trajectories of behavioral difficulties and PGS of bipolar disorder including parental education as a covariate.
**Table S52.** Relative odds of any emotional disorder given the assignment to a specific developmental profile including parental education as a covariate.
**Table S53.** Relative odds of assignment to specific developmental profile per standard deviation increase in polygenic score for depression including parental education as a covariate.
**Table S54.** Relative odds of assignment to specific developmental profile per standard deviation increase in polygenic score for anxiety including parental education as a covariate.
**Table S55.** Relative odds of assignment to specific developmental profile per standard deviation increase in polygenic score for bipolar disorder including parental education as a covariate.
**Table S56.** Relative odds of assignment to specific developmental profile per standard deviation increase in polygenic score for neuroticism including parental education as a covariate.

## Data Availability

Data from MoBa and MBRN used in this study are managed by the national health register holders in Norway (NIPH) and can be made available to researchers, provided approval from REC, compliance with the EU General Data Protection Regulation (GDPR), and approval from the data owners. The consent given by the participants does not open for storage of data on an individual level in repositories or journals. Researchers who want access to data sets for replication should apply through helsedata.no. Access to data sets requires approval from REC in Norway and an agreement with MoBa.
