# Supplementary material for: Childhood trajectories of emotional and behavioral difficulties are related to polygenic liability for mood and anxiety disorders
Source: J Child Psychol Psychiatry. 2024 Oct 27;66(3):350–65. doi: 10.1111/jcpp.14063 (PMC11812494; doi:10.1111/jcpp.14063)
Supplement: Supplementary file 1 — Figure S1. Flow diagram for the study sample. Table S1. Questions included in each childhood emotional and behavioral difficulty measure. Table S2. Key descriptives for original GWAS. Appendix S1. Detailed description of PGS calculation and evaluation procedure. Table S3. PGS relation to depression, anxiety, and bipolar disorder in our sample. Table S4. PGS relation to neuroticism in our sample. Appendix S2. Lavaan model syntax for the latent growth models. Appendix S3. Detailed description of the stepwise growth model selection procedure. Figure S2. Latent profile analysis incorporating latent growth models for developmental profiles of emotional and behavioral difficulties and polygenic scores. Table S5. Ordinal Cronbach's alpha for each childhood emotional and behavioral difficulty measure. Table S6. Depression PGS. Linear association between standardized PGS score and standardized score of emotional and behavioral difficulties. Table S7. Neuroticism PGS. Linear association between standardized PGS score and standardized score of emotional and behavioral difficulties. Table S8. Anxiety disorder PGS. Linear association between standardized PGS score and standardized score of emotional and behavioral difficulties Table S9. Bipolar disorder PGS. Linear association between standardized PGS score and standardized score of emotional and behavioral difficulties. Table S10. Model fit for basic linear latent growth models across early childhood. Figure S3. Trajectories of emotional difficulties across early childhood (1.5, 3, and 5 years). Figure S4. Trajectories of behavioral difficulties across early childhood (1.5, 3, and 5 years). Table S11. Evaluating the best fitting latent growth model for emotional and behavioral difficulties for PGS of depression. Table S12. Evaluating the best fitting latent growth model for emotional and behavioral difficulties for PGS of anxiety. Table S13. Evaluating the best fitting latent growth model for emotional and behavioral difficulti [file JCPP-66-350-s001.docx]

**Supporting information:**

Bakken et al. Childhood trajectories of emotional and behavioral difficulties are related to polygenic liability for mood and anxiety disorders.

Contents

[Figure S1 Flow diagram for study sample 5](#_Toc180693781)

[Table S1 Questions included in each childhood emotional and behavioral difficulty measure. 6](#_Toc180693782)

[Table S2. Key descriptives for original GWAS: 10](#_Toc180693783)

[Appendix S1. Detailed description of PGS calculation and evaluation procedure 12](#_Toc180693784)

[Table S3 PGS relation to depression, anxiety and bipolar disorder in our sample 14](#_Toc180693785)

[Table S4 PGS relation to neuroticism in our sample 14](#_Toc180693786)

[Appendix S2. Lavaan model syntax for the latent growth models 15](#_Toc180693787)

[Appendix S3. Detailed description of the stepwise growth model selection procedure 23](#_Toc180693788)

[Figure S2: Latent profile analysis incorporating latent growth models for developmental profiles of emotional and behavioral difficulties and polygenic scores. 25](#_Toc180693789)

[Table S5 Ordinal Cronbach’s alpha for each childhood emotional and behavioral difficulty measure 27](#_Toc180693790)

[Table S6: Depression PGS. Linear association between standardized PGS score and standardized score of emotional and behavioral difficulties 28](#_Toc180693791)

[Table S7: Neuroticism PGS. Linear association between standardized PGS score and standardized score of emotional and behavioral difficulties. 29](#_Toc180693792)

[Table S8: Anxiety disorder PGS. Linear association between standardized PGS score and standardized score of emotional and behavioral difficulties. 30](#_Toc180693793)

[Table S9: Bipolar disorder PGS. Linear association between standardized PGS score and standardized score of emotional and behavioral difficulties 31](#_Toc180693794)

[Table S10 Model fit for basic linear latent growth models across early childhood 32](#_Toc180693795)

[Figure S3 Trajectories of emotional difficulties across early childhood (1.5, 3 and 5 years) 33](#_Toc180693796)

[Figure S4 Trajectories of behavioral difficulties across early childhood (1.5, 3 and 5 years) 33](#_Toc180693797)

[Table S11 Evaluating best fitting latent growth model for emotional and behavioral difficulties for PGS of depression 34](#_Toc180693798)

[Table S12 Evaluating best fitting latent growth model for emotional and behavioral difficulties for PGS of anxiety 35](#_Toc180693799)

[Table S13 Evaluating best fitting latent growth model for emotional and behavioral difficulties and PGS of neuroticism 36](#_Toc180693800)

[Table S14 Evaluating best fitting latent growth model for emotional and behavioral difficulties for PGS of bipolar disorder 37](#_Toc180693801)

[Table S15 Full model output for best fitting latent growth model including trajectories of emotional difficulties and PGS of depression 39](#_Toc180693802)

[Table S16 Full model output for best fitting latent growth model including trajectories of behavioral difficulties and PGS of depression 40](#_Toc180693803)

[Table S17 Full model output for best fitting latent growth model including trajectories of emotional difficulties and PGS of anxiety 41](#_Toc180693804)

[Table S18 Full model output for best fitting latent growth model including trajectories of behavioral difficulties and PGS of anxiety 42](#_Toc180693805)

[Table S19 Full model output for best fitting latent growth model including trajectories of behavioral difficulties and PGS of neuroticism 43](#_Toc180693806)

[Table S20 Full model output for best fitting latent growth model including trajectories of emotional difficulties and PGS of neuroticism 44](#_Toc180693807)

[Table S21 Full model output for best fitting latent growth model including trajectories of emotional difficulties and PGS of bipolar disorder 45](#_Toc180693808)

[Table S22 Full model output for best fitting latent growth model including trajectories of behavioral difficulties and PGS of bipolar disorder 46](#_Toc180693809)

[Table S23 Evaluating best fitting latent growth model for emotional and behavioral difficulties for PGS of depression with covariate effects constrained to equal 47](#_Toc180693810)

[Table S24 Evaluating best fitting latent growth model for emotional and behavioral difficulties for PGS of anxiety with covariate effects constrained to equal 48](#_Toc180693811)

[Table S25 Evaluating best fitting latent growth model for emotional and behavioral difficulties and PGS of neuroticism with covariate effects constrained to equal 49](#_Toc180693812)

[Table S26 Evaluating best fitting latent growth model for emotional and behavioral difficulties for PGS of bipolar disorder with covariate effects constrained to equal 50](#_Toc180693813)

[Table S27: Model fit statistics for latent profile analyses 52](#_Toc180693814)

[Table S28: Distribution of individuals in each profile with five-profile-model 52](#_Toc180693815)

[Table S29: Relative odds of any emotional disorder given assignment to specific developmental profile 53](#_Toc180693816)

[Table S30: Relative odds of assignment to specific developmental profile per standard deviation increase in polygenic score for depression. 54](#_Toc180693817)

[Table S31: Relative odds of assignment to specific developmental profile per standard deviation increase in polygenic score for anxiety. 54](#_Toc180693818)

[Table S32: Relative odds of assignment to specific developmental profile per standard deviation increase in polygenic score for bipolar disorder. 55](#_Toc180693819)

[Table S33: Relative odds of assignment to specific developmental profile per standard deviation increase in polygenic score for neuroticism. 55](#_Toc180693820)

[Table S34: Depression PGS. Linear association between standardized PGS score and standardized score of emotional and behavioral difficulties including parental education as covariate. 56](#_Toc180693821)

[Table S35: Neuroticism PGS. Linear association between standardized PGS score and standardized score of emotional and behavioral difficulties including parental education as covariate. 57](#_Toc180693822)

[Table S36: Anxiety disorder PGS. Linear association between standardized PGS score and standardized score of emotional and behavioral difficulties including parental education as covariate. 58](#_Toc180693823)

[Table S37: Bipolar disorder PGS. Linear association between standardized PGS score and standardized score of emotional and behavioral difficulties including parental education as covariate 59](#_Toc180693824)

[Table S38 Model fit for basic linear latent growth models across early childhood including parental education as covariate 60](#_Toc180693825)

[Table S39 Evaluating best fitting latent growth model for emotional and behavioral difficulties for PGS of depression including parental education as covariate 61](#_Toc180693826)

[Table S40 Evaluating best fitting latent growth model for emotional and behavioral difficulties for PGS of anxiety including parental education as covariate 62](#_Toc180693827)

[Table S41 Evaluating best fitting latent growth model for emotional and behavioral difficulties and PGS of neuroticism including parental education as covariate 63](#_Toc180693828)

[Table S42 Evaluating best fitting latent growth model for emotional and behavioral difficulties for PGS of bipolar disorder including parental education as covariate 64](#_Toc180693829)

[Table S43 Standardized beta for PGS on best performing latent growth model for emotional and behavioral difficulties across early childhood including parental education as covariate 66](#_Toc180693830)

[Table S44 Full model output for best fitting latent growth model including trajectories of emotional difficulties and PGS of depression including parental education as a covariate 67](#_Toc180693831)

[Table S45 Full model output for best fitting latent growth model including trajectories of behavioral difficulties and PGS of depression including parental education as a covariate 68](#_Toc180693832)

[Table S46 Full model output for best fitting latent growth model including trajectories of emotional difficulties and PGS of anxiety including parental education as a covariate 69](#_Toc180693833)

[Table S47 Full model output for best fitting latent growth model including trajectories of behavioral difficulties and PGS of anxiety including parental education as a covariate 70](#_Toc180693834)

[Table S48 Full model output for best fitting latent growth model including trajectories of emotional difficulties and PGS of neuroticism including parental education as a covariate 71](#_Toc180693835)

[Table S49 Full model output for best fitting latent growth model including trajectories of behavioral difficulties and PGS of neuroticism including parental education as a covariate 72](#_Toc180693836)

[Table S50 Full model output for best fitting latent growth model including trajectories of emotional difficulties and PGS of bipolar disorder including parental education as a covariate 73](#_Toc180693837)

[Table S51 Full model output for best fitting latent growth model including trajectories of behavioral difficulties and PGS of bipolar disorder including parental education as a covariate 74](#_Toc180693838)

[Table S52: Relative odds of any emotional disorder given assignment to specific developmental profile including parental education as a covariate 75](#_Toc180693839)

[Table S53: Relative odds of assignment to specific developmental profile per standard deviation increase in polygenic score for depression including parental education as a covariate. 76](#_Toc180693840)

[Table S54: Relative odds of assignment to specific developmental profile per standard deviation increase in polygenic score for anxiety including parental education as a covariate. 76](#_Toc180693841)

[Table S55: Relative odds of assignment to specific developmental profile per standard deviation increase in polygenic score for bipolar disorder including parental education as a covariate. 77](#_Toc180693842)

[Table S56: Relative odds of assignment to specific developmental profile per standard deviation increase in polygenic score for neuroticism including parental education as a covariate. 77](#_Toc180693843)

[SUPPLEMENTARY REFERENCES 78](#_Toc180693844)

# Figure S1 Flow diagram for study sample


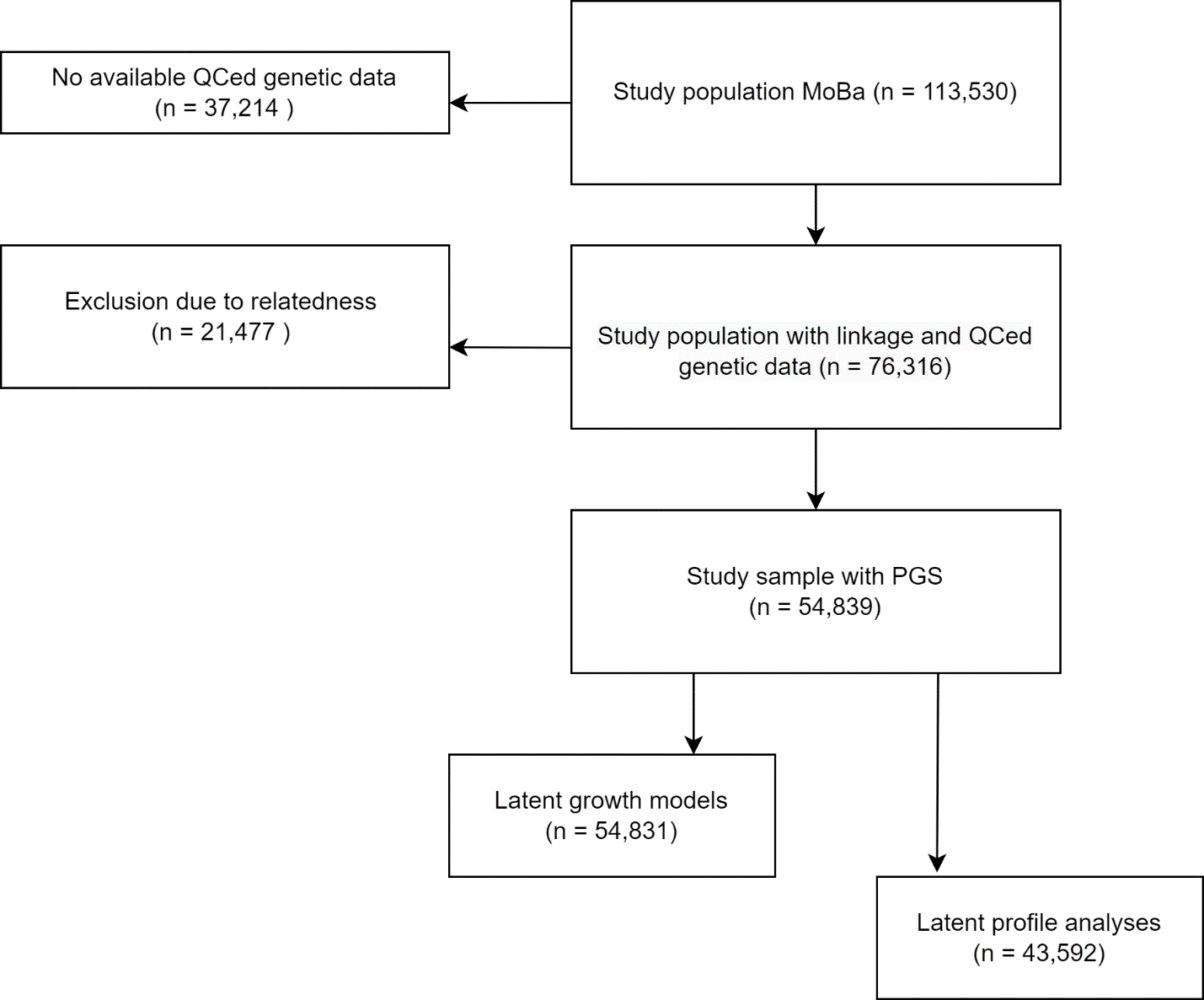


# Table S1 Questions included in each childhood emotional and behavioral difficulty measure.

| **Instrument** | **Mental health difficulty measures** | **Item level data for each measure** |
| --- | --- | --- |
| **CBCL** | **To what extent are the following statements true of your child’s behavior during the last two months?** | |
|  | Emotional difficulties | - Disturbed by any change in routine - Clings to adults or too dependent - Gets too upset when separated from parents - Too fearful or anxious - Doesn’t eat well |
|  | Behavioral difficulties | - Can’t concentrate, can’t pay attention for long - Cant sit still, restless or overactive - Hits others - Doesn’t seem to feel guilty after misbehaving - Gets in many fights - Quickly shifts from one activity to another - Defiant - Punishment doesn’t change his/her behaviour |
| **SMFQ** | **Mark how true each item has been for your child during the two last weeks.** | |
|  | Depressive symptoms 8yrs | - Felt miserable or unhappy - Felt so tired that s/he just sat around and did nothing - Was very restless - Didn’t enjoy anything at all - Felt s/he was no good anymore - Cried a lot - Hated him/herself - Thought s/he could never be as good as other kids - Felt lonely - Thought nobody really loved him/her - Felt s/he was a bad person - Felt s/he did everything wrong - Found it hard to think/concentrate |
| **SCARED** | **The questions below are about how your child have felt or behaved recently** | |
|  | Anxiety symptoms 8yrs | - My child gets really frightened for no reason at all - My child is afraid to be alone in the house - People tell my child that he/she worries too much - My child is scared to go to school - My child is shy |
| **RS-DBD** | **Mark the box that best describes your child’s behavior during the last 12 months/last year** | |
|  | Hyperactivity 8yrs | - Fidgets with hands or feet or squirms in seat (sits uneasily) - Leaves seat in classroom or in other situations in which remaining seated is expected (e.g. at the table or in group gathering) - Runs about or climbs excessively in situations in which it is inappropriate - Has difficulty playing or engaging in leisure activities quietly - Is “on the go” or acts as if “driven by a motor” - Talks excessively - Blurts out answers before questions have been completed - Has difficulty awaiting turn - Interrupts or intrudes on others, such as in conversation or play |
|  | Inattention 8yrs | - Fails to give close attention to details or makes careless mistakes in schoolwork - Has difficulty sustaining attention in tasks or play activities - Does not seem to listen when spoken to directly - Does not follow through on instructions and fails to finish school work, chores or duties (not due to oppositional behaviour or failure to understand instructions) - Has difficulty organizing tasks and activities - Avoids, dislikes or is reluctant to engage in tasks that require sustained mental effort (such as schoolwork or homework) - Loses things necessary for tasks or activities (pencils, books, toys) - Is easily distracted - Is forgetful in daily activities |
|  | Oppositional defiant disorder symptoms 8yrs | - Loses temper (tantrums) - Argues with adults - Actively defies or refuses to comply with adults’ requests or rules - Deliberately annoys people - Blames others for his/her mistakes or misbehaviour - Is touchy or easily annoyed by others - Is angry and resentful - Is spiteful or vindictive |
|  | Conduct disorder symptoms 8yrs | - Bullies, threatens or intimidates others - Initiates physical fights - Has been physically cruel to others - Has harassed or injured animals physically - Has stolen items of nontrivial value without confronting a victim (e.g. shoplifting) - Has deliberately destroyed other’s property - Has been truant from school - Has used an object that can cause serious physical harm to others (e.g. a bat, stone, knife, heavy toy) |

***Note:* CBCL=** The Child Behavior Checklist (Achenbach & Ruffle, 2000). **SMFQ=** Short Mood and Feelings Questionnaire (Angold, Costello, Messer, & Pickles, 1995). **SCARED=** Screen for Child Anxiety Related Disorders (Birmaher et al., 1999). **RS-DBD=** Rating Scale for Disruptive Behaviour Disorders (RS-DBD) (Silva et al., 2005).

# Table S2. Key descriptives for original GWAS:

| **GWAS** | ***N***  **cases** | ***N* controls** | ***N***  **total** | ***N***  **loci** | **h^2^_snp_** | **Phenotyping strategy** |
| --- | --- | --- | --- | --- | --- | --- |
| Depression (Howard et al., 2019) | 246,363 | 561,190 | 807,553 | 101 | 0.089^1^ | Meta-analysis of three studies:  1) self-reported past help-seeking for problems with “nerves, anxiety, tension or depression” (termed “broad depression”) (Howard et al., 2018)  2) self-reported diagnosis of or presence of depression. (Hyde et al., 2016)  3) life time diagnosis of depression (or anxiety) from registry or by clinical interview. (Wray et al., 2018) |
| Anxiety (Purves et al., 2020) | 25,453 | 58,113 | 83,566 | 5 | 0.260^2^ | 1) self-report of a lifetime professional diagnosis of one of the core five anxiety disorders (generalized  anxiety disorder, social phobia, panic disorder, agoraphobia or specific phobia    Or  2) meeting criteria for a likely lifetime diagnosis of DSM-IV generalized anxiety disorder based on anxiety questions from the Composite International Diagnostic Interview (CIDI) Short-form questionnaire. |
| Bipolar disorder (Mullins et al., 2021) | 40,939 | 366,226 | 407,165 | 64 | 0.186^1^ | Cases were required to meet international consensus criteria (DSM-IV, ICD-9 or ICD-10) for a lifetime diagnosis of BD, established using structured diagnostic instruments from assessments by trained interviewers, clinician-administered checklists, medical record review or self-report of BD during a nurse interview. |
| Neuroticism (Nagel et al., 2018) |  | | 372,903 | 136 | 0.100^3^ | 1) 12 dichotomous (yes or no) items of the Eysenck Personality Questionnaire Revised Short Form (EPQ-RS)   - Does your mood often go up and down? - Do you ever feel ‘just miserable’ for no reason? - Are you an irritable person? - Are your feelings easily hurt? - Are your feelings easily hurt? - Do you often feel ‘fed-up’? - Would you call yourself a nervous person? - Are you a worrier? - Would you call yourself tense or ‘highly strung’? - Do you worry too long after an embarrassing experience? - Do you suffer from ‘nerves’? - Do you often feel lonely? - Are you often troubled by feelings of guilt? |

*Note: 1*) SNP heritability measured on liability scale using LDSC regression. 2) SNP heritability based on variance component analyses conducted in BOLT-LMM converted to liability threshold. 3) Based on linkage disequilibrium score regression, estimated on liability scale. 4) Based on linkage disequilibrium score regression.

# Appendix S1. Detailed description of PGS calculation and evaluation procedure

*Detailed description of PGS calculation using LDPred2*

SNP data from the unrelated children (*n* = 54,839) in MoBa were extracted and converted from PLINK .bed files to the bigsnpr R package “bigSNP” (Privé, Aschard, Ziyatdinov & Blum, 2018) format (using the bigsnpr::snp_readBed function), and imputed in-place (using bigsnpr::snp_fastImputeSimple function with mode “mean0”). Duplicate SNPs were subsequently removed. Polygenic scores were estimated using the LDPred2 “auto” model (Privé, Arbel & Vilhjálmsson, 2020), as implemented using the R package bigsnpr via a set of custom R scripts and software containers (Akdeniz, Frei, Hagen, Filiz, Karthikeyan, 2022; Frei, Jangmo, Hagen, Akdeniz, Zetterberg, Filiz & Shorter, 2023). The Ldpred2 “auto” model is free of hyperparameters. We used the LD reference for HapMAP3+ (Privé, 2022).

*PGS evaluation*

To evaluate the PGS for each trait (PGS_trait_), we fit generalized linear models (GLM) on the form

$$y_{\mathrm{trait}}\sim1+\mathrm{PGS}_{\mathrm{trait}}+SEX+\mathrm{BATCH}_{\mathrm{geno}}+\mathrm{Year}_{\mathrm{birth}}+\mathrm{PC}_{1}+\mathrm{PC}_{2}+\ldots+\mathrm{PC}_{10}.$$

Here, $y_{\mathrm{trait}}$ is the binary trait in terms of bipolar disorder (BD), anxiety (ANX), and depression (DEP), or neuroticism score (NEUR) which were treated as a continuous trait. The variable $\mathrm{SEX}$ denotes biological sex, $\mathrm{BATCH}_{\mathrm{geno}}$ the genotypic batch number, Year_birth_ the year of birth, and $\mathrm{PC}_{n}$ the $n$-th PC. SEX, BATCH_geno_ and Year_birth_ were treated as categorical variables (one-hot-encoded). The corresponding null models excluding effects of PGS_trait_ were fitted using GLMs on the form

$$y_{\mathrm{trait}}\sim1+SEX+\mathrm{BATCH}_{\mathrm{geno}}+\mathrm{Year}_{\mathrm{birth}}+\mathrm{PC}_{1}+\mathrm{PC}_{2}+\ldots+\mathrm{PC}_{10}.$$

The models were fitted using the “glm” function in R using the binomial family with logit links for the binary traits. The continuous trait models used the Gaussian family. Each logistic regression model’s goodness of fit was evaluated in terms of the Nagelkerke pseudo coefficient of determination (R^2^) (Nagelkerke, 1991), while the standard coefficient of determination (R^2^) was used for the continuous trait. The results are summarized in Table S2.

*Standardized residuals for PGS*

We calculated the residual PGS (for each trait) $\mathrm{PG}S_{\mathrm{trait}}^{\mathrm{res}}$ as:

$$\mathrm{PG}S_{\mathrm{trait}}^{\mathrm{res}}=PGS_{\mathrm{trait}}-X\hat{\beta},$$

where $\mathrm{PG}S_{\mathrm{trait}}$ is the standardized polygenic score from LDpred2, and $\hat{\beta}$ are the estimated linear regression coefficients obtained from regressing the effect of covariates $X$ (genotyping batch and the first ten genetic principal components) on $\mathrm{PG}S_{\mathrm{trait}}$:

$$\mathrm{PG}S_{\mathrm{trait}}\sim1+\mathrm{BATC}H_{\mathrm{geno}}+PC_{1}+\ldots+PC_{10}$$

# Table S3 PGS relation to depression, anxiety and bipolar disorder in our sample

| **PGS** | **OR (95% CI)** | ***p*-value** | **R^2^ PGS model** | **R^2^ null model** |
| --- | --- | --- | --- | --- |
| **DEP** | 1.43 (1.36, 1.49) | <0.001 | 0.088 | 0.072 |
| **ANX** | 1.24 (1.19, 1.29) | <0.001 | 0.055 | 0.048 |
| **BD** | 1.58 (1.32, 1.90) | <0.001 | 0.078 | 0.063 |

**Note:** Odds Ratio (OR) reported for diagnostic outcome. P-value corresponds to OR for diagnostic outcome. PGS_DEP_ is tested against depressive disorder diagnosis (*n* = 1,954), PGS_ANX_ is tested against anxiety disorder diagnosis (*n* = 2,853), PGS_BD_ is tested against bipolar disorder diagnosis (*n* = 118). ANX = anxiety, DEP = depression, BD = bipolar disorder.

# Table S4 PGS relation to neuroticism in our sample

| **PGS** | $\beta$ **(95% CI)** | ***p*-value** | **R^2^ PGS-model** | **R^2^ null model** |
| --- | --- | --- | --- | --- |
| **NEUR** | 0.26 (0.21, 0.31) | <0.001 | 0.015 | 0.011 |

**Note:** The standardized $\beta$ coefficient is reported for the maternally reported level of neuroticism at 8 years (continuous measure). P-value corresponds to $\beta$.NEUR = neuroticism.

# Appendix S2. Lavaan model syntax for the latent growth models

*Lavaan script for growth models*

#adapted from: <https://github.com/psychgen/scz-prs-psychopathol-dev/blob/master/scripts/01.1_specify_growth_models.R>

by_cat=birth year, pgs= polygenic score adjusted for 10 first principal components and batch effects.

**#Latent growth model without PGS to assess adequacy of linear model in sample**

basic <-

'

#Growth parameters (latent variables)

i1 =~ 1*ytime1 + 1*ytime2 + 1*ytime3

s1 =~ 0*ytime1 + 1.5*ytime2 + 3.5*ytime3

#Obs variable variances

ytime1 ~~ ytime1

ytime2 ~~ ytime2

ytime3 ~~ ytime3

#Growth parameter (co)variances

i1 ~~ i1

s1 ~~ s1

i1 ~~ s1

#Obs variable intercepts (fixed to 0)

ytime1 ~ 0*1 + sex + by_cat

ytime2 ~ 0*1 + sex + by_cat

ytime3 ~ 0*1 + sex + by_cat

#Growth parameter intercepts (freely estimated)

i1 ~ 1

s1 ~ 1

'

**# PGS effect on age specific residuals**

**#** the age-specific residuals represent the variance in emotional and behavioral difficulties measured at each time-point (1.5, 3 and 5 years of age) after accounting for variation explained by the growth parameters (intercept and slope). Thus this model test influence of polygenic scores on the time-specific variation in emotional and behavioral difficulties.

pgs_residuals <-

'

#Growth parameters (latent variables)

i1 =~ 1*ytime1 + 1*ytime2 + 1*ytime3

s1 =~ 0*ytime1 + 1.5*ytime2 + 3.5*ytime3

#Obs variable variances

ytime1 ~~ ytime1

ytime2 ~~ ytime2

ytime3 ~~ ytime3

#Growth parameter (co)variances

i1 ~~ i1

s1 ~~ s1

i1 ~~ s1

#Obs variable intercepts (fixed to 0)

ytime1 ~ 0*1 + pgs + sex + by_cat

ytime2 ~ 0*1 + pgs + sex + by_cat

ytime3 ~ 0*1 + pgs + sex + by_cat

#Growth parameter intercepts (freely estimated)

i1 ~ 1

s1 ~ 1

'

**# PGS on growth factors (intercept and slope)**

pgs_gf <-

'

#Growth parameters (latent variables)

i1 =~ 1*ytime1 + 1*ytime2 + 1*ytime3

s1 =~ 0*ytime1 + 1.5*ytime2 + 3.5*ytime3

#Obs variable variances

ytime1 ~~ ytime1

ytime2 ~~ ytime2

ytime3 ~~ ytime3

#Growth parameter (co)variances

i1 ~~ i1

s1 ~~ s1

i1 ~~ s1

#Obs variable intercepts (fixed to 0)

ytime1 ~ 0*1 + sex + by_cat

ytime2 ~ 0*1 + sex + by_cat

ytime3 ~ 0*1 + sex + by_cat

#Growth parameter intercepts (freely estimated)

i1 ~ 1 + pgs

s1 ~ 1 + pgs

'

**# PGS on intercept only**

pgs_intercept <-

'

#Growth parameters (latent variables)

i1 =~ 1*ytime1 + 1*ytime2 + 1*ytime3

s1 =~ 0*ytime1 + 1.5*ytime2 + 3.5*ytime3

#Obs variable variances

ytime1 ~~ ytime1

ytime2 ~~ ytime2

ytime3 ~~ ytime3

#Growth parameter (co)variances

i1 ~~ i1

s1 ~~ s1

i1 ~~ s1

#Obs variable intercepts (fixed to 0)

ytime1 ~ 0*1 + sex + by_cat

ytime2 ~ 0*1 + sex + by_cat

ytime3 ~ 0*1 + sex + by_cat

#Growth parameter intercepts (freely estimated)

i1 ~ 1 + pgs

s1 ~ 1 + 0*pgs

'

**# PGS effect on slope only**

pgs_slope <-

'

#Growth parameters (latent variables)

i1 =~ 1*ytime1 + 1*ytime2 + 1*ytime3

s1 =~ 0*ytime1 + 1.5*ytime2 + 3.5*ytime3

#Obs variable variances

ytime1 ~~ ytime1

ytime2 ~~ ytime2

ytime3 ~~ ytime3

#Growth parameter (co)variances

i1 ~~ i1

s1 ~~ s1

i1 ~~ s1

#Obs variable intercepts (fixed to 0)

ytime1 ~ 0*1 + sex + by_cat

ytime2 ~ 0*1 + sex + by_cat

ytime3 ~ 0*1 + sex + by_cat

#Growth parameter intercepts (freely estimated)

i1 ~ 1 + 0*pgs

s1 ~ 1 + pgs

'

**# no PGS effects (PGS fixed to null)**

lgm_nopgs <-

'

#Growth parameters (latent variables)

i1 =~ 1*ytime1 + 1*ytime2 + 1*ytime3

s1 =~ 0*ytime1 + 1.5*ytime2 + 3.5*ytime3

#Obs variable variances

ytime1 ~~ ytime1

ytime2 ~~ ytime2

ytime3 ~~ ytime3

#Growth parameter (co)variances

i1 ~~ i1

s1 ~~ s1

i1 ~~ s1

#Obs variable intercepts (fixed to 0)

ytime1 ~ 0*1 + sex + by_cat

ytime2 ~ 0*1 + sex + by_cat

ytime3 ~ 0*1 + sex + by_cat

#Growth parameter intercepts (freely estimated)

i1 ~ 1 + 0*pgs

s1 ~ 1 + 0*pgs

# Appendix S3. Detailed description of the stepwise growth model selection procedure

*Detailed description of stepwise model selection procedure*

1. Define the five models: PGS effect on age-specific residuals (“pgs_residuals”), PGS effect on both intercept and slope growth factors (“pgs_gf”), PGS effect on intercept growth factor (“pgs_intercept”), PGS effect on slope growth factor (“pgs_slope”), and PGS effect fixed to null (“lgm_nopgs).
2. Compare pgs_gf with pgs_residuals; if pgs_gf is not worse performing than pgs_residuals, select pgs_gf and move to step 3; otherwise select pgs_residuals as the final model.
3. If pgs_gf is selected at step 2, formally compare to pgs_intercept and pgs_slope respectively; if both are significantly worse performing, select pgs_gf as the final model; otherwise select the model with the lower AIC from pgs_intercept and pgs_slope and move to step 4.
4. If pgs_intercept or pgs_slope is selected at step 3, compare the selected model with lgm_nopgs; if lgm_nopgs is not worse performing, select it as the final model; otherwise, select the comparator model (either pgs_intercept or pgs_slope) as the final model.

We used the change in AIC and *p* value from Chi-square test to assess if the models were not worse performing.

*Explanation of logic behind the stepwise selection procedure*

The models are compared in a stepwise manner starting with the most complex model (PGS-residuals) and ending with the least complex model (lgm-nopgs). In the first step of growth model comparisons, we assess if a model with PGS regressed on latent growth factors (assuming all influence of PGS on the difficulties are trough the latent growth factors, pgs_gf) is better or equally as well performing as a model where PGS effects regressed directly on the repeated measures, e.g allowing PGS to account for variation in the individual age specific difficulty measures). If we consider the PGS-gf model to be equally well performing, we consider the age-specific effects as small/negligible and can continue with the model with pgs regressed on the latent growth factors. Further, we assess if a model with PGS effect on only baseline level (pgs_intercept) or rate of change (pgs_slope) is better or equally well performing as a model with influence on both baseline and rate of change in symptoms (pgs_gf). If one of these are at least equally well performing and the other is not, we consider the tested PGS effect of the worse performing model small/negligible and conclude that the main effect is on baseline or rate of chance in difficulties. Last, we assess if PGS effect on the selected model is better or equally well performing as a model with no PGS effect. If we find that the two models perform equally well we conclude that we cannot identify any PGS effect, else we conclude that the last tested model represents the main effect of PGS on the phenotype.

# Figure S2: Latent profile analysis incorporating latent growth models for developmental profiles of emotional and behavioral difficulties and polygenic scores.

**
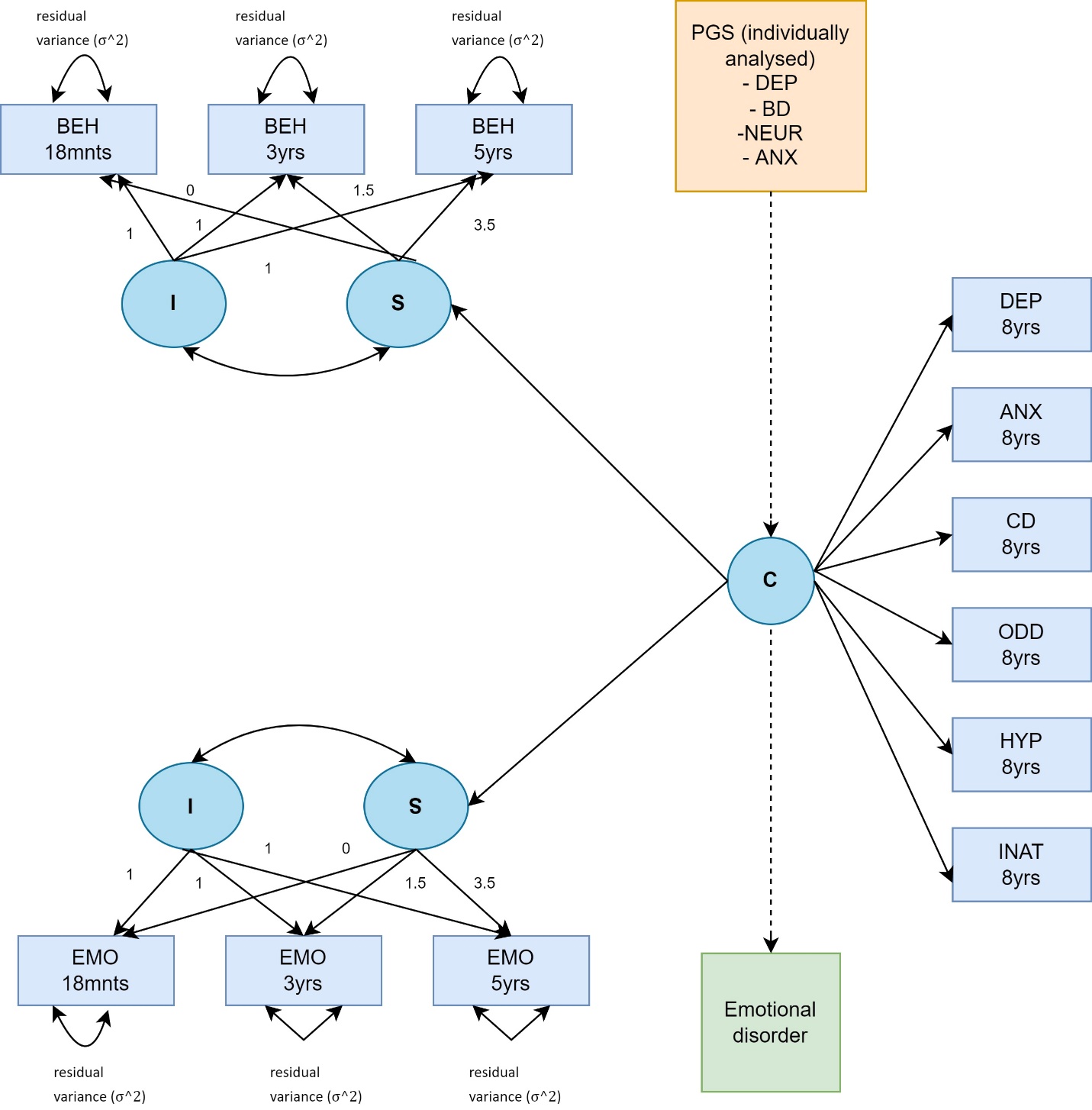
**

**Note**: Illustration of methods for developmental profiles incorporating latent growth models in 3-step maximum likelihood latent profile analysis. Boxes represent observed variables and circles model-estimated latent variables; I = intercept factor, loads equally on observed variables at all waves; C = categorical latent variable, subdividing the sample into a specified number of classes according to values: S = slope factor with loadings 0(18mnts), 1.5(3yrs), and 3.5(5yrs), corresponding to temporal distance between the measurements; BEH = Behavioral difficulties; EMO = Emotional difficulties; DEP= Depressive symptoms; ANX = Anxiety symptoms; CD= Conduct disorder symptoms; ODD = Oppositional defiant disorder symptoms; HYP = Hyperactivity; INAT= Inattention. Emotional/ behavioral intercept/slope variables and 8-year observed variables are intercorrelated within class (paths omitted from diagram for clarity) in step 1 of the latent profile analysis. Polygenic score for depression, anxiety, bipolar disorder and neuroticism is included as covariates (separately) and emotional disorder is included as distal outcome in step 3 in model-specification (indicated by dashed line).

For further methodological details on latent growth models see “Genetic liability for schizophrenia and childhood psychopathology in the general population” by L.J. Hannigan in Schizophrenia Bulletin Volume 47, Issue 4, July 2021, Pages 1179–1189 (Hannigan, et al., 2021) and “Childhood temperamental, emotional, and behavioral characteristics associated with mood and anxiety disorders in adolescence: A prospective study” by N.R. Bakken in Acta Psychiatrica Scandinavica Volume 147, Issue 2, February 2023. Pages 217-228 (Bakken, et.al, 2023).

# Table S5 Ordinal Cronbach’s alpha for each childhood emotional and behavioral difficulty measure

| **Emotional and behavioral difficulty measure** | **Ordinal Cronbach alpha** |
| --- | --- |
| Emotional problems 1.5yrs | 0.64 |
| Emotional problems 3yrs | 0.68 |
| Emotional problems 5 yrs | 0.73 |
| Behavioral problems 1.5yrs | 0.69 |
| Behavioral problems 3yrs | 0.77 |
| Behavioral problems 5 yrs | 0.81 |
| Depressive symptoms 8yrs | 0.92 |
| Anxiety symptoms 8yrs | 0.76 |
| Hyperactivity 8yrs | 0.92 |
| Inattention 8yrs | 0.92 |
| Oppositional defiant disorder symptoms 8yrs | 0.91 |
| Conduct disorder symptoms 8yrs | 0.88 |

Table S6: Depression PGS. Linear association between standardized PGS score and standardized score of emotional and behavioral difficulties.

| Characteristic | $\beta$ | 95% CI^1^ | *p*-value | *p-FDR*^2^ |
| --- | --- | --- | --- | --- |
| Emotional difficulties 1.5 yrs | 0.02 | 0.01, 0.03 | <0.001 | <0.001 |
| Emotional difficulties 3yrs | 0.02 | 0.00, 0.03 | 0.005 | 0.006 |
| Emotional difficulties 5 yrs | 0.03 | 0.01, 0.04 | <0.001 | <0.001 |
| Behavioral difficulties 1.5yrs | 0.01 | 0.00, 0.02 | 0.004 | 0.006 |
| Behavioral difficulties 3yrs | 0.03 | 0.02, 0.04 | <0.001 | <0.001 |
| Behavioral difficulties 5 yrs | 0.05 | 0.03, 0.06 | <0.001 | <0.001 |
| Depressive symptoms 8yrs | 0.06 | 0.04, 0.07 | <0.001 | <0.001 |
| Anxiety symptoms 8yrs | 0.03 | 0.02, 0.04 | <0.001 | <0.001 |
| Inattention 8yrs | 0.04 | 0.02, 0.05 | <0.001 | <0.001 |
| Oppositional defiant disorder symptoms 8yrs | 0.05 | 0.03, 0.06 | <0.001 | <0.001 |
| Hyperactivity 8yrs | 0.03 | 0.02, 0.05 | <0.001 | <0.001 |
| Conduct disorder symptoms 8yrs | 0.04 | 0.03, 0.06 | <0.001 | <0.001 |

| ^1^CI = Confidence Interval |
| --- |
| ^2^False discovery rate correction for multiple testing using Benjamini-Hochberg false discovery rate procedure |

# **Table S7: Neuroticism PGS. Linear association between standardized PGS score and standardized score of emotional and behavioral difficulties**.

| Characteristic | $\beta$ | 95% CI^1^ | p-value | p-FDR^2^ |  |  |
| --- | --- | --- | --- | --- | --- | --- |
| Emotional difficulties 1.5yrs | 0.04 | 0.03, 0.05 | <0.001 | <0.001 |  |  |
| Emotional difficulties 3yrs | 0.04 | 0.03, 0.05 | <0.001 | <0.001 |  |  |
| Emotional difficulties 5 yrs | 0.04 | 0.03, 0.06 | <0.001 | <0.001 |  |  |
| Behavioral difficulties 1.5yrs | 0.03 | 0.02, 0.04 | <0.001 | <0.001 |  |  |
| Behavioral difficulties 3yrs | 0.03 | 0.02, 0.04 | <0.001 | <0.001 |  |  |
| Behavioral difficulties 5 yrs | 0.04 | 0.03, 0.05 | <0.001 | <0.001 |  |  |
| Depressive symptoms 8yrs | 0.07 | 0.05, 0.08 | <0.001 | <0.001 |  |  |
| Anxiety symptoms 8yrs | 0.05 | 0.04, 0.07 | <0.001 | <0.001 |  |  |
| Inattention 8yrs | 0.03 | 0.02, 0.04 | <0.001 | <0.001 |  |  |
| Oppositional defiant disorder symptoms 8yrs | 0.04 | 0.03, 0.06 | <0.001 | <0.001 |  |  |
| Hyperactivity 8yrs | 0.02 | 0.01, 0.04 | <0.001 | 0.001 |  |  |
| Conduct disorder symptoms 8yrs | 0.02 | 0.01, 0.03 | 0.007 | 0.010 |  |  |
| ^1^CI = Confidence Interval | | | | | | |
| ^2^False discovery rate correction for multiple testing using Benjamini-Hochberg false discovery rate procedure | | | | | | |

# Table S8: Anxiety disorder PGS. Linear association between standardized PGS score and standardized score of emotional and behavioral difficulties.

| Characteristic | $\beta$ | 95% CI^1^ | p-value | p-FDR^2^ |  |  |
| --- | --- | --- | --- | --- | --- | --- |
| Emotional difficulties 1.5yrs | 0.01 | 0.00, 0.02 | 0.026 | 0.033 |  |  |
| Emotional difficulties 3yrs | 0.02 | 0.01, 0.03 | 0.003 | 0.004 |  |  |
| Emotional difficulties 5 yrs | 0.03 | 0.02, 0.04 | <0.001 | <0.001 |  |  |
| Behavioral difficulties 1.5yrs | 0.01 | 0.00, 0.02 | 0.058 | 0.072 |  |  |
| Behavioral difficulties 3yrs | 0.02 | 0.01, 0.03 | 0.004 | 0.005 |  |  |
| Behavioral difficulties 5 yrs | 0.03 | 0.01, 0.04 | <0.001 | <0.001 |  |  |
| Depressive symptoms 8yrs | 0.04 | 0.03, 0.05 | <0.001 | <0.001 |  |  |
| Anxiety symptoms 8yrs | 0.03 | 0.01, 0.04 | <0.001 | <0.001 |  |  |
| Inattention 8yrs | 0.02 | 0.01, 0.04 | <0.001 | 0.001 |  |  |
| Oppositional defiant disorder symptoms 8yrs | 0.03 | 0.01, 0.04 | <0.001 | <0.001 |  |  |
| Hyperactivity 8yrs | 0.02 | 0.01, 0.04 | <0.001 | 0.001 |  |  |
| Conduct disorder symptoms 8yrs | 0.02 | 0.01, 0.03 | 0.003 | 0.004 |  |  |
| ^1^CI = Confidence Interval | | | | | | |
| ^2^False discovery rate correction for multiple testing using Benjamini-Hochberg false discovery rate procedure | | | | | | |

Table S9: Bipolar disorder PGS. Linear association between standardized PGS score and standardized score of emotional and behavioral difficulties.

| Characteristic | $\beta$ | 95% CI^1^ | p-value | p-FDR^2^ |
| --- | --- | --- | --- | --- |
| Emotional difficulties 1.5yrs | -0.01 | -0.02, 0.00 | 0.254 | 0.284 |
| Emotional difficulties 3yrs | 0.00 | -0.02, 0.01 | 0.394 | 0.420 |
| Emotional difficulties 5 yrs | -0.01 | -0.02, 0.01 | 0.216 | 0.247 |
| Behavioral difficulties 1.5yrs | -0.01 | -0.02, 0.00 | 0.108 | 0.126 |
| Behavioral difficulties 3yrs | -0.01 | -0.02, 0.01 | 0.312 | 0.340 |
| Behavioral difficulties 5 yrs | 0.00 | -0.01, 0.02 | 0.582 | 0.594 |
| Depressive symptoms 8yrs | 0.02 | 0.01, 0.03 | 0.002 | 0.002 |
| Anxiety symptoms 8yrs | 0.00 | -0.01, 0.02 | 0.707 | 0.707 |
| Inattention 8yrs | 0.00 | -0.01, 0.02 | 0.495 | 0.516 |
| Oppositional defiant disorder symptoms 8yrs | 0.03 | 0.02, 0.04 | <0.001 | <0.001 |
| Hyperactivity 8yrs | 0.02 | 0.01, 0.03 | 0.002 | 0.003 |
| Conduct disorder symptoms 8yrs | 0.03 | 0.02, 0.04 | <0.001 | <0.001 |

| ^1^CI = Confidence Interval |
| --- |
| ^2^False discovery rate correction for multiple testing using Benjamini-Hochberg false discovery rate procedure |

# Table S10 Model fit for basic linear latent growth models across early childhood

| **Measurement** | **CFI** | **TLI** | **RMSEA (95% CI)** | **SRMR** | **chisq** | **p-value chisq** | **DF** |
| --- | --- | --- | --- | --- | --- | --- | --- |
| Emotional difficulties | 0.993 | 0.939 | 0.032 (0.025, 0.039) | 0.01 | 56.676 | <0.001 | 1 |
| Behavioral difficulties | 0.992 | 0.927 | 0.044 (0.037, 0.051) | 0.013 | 105.257 | <0.001 | 1 |

**Note:** CFI= Comparative fit index; TLI=Tucker Lewis Index and RMSEA=Root Mean Square Error of Approximation. SRMR=Standardized Root Mean Square Residual. Chisq= chi-square (χ2) statistic. DF= Degrees of freedom.

# Figure S3 Trajectories of emotional difficulties across early childhood (1.5, 3 and 5 years)

*
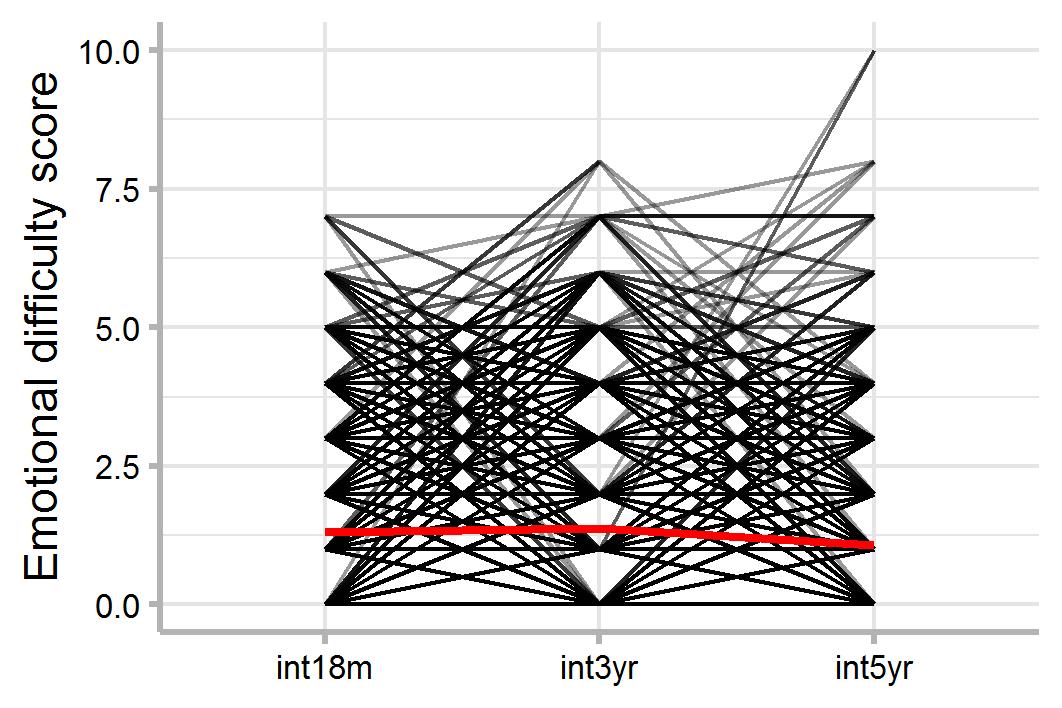
*

**Note**: Plots illustrating trajectories of emotional difficulties (1.5, 3 and 5 years) for 10,000 randomly selected individuals, red line highlight sample mean. Int18m= emotional problems at 1.5 years. Int3yr=emotional problems at 3 years. Int5years=emotional problems at 5 years.

# Figure S4 Trajectories of behavioral difficulties across early childhood (1.5, 3 and 5 years)

*
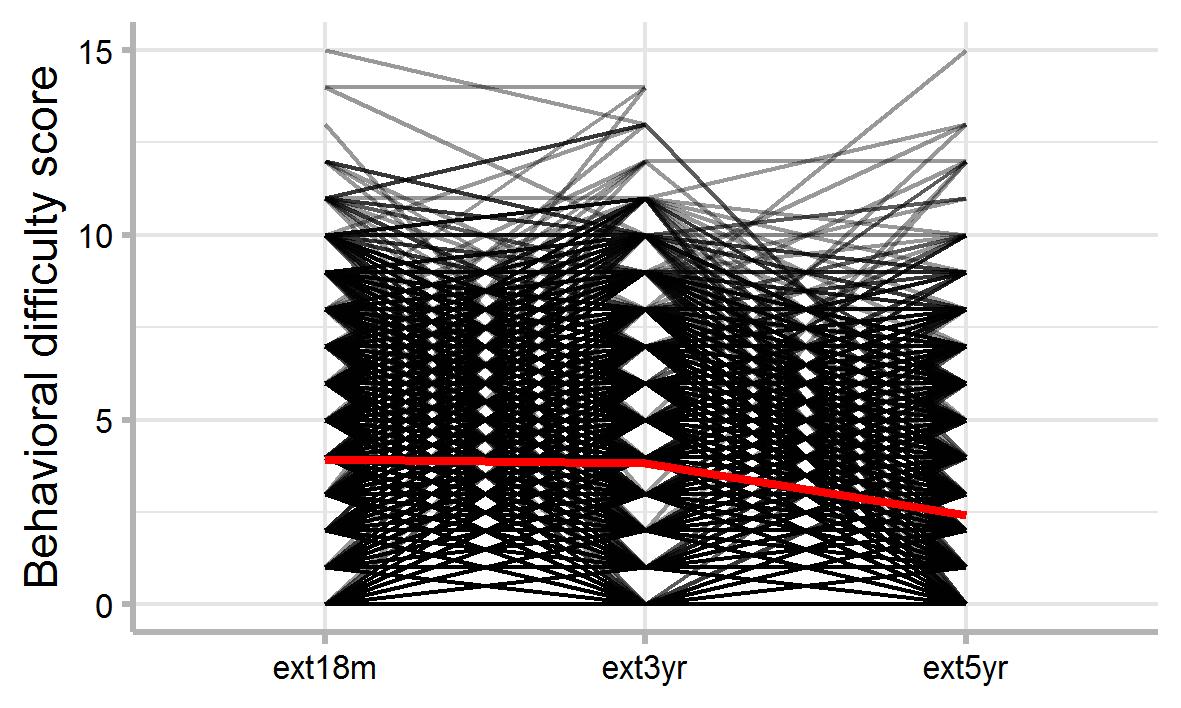
*

**Note**: Plots illustrating trajectories of behavioral difficulties (1.5, 3 and 5 years) for 10,000 randomly selected individuals, red line highlight sample mean. Ext 18m= behavioral problems at 1.5 years. Ext3yr= behavioral problems at 3 years. Ext5yr=behavioral problems at 5 years.

# Table S11 Evaluating best fitting latent growth model for emotional and behavioral difficulties for PGS of depression

| Childhood difficulty | Model | Df | AIC | Chisq.diff | P(>Chisq) |
| --- | --- | --- | --- | --- | --- |
| **Emotional difficulties** |  |  |  |  |  |
|  | Age specific PGS effects | 1 | 290246.4 |  |  |
|  | PGS effect on growth factors | 2 | 290244.7 | 0.258 | 0.611 |
|  | PGS effect on growth factors | 2 | 290244.7 |  |  |
|  | **PGS effect on intercept only** | **3** | **290243.4** | **0.650** | **0.420** |
|  | PGS effect on growth factors | 2 | 290244.7 |  |  |
|  | PGS effect on slope only | 3 | 290257.4 | 14.667 | 0.000 |
|  | PGS effect on intercept only | 3 | 290243.4 |  |  |
|  | No PGS effect | 4 | 290265.8 | 24.433 | 0.000 |
| **Behavioral difficulties** |  |  |  |  |  |
|  | Age specific PGS effects | 1 | 399123.6 |  |  |
|  | **PGS effect on growth factors** | **2** | **399122.6** | **1.008** | **0.315** |
|  | PGS effect on growth factors | 2 | 399122.6 |  |  |
|  | PGS effect on intercept only | 3 | 399143.0 | 22.380 | 0.000 |
|  | PGS effect on growth factors | 2 | 399122.6 |  |  |
|  | PGS effect on slope only | 3 | 399131.9 | 11.280 | 0.001 |
|  | PGS effect on growth factors | 2 | 399122.6 |  |  |
|  | No PGS effect | 4 | 399184.4 | 65.842 | 0.000 |

**Note:** Df= Degrees of Freedom, AIC= First-order Akaike Information Criteria, Chisq.diff= Difference in Chi-Square Value. P(>Chisq) = P-value for Chi-Square Test. The models are compared in a step-wise manner, thus in this table each model is compared to the model listed immediately above. The final selected model is highlighted in bold.

# Table S12 Evaluating best fitting latent growth model for emotional and behavioral difficulties for PGS of anxiety

| Childhood difficulty | Model | Df | AIC | Chisq.diff | P(>Chisq) |
| --- | --- | --- | --- | --- | --- |
| **Emotional difficulties** |  |  |  |  |  |
|  | Age specific PGS effects | 1 | 290253.3 |  |  |
|  | **PGS effect on growth factors** | **2** | **290251.3** | **0.019** | **0.889** |
|  | PGS effect on growth factors | 2 | 290251.3 |  |  |
|  | PGS effect on intercept only | 3 | 290253.4 | 4.085 | 0.043 |
|  | PGS effect on growth factors | 2 | 290251.3 |  |  |
|  | PGS effect on slope only | 3 | 290254.2 | 4.948 | 0.026 |
|  | PGS effect on growth factors | 2 | 290251.3 |  |  |
|  | No PGS effect | 4 | 290265.8 | 18.502 | 0.000 |
| **Behavioral difficulties** |  |  |  |  |  |
|  | Age specific PGS effects | 1 | 399172.7 |  |  |
|  | **PGS effect on growth factors** | **2** | **399170.9** | **0.157** | **0.692** |
|  | PGS effect on growth factors | 2 | 399170.9 |  |  |
|  | PGS effect on intercept only | 3 | 399173.2 | 4.351 | 0.037 |
|  | PGS effect on growth factors | 2 | 399170.9 |  |  |
|  | PGS effect on slope only | 3 | 399173.4 | 4.472 | 0.034 |
|  | PGS effect on growth factors | 2 | 399170.9 |  |  |
|  | No PGS effect | 4 | 399184.4 | 17.543 | 0.000 |

**Note:** Df= Degrees of Freedom, AIC= First-order Akaike Information Criteria, Chisq.diff= Difference in Chi Square Value. P(>Chisq) = P-value for Chi Square Test. The models are compared in a step-wise-manner, thus in this table each model is compared to the model listed immediately above. The final selected model is highlighted in bold.

# Table S13 Evaluating best fitting latent growth model for emotional and behavioral difficulties and PGS of neuroticism

| Childhood difficulty | Model | Df | AIC | Chisq.diff | P(>Chisq) |
| --- | --- | --- | --- | --- | --- |
| **Emotional difficulties** |  |  |  |  |  |
|  | Age specific PGS effects | 1 | 290182.7 |  |  |
|  | PGS effect on growth factors | 2 | 290181.0 | 0.364 | 0.546 |
|  | PGS effect on growth factors | 2 | 290181.0 |  |  |
|  | **PGS effect on intercept only** | **3** | **290180.9** | **1.921** | **0.166** |
|  | PGS effect on growth factors | 2 | 290181.0 |  |  |
|  | PGS effect on slope only | 3 | 290232.3 | 53.270 | 0.000 |
|  | PGS effect on intercept only | 3 | 290180.9 |  |  |
|  | No PGS effect | 4 | 290265.8 | 86.841 | 0.000 |
| **Behavioral difficulties** |  |  |  |  |  |
|  | Age specific PGS effects | 1 | 399119.8 |  |  |
|  | PGS effect on growth factors | 2 | 399117.8 | 0.042 | 0.837 |
|  | PGS effect on growth factors | 2 | 399117.8 |  |  |
|  | **PGS effect on intercept only** | **3** | **399117.9** | **2.109** | **0.146** |
|  | PGS effect on growth factors | 2 | 399117.8 |  |  |
|  | PGS effect on slope only | 3 | 399157.6 | 41.791 | 0.000 |
|  | PGS effect on intercept only | 3 | 399117.9 |  |  |
|  | No PGS effect | 4 | 399184.4 | 68.494 | 0.000 |

**Note:** Df= Degrees of Freedom, AIC= First-order Akaike Information Criteria, Chisq.diff= Difference in Chi Square Value. P(>Chisq) = P-value for Chi Square Test. The models are compared in a step-wise-manner, thus in this table each model is compared to the model listed immediately above. The final selected model is highlighted in bold.

# Table S14 Evaluating best fitting latent growth model for emotional and behavioral difficulties for PGS of bipolar disorder

| Childhood difficulty | Model | Df | AIC | Chisq.diff | P(>Chisq) |
| --- | --- | --- | --- | --- | --- |
| **Emotional difficulties** |  |  |  |  |  |
|  | Age specific PGS effects | 1 | 290269.9 |  |  |
|  | PGS effect on growth factors | 2 | 290267.9 | 0.023 | 0.880 |
|  | PGS effect on growth factors | 2 | 290267.9 |  |  |
|  | PGS effect on intercept only | 3 | 290266.0 | 0.080 | 0.777 |
|  | PGS effect on growth factors | 2 | 290267.9 |  |  |
|  | PGS effect on slope only | 3 | 290266.9 | 1.009 | 0.315 |
|  | PGS effect on intercept only | 3 | 290266.0 |  |  |
|  | No PGS effect | 4 | 290265.8 | 1.790 | 0.181 |
|  | PGS effect on slope only | 3 | 290266.9 |  |  |
|  | **No PGS effect** | **4** | **290265.8** | **0.861** | **0.353** |
| **Behavioral difficulties** |  |  |  |  |  |
|  | Age specific PGS effects | 1 | 399186.0 |  |  |
|  | PGS effect on growth factors | 2 | 399184.3 | 0.338 | 0.561 |
|  | PGS effect on growth factors | 2 | 399184.3 |  |  |
|  | PGS effect on intercept only | 3 | 399184.3 | 1.969 | 0.161 |
|  | PGS effect on growth factors | 2 | 399184.3 |  |  |
|  | PGS effect on slope only | 3 | 399186.2 | 3.879 | 0.049 |
|  | PGS effect on intercept only | 3 | 399184.3 |  |  |
|  | **No PGS effect** | **4** | **399184.4** | **2.149** | **0.143** |

**Note:** Df= Degrees of Freedom, AIC= First-order Akaike Information Criteria, Chisq.diff= Difference in Chi-Square Value. P(>Chisq) = P-value for Chi-Square Test. The models are compared in a step-wise manner, thus in this table each model is compared to the model listed immediately above. The final selected model is highlighted in bold.

# Table S15 Full model output for best fitting latent growth model including trajectories of emotional difficulties and PGS of depression

Latent Variables:

Estimate Std.Err z-value P(>|z|) ci.lower ci.upper Std.lv Std.all

i1 =~

ytime1 1.000 1.000 1.000 0.892 0.734

ytime2 1.000 1.000 1.000 0.892 0.637

ytime3 1.000 1.000 1.000 0.892 0.682

s1 =~

ytime1 0.000 0.000 0.000 0.000 0.000

ytime2 1.500 1.500 1.500 0.465 0.332

ytime3 3.500 3.500 3.500 1.085 0.830

Regressions:

Estimate Std.Err z-value P(>|z|) ci.lower ci.upper Std.lv Std.all

ytime1 ~

sex 0.019 0.012 1.505 0.132 -0.006 0.043 0.019 0.008

by_cat -0.027 0.009 -3.157 0.002 -0.044 -0.010 -0.027 -0.017

ytime2 ~

sex 0.071 0.014 5.129 0.000 0.044 0.098 0.071 0.025

by_cat 0.042 0.010 4.317 0.000 0.023 0.061 0.042 0.022

ytime3 ~

sex -0.011 0.017 -0.655 0.512 -0.044 0.022 -0.011 -0.004

by_cat 0.037 0.016 2.400 0.016 0.007 0.068 0.037 0.021

i1 ~

adj_pgs 0.026 0.005 4.944 0.000 0.016 0.036 0.029 0.029

s1 ~

adj_pgs 0.000 0.000 0.000 0.000 0.000

Covariances:

Estimate Std.Err z-value P(>|z|) ci.lower ci.upper Std.lv Std.all

.i1 ~~

.s1 -0.102 0.006 -16.540 0.000 -0.114 -0.090 -0.368 -0.368

Intercepts:

Estimate Std.Err z-value P(>|z|) ci.lower ci.upper Std.lv Std.all

.ytime1 0.000 0.000 0.000 0.000 0.000

.ytime2 0.000 0.000 0.000 0.000 0.000

.ytime3 0.000 0.000 0.000 0.000 0.000

.i1 1.345 0.026 51.602 0.000 1.294 1.397 1.508 1.508

.s1 -0.091 0.014 -6.395 0.000 -0.118 -0.063 -0.292 -0.292

Variances:

Estimate Std.Err z-value P(>|z|) ci.lower ci.upper Std.lv Std.all

.ytime1 0.680 0.017 39.920 0.000 0.646 0.713 0.680 0.461

.ytime2 1.251 0.013 96.980 0.000 1.225 1.276 1.251 0.638

.ytime3 0.447 0.028 16.121 0.000 0.392 0.501 0.447 0.261

.i1 0.795 0.017 46.072 0.000 0.761 0.829 0.999 0.999

.s1 0.096 0.004 25.968 0.000 0.089 0.103 1.000 1.000

**Note:** std.err=standard error, std.lv=Standardized estimates on variances of the latent variables. Std.all= Standardized estimates based on variances of observed and latent variables. Ci.lower= lower 2.5% of 95% confidence interval. Ci.upper= upper 2.5% of 95% confidence interval. The models are run with sex coded (1,2) and by_cat (birth year categorized to three levels, coded as 1,2,3). Adj_pgs= polygenic score.

# Table S16 Full model output for best fitting latent growth model including trajectories of behavioral difficulties and PGS of depression

Latent Variables:

Estimate Std.Err z-value P(>|z|) ci.lower ci.upper Std.lv Std.all

i1 =~

ytime1 1.000 1.000 1.000 1.732 0.767

ytime2 1.000 1.000 1.000 1.732 0.714

ytime3 1.000 1.000 1.000 1.732 0.761

s1 =~

ytime1 0.000 0.000 0.000 0.000 0.000

ytime2 1.500 1.500 1.500 0.791 0.326

ytime3 3.500 3.500 3.500 1.847 0.811

Regressions:

Estimate Std.Err z-value P(>|z|) ci.lower ci.upper Std.lv Std.all

ytime1 ~

sex -0.378 0.022 -16.997 0.000 -0.422 -0.335 -0.378 -0.084

by_cat -0.144 0.015 -9.603 0.000 -0.174 -0.115 -0.144 -0.047

ytime2 ~

sex -0.190 0.024 -7.934 0.000 -0.237 -0.143 -0.190 -0.039

by_cat -0.035 0.017 -2.106 0.035 -0.068 -0.002 -0.035 -0.011

ytime3 ~

sex -0.399 0.029 -13.892 0.000 -0.455 -0.343 -0.399 -0.088

by_cat -0.118 0.026 -4.505 0.000 -0.170 -0.067 -0.118 -0.039

i1 ~

adj_pgs 0.037 0.011 3.359 0.001 0.015 0.058 0.021 0.021

s1 ~

adj_pgs 0.022 0.005 4.731 0.000 0.013 0.030 0.041 0.041

Covariances:

Estimate Std.Err z-value P(>|z|) ci.lower ci.upper Std.lv Std.all

.i1 ~~

.s1 -0.336 0.019 -18.091 0.000 -0.373 -0.300 -0.369 -0.369

Intercepts:

Estimate Std.Err z-value P(>|z|) ci.lower ci.upper Std.lv Std.all

.ytime1 0.000 0.000 0.000 0.000 0.000

.ytime2 0.000 0.000 0.000 0.000 0.000

.ytime3 0.000 0.000 0.000 0.000 0.000

.i1 4.800 0.046 105.123 0.000 4.711 4.889 2.772 2.772

.s1 -0.398 0.023 -16.938 0.000 -0.444 -0.352 -0.754 -0.754

Variances:

Estimate Std.Err z-value P(>|z|) ci.lower ci.upper Std.lv Std.all

.ytime1 2.050 0.051 40.171 0.000 1.950 2.150 2.050 0.402

.ytime2 3.255 0.036 91.515 0.000 3.186 3.325 3.255 0.553

.ytime3 1.073 0.077 13.889 0.000 0.922 1.225 1.073 0.207

.i1 2.997 0.053 56.347 0.000 2.893 3.101 1.000 1.000

.s1 0.278 0.011 25.672 0.000 0.257 0.299 0.998 0.998

**Note:** std.err=standard error, std.lv=Standardized estimates on variances of the latent variables. Std.all= Standardized estimates based on variances of observed and latent variables. Ci.lower= lower 2.5% of 95% confidence interval. Ci.upper= upper 2.5% of 95% confidence interval. The models are run with sex coded (1,2) and by_cat (birth year categorized to three levels, coded as 1,2,3). Adj_pgs= polygenic score.

# Table S17 Full model output for best fitting latent growth model including trajectories of emotional difficulties and PGS of anxiety

Latent Variables:

Estimate Std.Err z-value P(>|z|) ci.lower ci.upper Std.lv Std.all

i1 =~

ytime1 1.000 1.000 1.000 0.892 0.734

ytime2 1.000 1.000 1.000 0.892 0.637

ytime3 1.000 1.000 1.000 0.892 0.682

s1 =~

ytime1 0.000 0.000 0.000 0.000 0.000

ytime2 1.500 1.500 1.500 0.465 0.332

ytime3 3.500 3.500 3.500 1.085 0.830

Regressions:

Estimate Std.Err z-value P(>|z|) ci.lower ci.upper Std.lv Std.all

ytime1 ~

sex 0.019 0.012 1.502 0.133 -0.006 0.043 0.019 0.008

by_cat -0.028 0.009 -3.199 0.001 -0.044 -0.011 -0.028 -0.017

ytime2 ~

sex 0.071 0.014 5.124 0.000 0.044 0.098 0.071 0.025

by_cat 0.041 0.010 4.293 0.000 0.023 0.060 0.041 0.022

ytime3 ~

sex -0.011 0.017 -0.663 0.507 -0.044 0.022 -0.011 -0.004

by_cat 0.038 0.016 2.401 0.016 0.007 0.068 0.038 0.021

i1 ~

adj_pgs 0.014 0.006 2.224 0.026 0.002 0.025 0.015 0.015

s1 ~

adj_pgs 0.006 0.003 2.021 0.043 0.000 0.011 0.018 0.018

Covariances:

Estimate Std.Err z-value P(>|z|) ci.lower ci.upper Std.lv Std.all

.i1 ~~

.s1 -0.102 0.006 -16.545 0.000 -0.114 -0.090 -0.368 -0.368

Intercepts:

Estimate Std.Err z-value P(>|z|) ci.lower ci.upper Std.lv Std.all

.ytime1 0.000 0.000 0.000 0.000 0.000

.ytime2 0.000 0.000 0.000 0.000 0.000

.ytime3 0.000 0.000 0.000 0.000 0.000

.i1 1.346 0.026 51.616 0.000 1.295 1.397 1.509 1.509

.s1 -0.091 0.014 -6.403 0.000 -0.118 -0.063 -0.292 -0.292

Variances:

Estimate Std.Err z-value P(>|z|) ci.lower ci.upper Std.lv Std.all

.ytime1 0.680 0.017 39.917 0.000 0.646 0.713 0.680 0.461

.ytime2 1.251 0.013 96.977 0.000 1.225 1.276 1.251 0.638

.ytime3 0.447 0.028 16.128 0.000 0.393 0.501 0.447 0.262

.i1 0.795 0.017 46.078 0.000 0.762 0.829 1.000 1.000

.s1 0.096 0.004 25.954 0.000 0.089 0.103 1.000 1.000

**Note:** std.err=standard error, std.lv=Standardized estimates on variances of the latent variables. Std.all= Standardized estimates based on variances of observed and latent variables. Ci.lower= lower 2.5% of 95% confidence interval. Ci.upper= upper 2.5% of 95% confidence interval. The models are run with sex coded (1,2) and by_cat (birth year categorized to three levels, coded as 1,2,3). Adj_pgs= polygenic score.

# Table S18 Full model output for best fitting latent growth model including trajectories of behavioral difficulties and PGS of anxiety

Latent Variables:

Estimate Std.Err z-value P(>|z|) ci.lower ci.upper Std.lv Std.all

i1 =~

ytime1 1.000 1.000 1.000 1.732 0.767

ytime2 1.000 1.000 1.000 1.732 0.714

ytime3 1.000 1.000 1.000 1.732 0.761

s1 =~

ytime1 0.000 0.000 0.000 0.000 0.000

ytime2 1.500 1.500 1.500 0.792 0.326

ytime3 3.500 3.500 3.500 1.847 0.812

Regressions:

Estimate Std.Err z-value P(>|z|) ci.lower ci.upper Std.lv Std.all

ytime1 ~

sex -0.378 0.022 -16.994 0.000 -0.422 -0.334 -0.378 -0.084

by_cat -0.145 0.015 -9.622 0.000 -0.174 -0.115 -0.145 -0.047

ytime2 ~

sex -0.190 0.024 -7.938 0.000 -0.237 -0.143 -0.190 -0.039

by_cat -0.036 0.017 -2.164 0.030 -0.069 -0.003 -0.036 -0.011

ytime3 ~

sex -0.398 0.029 -13.850 0.000 -0.454 -0.342 -0.398 -0.087

by_cat -0.121 0.026 -4.606 0.000 -0.173 -0.070 -0.121 -0.039

i1 ~

adj_pgs 0.023 0.011 2.115 0.034 0.002 0.044 0.013 0.013

s1 ~

adj_pgs 0.009 0.005 2.086 0.037 0.001 0.018 0.018 0.018

Covariances:

Estimate Std.Err z-value P(>|z|) ci.lower ci.upper Std.lv Std.all

.i1 ~~

.s1 -0.336 0.019 -18.069 0.000 -0.373 -0.300 -0.368 -0.368

Intercepts:

Estimate Std.Err z-value P(>|z|) ci.lower ci.upper Std.lv Std.all

.ytime1 0.000 0.000 0.000 0.000 0.000

.ytime2 0.000 0.000 0.000 0.000 0.000

.ytime3 0.000 0.000 0.000 0.000 0.000

.i1 4.800 0.046 105.120 0.000 4.711 4.890 2.772 2.772

.s1 -0.397 0.023 -16.899 0.000 -0.443 -0.351 -0.752 -0.752

Variances:

Estimate Std.Err z-value P(>|z|) ci.lower ci.upper Std.lv Std.all

.ytime1 2.049 0.051 40.148 0.000 1.949 2.149 2.049 0.402

.ytime2 3.257 0.036 91.504 0.000 3.187 3.326 3.257 0.553

.ytime3 1.071 0.077 13.849 0.000 0.920 1.223 1.071 0.207

.i1 2.999 0.053 56.364 0.000 2.895 3.103 1.000 1.000

.s1 0.278 0.011 25.706 0.000 0.257 0.300 1.000 1.000

**Note:** std.err=standard error, std.lv=Standardized estimates on variances of the latent variables. Std.all= Standardized estimates based on variances of observed and latent variables. Ci.lower= lower 2.5% of 95% confidence interval. Ci.upper= upper 2.5% of 95% confidence interval. The models are run with sex coded (1,2) and by_cat (birth year categorized to three levels, coded as 1,2,3). Adj_pgs= polygenic score.

# Table S19 Full model output for best fitting latent growth model including trajectories of behavioral difficulties and PGS of neuroticism

Latent Variables:

Estimate Std.Err z-value P(>|z|) ci.lower ci.upper Std.lv Std.all

i1 =~

ytime1 1.000 1.000 1.000 1.732 0.767

ytime2 1.000 1.000 1.000 1.732 0.714

ytime3 1.000 1.000 1.000 1.732 0.761

s1 =~

ytime1 0.000 0.000 0.000 0.000 0.000

ytime2 1.500 1.500 1.500 0.792 0.326

ytime3 3.500 3.500 3.500 1.847 0.812

Regressions:

Estimate Std.Err z-value P(>|z|) ci.lower ci.upper Std.lv Std.all

ytime1 ~

sex -0.378 0.022 -16.992 0.000 -0.421 -0.334 -0.378 -0.084

by_cat -0.143 0.015 -9.555 0.000 -0.173 -0.114 -0.143 -0.047

ytime2 ~

sex -0.190 0.024 -7.930 0.000 -0.237 -0.143 -0.190 -0.039

by_cat -0.035 0.017 -2.101 0.036 -0.067 -0.002 -0.035 -0.011

ytime3 ~

sex -0.398 0.029 -13.850 0.000 -0.454 -0.342 -0.398 -0.087

by_cat -0.120 0.026 -4.568 0.000 -0.172 -0.069 -0.120 -0.039

i1 ~

adj_pgs 0.078 0.009 8.280 0.000 0.060 0.097 0.045 0.045

s1 ~

adj_pgs 0.000 0.000 0.000 0.000 0.000

Covariances:

Estimate Std.Err z-value P(>|z|) ci.lower ci.upper Std.lv Std.all

.i1 ~~

.s1 -0.336 0.019 -18.095 0.000 -0.373 -0.300 -0.368 -0.368

Intercepts:

Estimate Std.Err z-value P(>|z|) ci.lower ci.upper Std.lv Std.all

.ytime1 0.000 0.000 0.000 0.000 0.000

.ytime2 0.000 0.000 0.000 0.000 0.000

.ytime3 0.000 0.000 0.000 0.000 0.000

.i1 4.798 0.046 105.124 0.000 4.709 4.888 2.770 2.770

.s1 -0.397 0.023 -16.906 0.000 -0.443 -0.351 -0.752 -0.752

Variances:

Estimate Std.Err z-value P(>|z|) ci.lower ci.upper Std.lv Std.all

.ytime1 2.049 0.051 40.172 0.000 1.949 2.149 2.049 0.402

.ytime2 3.256 0.036 91.533 0.000 3.187 3.326 3.256 0.553

.ytime3 1.072 0.077 13.864 0.000 0.920 1.223 1.072 0.207

.i1 2.994 0.053 56.319 0.000 2.890 3.099 0.998 0.998

.s1 0.279 0.011 25.725 0.000 0.257 0.300 1.000 1.000

**Note:** std.err=standard error, std.lv=Standardized estimates on variances of the latent variables. Std.all= Standardized estimates based on variances of observed and latent variables. Ci.lower= lower 2.5% of 95% confidence interval. Ci.upper= upper 2.5% of 95% confidence interval. The models are run with sex coded (1,2) and by_cat (birth year categorized to three levels, coded as 1,2,3). Adj_pgs= polygenic score.

# Table S20 Full model output for best fitting latent growth model including trajectories of emotional difficulties and PGS of neuroticism

Latent Variables:

Estimate Std.Err z-value P(>|z|) ci.lower ci.upper Std.lv Std.all

i1 =~

ytime1 1.000 1.000 1.000 0.892 0.734

ytime2 1.000 1.000 1.000 0.892 0.637

ytime3 1.000 1.000 1.000 0.892 0.682

s1 =~

ytime1 0.000 0.000 0.000 0.000 0.000

ytime2 1.500 1.500 1.500 0.465 0.332

ytime3 3.500 3.500 3.500 1.084 0.829

Regressions:

Estimate Std.Err z-value P(>|z|) ci.lower ci.upper Std.lv Std.all

ytime1 ~

sex 0.018 0.012 1.492 0.136 -0.006 0.043 0.018 0.008

by_cat -0.027 0.009 -3.119 0.002 -0.044 -0.010 -0.027 -0.016

ytime2 ~

sex 0.071 0.014 5.128 0.000 0.044 0.098 0.071 0.025

by_cat 0.042 0.010 4.369 0.000 0.023 0.061 0.042 0.022

ytime3 ~

sex -0.011 0.017 -0.665 0.506 -0.044 0.022 -0.011 -0.004

by_cat 0.038 0.016 2.452 0.014 0.008 0.069 0.038 0.022

i1 ~

adj_pgs 0.049 0.005 9.324 0.000 0.038 0.059 0.055 0.055

s1 ~

adj_pgs 0.000 0.000 0.000 0.000 0.000

Covariances:

Estimate Std.Err z-value P(>|z|) ci.lower ci.upper Std.lv Std.all

.i1 ~~

.s1 -0.102 0.006 -16.535 0.000 -0.114 -0.089 -0.368 -0.368

Intercepts:

Estimate Std.Err z-value P(>|z|) ci.lower ci.upper Std.lv Std.all

.ytime1 0.000 0.000 0.000 0.000 0.000

.ytime2 0.000 0.000 0.000 0.000 0.000

.ytime3 0.000 0.000 0.000 0.000 0.000

.i1 1.345 0.026 51.615 0.000 1.294 1.396 1.508 1.508

.s1 -0.091 0.014 -6.418 0.000 -0.119 -0.063 -0.293 -0.293

Variances:

Estimate Std.Err z-value P(>|z|) ci.lower ci.upper Std.lv Std.all

.ytime1 0.680 0.017 39.976 0.000 0.647 0.714 0.680 0.461

.ytime2 1.250 0.013 97.004 0.000 1.225 1.276 1.250 0.638

.ytime3 0.448 0.028 16.165 0.000 0.393 0.502 0.448 0.262

.i1 0.793 0.017 46.000 0.000 0.759 0.827 0.997 0.997

.s1 0.096 0.004 25.943 0.000 0.089 0.103 1.000 1.000

**Note:** std.err=standard error, std.lv=Standardized estimates on variances of the latent variables. Std.all= Standardized estimates based on variances of observed and latent variables. Ci.lower= lower 2.5% of 95% confidence interval. Ci.upper= upper 2.5% of 95% confidence interval. The models are run with sex coded (1,2) and by_cat (birth year categorized to three levels, coded as 1,2,3). Adj_pgs= polygenic score.

# Table S21 Full model output for best fitting latent growth model including trajectories of emotional difficulties and PGS of bipolar disorder

Latent Variables:

Estimate Std.Err z-value P(>|z|) ci.lower ci.upper Std.lv Std.all

i1 =~

ytime1 1.000 1.000 1.000 0.892 0.734

ytime2 1.000 1.000 1.000 0.892 0.637

ytime3 1.000 1.000 1.000 0.892 0.682

s1 =~

ytime1 0.000 0.000 0.000 0.000 0.000

ytime2 1.500 1.500 1.500 0.465 0.332

ytime3 3.500 3.500 3.500 1.085 0.830

Regressions:

Estimate Std.Err z-value P(>|z|) ci.lower ci.upper Std.lv Std.all

ytime1 ~

sex 0.019 0.012 1.513 0.130 -0.006 0.043 0.019 0.008

by_cat -0.028 0.009 -3.211 0.001 -0.045 -0.011 -0.028 -0.017

ytime2 ~

sex 0.071 0.014 5.131 0.000 0.044 0.098 0.071 0.025

by_cat 0.041 0.010 4.261 0.000 0.022 0.060 0.041 0.022

ytime3 ~

sex -0.011 0.017 -0.634 0.526 -0.044 0.022 -0.011 -0.004

by_cat 0.037 0.016 2.349 0.019 0.006 0.067 0.037 0.021

i1 ~

adj_pgs 0.000 0.000 0.000 0.000 0.000

s1 ~

adj_pgs 0.000 0.000 0.000 0.000 0.000

Covariances:

Estimate Std.Err z-value P(>|z|) ci.lower ci.upper Std.lv Std.all

.i1 ~~

.s1 -0.101 0.006 -16.515 0.000 -0.114 -0.089 -0.367 -0.367

Intercepts:

Estimate Std.Err z-value P(>|z|) ci.lower ci.upper Std.lv Std.all

.ytime1 0.000 0.000 0.000 0.000 0.000

.ytime2 0.000 0.000 0.000 0.000 0.000

.ytime3 0.000 0.000 0.000 0.000 0.000

.i1 1.346 0.026 51.608 0.000 1.295 1.397 1.509 1.509

.s1 -0.090 0.014 -6.389 0.000 -0.118 -0.063 -0.292 -0.292

Variances:

Estimate Std.Err z-value P(>|z|) ci.lower ci.upper Std.lv Std.all

.ytime1 0.680 0.017 39.927 0.000 0.647 0.713 0.680 0.461

.ytime2 1.251 0.013 96.965 0.000 1.225 1.276 1.251 0.638

.ytime3 0.447 0.028 16.121 0.000 0.392 0.501 0.447 0.261

.i1 0.795 0.017 46.079 0.000 0.762 0.829 1.000 1.000

.s1 0.096 0.004 25.955 0.000 0.089 0.103 1.000 1.000

**Note:** std.err=standard error, std.lv=Standardized estimates on variances of the latent variables. Std.all= Standardized estimates based on variances of observed and latent variables. Ci.lower= lower 2.5% of 95% confidence interval. Ci.upper= upper 2.5% of 95% confidence interval. The models are run with sex coded (1,2) and by_cat (birth year categorized to three levels, coded as 1,2,3). Adj_pgs= polygenic score.

# Table S22 Full model output for best fitting latent growth model including trajectories of behavioral difficulties and PGS of bipolar disorder

Latent Variables:

Estimate Std.Err z-value P(>|z|) ci.lower ci.upper Std.lv Std.all

i1 =~

ytime1 1.000 1.000 1.000 1.732 0.767

ytime2 1.000 1.000 1.000 1.732 0.714

ytime3 1.000 1.000 1.000 1.732 0.761

s1 =~

ytime1 0.000 0.000 0.000 0.000 0.000

ytime2 1.500 1.500 1.500 0.792 0.326

ytime3 3.500 3.500 3.500 1.847 0.812

Regressions:

Estimate Std.Err z-value P(>|z|) ci.lower ci.upper Std.lv Std.all

ytime1 ~

sex -0.378 0.022 -16.982 0.000 -0.421 -0.334 -0.378 -0.084

by_cat -0.145 0.015 -9.635 0.000 -0.174 -0.115 -0.145 -0.048

ytime2 ~

sex -0.190 0.024 -7.929 0.000 -0.237 -0.143 -0.190 -0.039

by_cat -0.036 0.017 -2.196 0.028 -0.069 -0.004 -0.036 -0.011

ytime3 ~

sex -0.397 0.029 -13.818 0.000 -0.454 -0.341 -0.397 -0.087

by_cat -0.123 0.026 -4.658 0.000 -0.174 -0.071 -0.123 -0.040

i1 ~

adj_pgs 0.000 0.000 0.000 0.000 0.000

s1 ~

adj_pgs 0.000 0.000 0.000 0.000 0.000

Covariances:

Estimate Std.Err z-value P(>|z|) ci.lower ci.upper Std.lv Std.all

.i1 ~~

.s1 -0.336 0.019 -18.053 0.000 -0.372 -0.299 -0.367 -0.367

Intercepts:

Estimate Std.Err z-value P(>|z|) ci.lower ci.upper Std.lv Std.all

.ytime1 0.000 0.000 0.000 0.000 0.000

.ytime2 0.000 0.000 0.000 0.000 0.000

.ytime3 0.000 0.000 0.000 0.000 0.000

.i1 4.800 0.046 105.110 0.000 4.710 4.890 2.772 2.772

.s1 -0.397 0.023 -16.883 0.000 -0.443 -0.350 -0.751 -0.751

Variances:

Estimate Std.Err z-value P(>|z|) ci.lower ci.upper Std.lv Std.all

.ytime1 2.049 0.051 40.147 0.000 1.949 2.149 2.049 0.402

.ytime2 3.256 0.036 91.492 0.000 3.187 3.326 3.256 0.553

.ytime3 1.072 0.077 13.854 0.000 0.920 1.223 1.072 0.207

.i1 2.999 0.053 56.370 0.000 2.895 3.104 1.000 1.000

.s1 0.278 0.011 25.705 0.000 0.257 0.300 1.000 1.000

**Note:** std.err=standard error, std.lv=Standardized estimates on variances of the latent variables. Std.all= Standardized estimates based on variances of observed and latent variables. Ci.lower= lower 2.5% of 95% confidence interval. Ci.upper= upper 2.5% of 95% confidence interval. The models are run with sex coded (1,2) and by_cat (birth year categorized to three levels, coded as 1,2,3). Adj_pgs= polygenic score.

# Table S23 Evaluating best fitting latent growth model for emotional and behavioral difficulties for PGS of depression with covariate effects constrained to equal

| Childhood difficulty | Model | Df | AIC | Chisq.diff | P(>Chisq) |
| --- | --- | --- | --- | --- | --- |
| **Emotional difficulties** |  |  |  |  |  |
|  | Age specific PGS effects | 5 | 290843.4 |  |  |
|  | PGS effect on growth factors | 6 | 290842.9 | 1.481 | 0.224 |
|  | PGS effect on growth factors | 6 | 290842.9 |  |  |
|  | **PGS effect on intercept only** | **7** | **290841.5** | **0.667** | **0.414** |
|  | PGS effect on growth factors | 6 | 290842.9 |  |  |
|  | PGS effect on slope only | 7 | 290855.0 | 14.125 | 0.000 |
|  | PGS effect on intercept only | 7 | 290841.5 |  |  |
|  | No PGS effect | 8 | 290863.2 | 23.638 | 0.000 |
| **Behavioral difficulties** |  |  |  |  |  |
|  | Age specific PGS effects | 5 | 400810.8 |  |  |
|  | **PGS effect on growth factors** | **6** | **400808.8** | **0.045** | **0.833** |
|  | PGS effect on growth factors | 6 | 400808.8 |  |  |
|  | PGS effect on intercept only | 7 | 400830.3 | 23.480 | 0.000 |
|  | PGS effect on growth factors | 6 | 400808.8 |  |  |
|  | PGS effect on slope only | 7 | 400817.1 | 10.335 | 0.001 |
|  | PGS effect on growth factors | 6 | 400808.8 |  |  |
|  | No PGS effect | 8 | 400870.2 | 65.410 | 0.000 |

**Note** Df= Degrees of Freedom, AIC= First-order Akaike Information Criteria, Chisq.diff= Difference in Chi Square Value. P(>Chisq) = P-value for Chi Square Test. The models are compared in a step-wise-manner, thus in this table each model is compared to the model listed immediately above. The final selected model is highlighted in bold.

# Table S24 Evaluating best fitting latent growth model for emotional and behavioral difficulties for PGS of anxiety with covariate effects constrained to equal

| Childhood difficulty | Model | Df | AIC | Chisq.diff | P(>Chisq) |
| --- | --- | --- | --- | --- | --- |
| **Emotional difficulties** |  |  |  |  |  |
|  | Age specific PGS effects | 5 | 290850.7 |  |  |
|  | **PGS effect on growth factors** | **6** | **290849.0** | **0.309** | **0.578** |
|  | PGS effect on growth factors | 6 | 290849.0 |  |  |
|  | PGS effect on intercept only | 7 | 290851.1 | 4.100 | 0.043 |
|  | PGS effect on growth factors | 6 | 290849.0 |  |  |
|  | PGS effect on slope only | 7 | 290851.8 | 4.790 | 0.029 |
|  | PGS effect on growth factors | 6 | 290849.0 |  |  |
|  | No PGS effect | 8 | 290863.2 | 18.182 | 0.000 |
| **Behavioral difficulties** |  |  |  |  |  |
|  | Age specific PGS effects | 5 | 400857.6 |  |  |
|  | **PGS effect on growth factors** | **6** | **400856.7** | **1.167** | **0.280** |
|  | PGS effect on growth factors | 6 | 400856.7 |  |  |
|  | PGS effect on intercept only | 7 | 400859.5 | 4.737 | 0.030 |
|  | PGS effect on growth factors | 6 | 400856.7 |  |  |
|  | PGS effect on slope only | 7 | 400858.8 | 4.099 | 0.043 |
|  | PGS effect on growth factors | 6 | 400856.7 |  |  |
|  | No PGS effect | 8 | 400870.2 | 17.493 | 0.000 |

**Note:** Df= Degrees of Freedom, AIC= First-order Akaike Information Criteria, Chisq.diff= Difference in Chi Square Value. P(>Chisq) = P-value for Chi Square Test. The models are compared in a step-wise-manner, thus in this table each model is compared to the model listed immediately above. The final selected model is highlighted in bold.

# Table S25 Evaluating best fitting latent growth model for emotional and behavioral difficulties and PGS of neuroticism with covariate effects constrained to equal

| Childhood difficulty | Model | Df | AIC | Chisq.diff | P(>Chisq) |
| --- | --- | --- | --- | --- | --- |
| **Emotional difficulties** |  |  |  |  |  |
|  | Age specific PGS effects | 5 | 290781.5 |  |  |
|  | PGS effect on growth factors | 6 | 290779.5 | 0.008 | 0.930 |
|  | PGS effect on growth factors | 6 | 290779.5 |  |  |
|  | **PGS effect on intercept only** | **7** | **290779.4** | **1.863** | **0.172** |
|  | PGS effect on growth factors | 6 | 290779.5 |  |  |
|  | PGS effect on slope only | 7 | 290830.3 | 52.810 | 0.000 |
|  | PGS effect on intercept only | 7 | 290779.4 |  |  |
|  | No PGS effect | 8 | 290863.2 | 85.781 | 0.000 |
| **Behavioral difficulties** |  |  |  |  |  |
|  | Age specific PGS effects | 5 | 400806.2 |  |  |
|  | PGS effect on growth factors | 6 | 400804.6 | 0.412 | 0.521 |
|  | PGS effect on growth factors | 6 | 400804.6 |  |  |
|  | **PGS effect on intercept only** | **7** | **400805.0** | **2.316** | **0.128** |
|  | PGS effect on growth factors | 6 | 400804.6 |  |  |
|  | PGS effect on slope only | 7 | 400843.3 | 40.651 | 0.000 |
|  | PGS effect on intercept only | 7 | 400805.0 |  |  |
|  | No PGS effect | 8 | 400870.2 | 67.259 | 0.000 |

**Note:** Df= Degrees of Freedom, AIC= First-order Akaike Information Criteria, Chisq.diff= Difference in Chi Square Value. P(>Chisq) = P-value for Chi Square Test. The models are compared in a step-wise-manner, thus in this table each model is compared to the model listed immediately above. The final selected model is highlighted in bold.

# Table S26 Evaluating best fitting latent growth model for emotional and behavioral difficulties for PGS of bipolar disorder with covariate effects constrained to equal

| Childhood difficulty | Model | Df | AIC | Chisq.diff | P(>Chisq) |
| --- | --- | --- | --- | --- | --- |
| **Emotional difficulties** |  |  |  |  |  |
|  | Age specific PGS effects | 5 | 290867.2 |  |  |
|  | PGS effect on growth factors | 6 | 290865.2 | 0.023 | 0.880 |
|  | PGS effect on growth factors | 6 | 290865.2 |  |  |
|  | PGS effect on intercept only | 7 | 290863.3 | 0.046 | 0.830 |
|  | PGS effect on growth factors | 6 | 290865.2 |  |  |
|  | PGS effect on slope only | 7 | 290864.4 | 1.140 | 0.286 |
|  | PGS effect on intercept only | 7 | 290863.3 |  |  |
|  | No PGS effect | 8 | 290863.2 | 1.876 | 0.171 |
|  | PGS effect on slope only | 7 | 290864.4 |  |  |
|  | **No PGS effect** | **8** | **290863.2** | **0.782** | **0.377** |
| **Behavioral difficulties** |  |  |  |  |  |
|  | Age specific PGS effects | 5 | 400870.5 |  |  |
|  | PGS effect on growth factors | 6 | 400869.7 | 1.182 | 0.277 |
|  | PGS effect on growth factors | 6 | 400869.7 |  |  |
|  | PGS effect on intercept only | 7 | 400869.9 | 2.261 | 0.133 |
|  | PGS effect on growth factors | 6 | 400869.7 |  |  |
|  | PGS effect on slope only | 7 | 400871.9 | 4.236 | 0.040 |
|  | PGS effect on intercept only | 7 | 400869.9 |  |  |
|  | **No PGS effect** | **8** | **400870.2** | **2.284** | **0.131** |

**Note:** Df= Degrees of Freedom, AIC= First-order Akaike Information Criteria, Chisq.diff= Difference in Chi Square Value. P(>Chisq) = P-value for Chi Square Test. The models are compared in a step-wise-manner, thus in this table each model is compared to the model listed immediately above. The final selected model is highlighted in bold.

# Table S27: Model fit statistics for latent profile analyses

| **Model** | **LL** | **AIC** | **AICc** | **Entropy** | **VLMR 2LL Diff** | **VLMR test *p* value** |
| --- | --- | --- | --- | --- | --- | --- |
| 1 profile | -639955.21 | 1280004.41 | 1280004.53 |  |  |  |
| 2 profiles | -632923.412 | 1265962.82 | 1265962.98 | 0.815 | 14063.59 | <0.001 |
| 3 profiles | -629359.153 | 1258856.31 | 1258856.53 | 0.786 | 7128.52 | <0.001 |
| 4 profiles | -625839.72 | 1251839.44 | 1251839.74 | 0.761 | 6283.80 | <0.001 |
| 5 profiles | -623605.72 | 1247393.44 | 1247393.82 | 0.759 | 4468.00 | 0.0024 |
| 6 profiles | -621977.53 | 1244159.05 | 1244159.54 | 0.762 | 2230.48 | 0.3189 |
| 7 profiles | -620662.17 | 1241550.35 | 1241550.94 | 0.749 | 1231.34 | 0.1393 |
| 8 profiles | -618652.72 | 1237553.43 | 1237554.15 | 0.759 | 2639.33 | 0.1864 |

**Note:** LL= Loglikelihood value for final model. AIC= First-order Akaike Information Criterion. AICc= Second-order Akaike Information Criterion. VLMR= Vuong–Lo–Mendell–Rubin likelihood ratio test.

# Table S28: Distribution of individuals in each profile with five-profile-model

| **profile** | **count** | **proportion** | **prob_Class**  **1** | **prob_Clas**  **2** | **prob_Class3** | **prob_Class4** | **prob_Class5** |
| --- | --- | --- | --- | --- | --- | --- | --- |
| 1 | 2099 | 0.04815 | 0.784 | 0.141 | 0.023 | 0.039 | 0.013 |
| 2 | 37071 | 0.85041 | 0.028 | 0.878 | 0.057 | 0.028 | 0.01 |
| 3 | 2113 | 0.04847 | 0.005 | 0.06 | 0.912 | 0.016 | 0.007 |
| 4 | 1828 | 0.04193 | 0.027 | 0.114 | 0.078 | 0.703 | 0.077 |
| 5 | 481 | 0.01103 | 0 | 0 | 0.03 | 0.004 | 0.966 |

Table S29: Relative odds of any emotional disorder given assignment to specific developmental profile.

| **Comparison** | **Odds ratio (OR)** | **95% CI** | |
| --- | --- | --- | --- |
|  |  | **Lower 2.5%** | **Upper 2.5%** |
| Profile 1 vs. Reference | 2.800 | 2.351 | 3.249 |
| Profile 3 vs. Reference | 1.534 | 1.248 | 1.820 |
| Profile 4 vs. Reference | 1.804 | 1.390 | 2.218 |
| Profile 5 vs. Reference | 2.937 | 2.128 | 3.746 |
| Profile 3 vs. Profile 1 | 0.548 | 0.425 | 0.671 |
| Profile 4 vs. Profile 1 | 0.644 | 0.470 | 0.818 |
| Profile 5 vs. Profile 1 | 1.049 | 0.727 | 1.371 |
| Profile 4 vs. Profile 3 | 1.176 | 0.841 | 1.511 |
| Profile 5 vs. Profile 3 | 1.914 | 1.313 | 2.516 |
| Profile 5 vs. Profile 4 | 1.628 | 1.020 | 2.235 |

**Note:** All profiles were compared to each other.

# Table S30: Relative odds of assignment to specific developmental profile per standard deviation increase in polygenic score for depression.

| **Comparison** | **Odds ratio (OR)** | **95% CI** | |
| --- | --- | --- | --- |
|  |  | **Lower 2.5%** | **Upper 2.5%** |
| Profile 1 vs. Reference | 1.130 | 1.063 | 1.200 |
| Profile 3 vs. Reference | 1.112 | 1.053 | 1.174 |
| Profile 4 vs. Reference | 1.153 | 1.067 | 1.246 |
| Profile 5 vs. Reference | 1.217 | 1.096 | 1.351 |
| Profile 3 vs. Profile 1 | 0.984 | 0.912 | 1.062 |
| Profile 4 vs. Profile 1 | 1.021 | 0.925 | 1.126 |
| Profile 5 vs. Profile 1 | 1.077 | 0.957 | 1.213 |
| Profile 4 vs. Profile 3 | 1.037 | 0.944 | 1.139 |
| Profile 5 vs. Profile 3 | 1.094 | 0.976 | 1.227 |
| Profile 5 vs. Profile 4 | 1.056 | 0.918 | 1.214 |

**Note**: All profiles were compared to each other.

# Table S31: Relative odds of assignment to specific developmental profile per standard deviation increase in polygenic score for anxiety.

| **Comparison** | **Odds ratio (OR)** | **95% CI** | |
| --- | --- | --- | --- |
|  |  | **Lower 2.5%** | **Upper 2.5%** |
| Profile 1 vs. Reference | 1.109 | 1.047 | 1.175 |
| Profile 3 vs. Reference | 1.064 | 1.008 | 1.122 |
| Profile 4 vs. Reference | 1.048 | 0.974 | 1.128 |
| Profile 5 vs. Reference | 1.089 | 0.985 | 1.204 |
| Profile 3 vs. Profile 1 | 0.959 | 0.891 | 1.032 |
| Profile 4 vs. Profile 1 | 0.945 | 0.861 | 1.036 |
| Profile 5 vs. Profile 1 | 0.982 | 0.876 | 1.100 |
| Profile 4 vs. Profile 3 | 0.985 | 0.901 | 1.077 |
| Profile 5 vs. Profile 3 | 1.024 | 0.916 | 1.143 |
| Profile 5 vs. Profile 4 | 1.039 | 0.911 | 1.185 |

**Note:** All profiles were compared to each other.

# Table S32: Relative odds of assignment to specific developmental profile per standard deviation increase in polygenic score for bipolar disorder.

| **Comparison** | **Odds ratio (OR)** | **95% CI** | |
| --- | --- | --- | --- |
|  |  | **Lower 2.5%** | **Upper 2.5%** |
| Profile 1 vs. Reference | 1.011 | 0.954 | 1.072 |
| Profile 3 vs. Reference | 1.039 | 0.985 | 1.096 |
| Profile 4 vs. Reference | 1.001 | 0.929 | 1.079 |
| Profile 5 vs. Reference | 1.198 | 1.082 | 1.327 |
| Profile 3 vs. Profile 1 | 1.027 | 0.954 | 1.107 |
| Profile 4 vs. Profile 1 | 0.990 | 0.901 | 1.088 |
| Profile 5 vs. Profile 1 | 1.185 | 1.056 | 1.330 |
| Profile 4 vs. Profile 3 | 0.964 | 0.880 | 1.055 |
| Profile 5 vs. Profile 3 | 1.154 | 1.031 | 1.290 |
| Profile 5 vs. Profile 4 | 1.197 | 1.047 | 1.369 |

**Note**: All profiles were compared to each other.

# Table S33: Relative odds of assignment to specific developmental profile per standard deviation increase in polygenic score for neuroticism.

| **Comparison** | **Odds ratio (OR)** | **95% CI** | |
| --- | --- | --- | --- |
|  |  | **Lower 2.5%** | **Upper 2.5%** |
| Profile 1 vs. Reference | 1.209 | 1.139 | 1.283 |
| Profile 3 vs. Reference | 1.060 | 1.004 | 1.118 |
| Profile 4 vs. Reference | 1.157 | 1.075 | 1.245 |
| Profile 5 vs. Reference | 1.056 | 0.952 | 1.170 |
| Profile 3 vs. Profile 1 | 0.877 | 0.813 | 0.945 |
| Profile 4 vs. Profile 1 | 0.957 | 0.871 | 1.051 |
| Profile 5 vs. Profile 1 | 0.873 | 0.777 | 0.982 |
| Profile 4 vs. Profile 3 | 1.091 | 0.998 | 1.193 |
| Profile 5 vs. Profile 3 | 0.996 | 0.890 | 1.115 |
| Profile 5 vs. Profile 4 | 0.913 | 0.799 | 1.043 |

**Note:** All profiles were compared to each other.

# **Table S34: Depression PGS. Linear association between standardized PGS score and standardized score of emotional and behavioral difficulties** including parental education as covariate.

| **Characteristic** | $\boldsymbol{\beta}$ | **95% CI Lower 2.5%** | **95% CI**  **Upper 2.5%** | ***p*-value** | ***p-FDR*^2^** |
| --- | --- | --- | --- | --- | --- |
| Emotional difficulties 1.5 yrs | 0.01 | 0.00 | 0.03 | 0.004 | 0.006 |
| Emotional difficulties 3yrs | 0.01 | 0.00 | 0.02 | 0.026 | 0.034 |
| Emotional difficulties 5 yrs | 0.02 | 0.01 | 0.04 | 0.001 | 0.001 |
| Behavioral difficulties 1.5yrs | 0.01 | 0.00 | 0.02 | 0.053 | 0.067 |
| Behavioral difficulties 3yrs | 0.03 | 0.02 | 0.04 | <0.001 | <0.001 |
| Behavioral difficulties 5 yrs | 0.04 | 0.03 | 0.06 | <0.001 | <0.001 |
| Depressive symptoms 8yrs | 0.05 | 0.04 | 0.07 | <0.001 | <0.001 |
| Anxiety symptoms 8yrs | 0.03 | 0.02 | 0.04 | <0.001 | <0.001 |
| Inattention 8yrs | 0.03 | 0.02 | 0.04 | <0.001 | <0.001 |
| Oppositional defiant disorder symptoms 8yrs | 0.05 | 0.03 | 0.06 | <0.001 | <0.001 |
| Hyperactivity 8yrs | 0.03 | 0.02 | 0.04 | <0.001 | <0.001 |
| Conduct disorder symptoms 8yrs | 0.04 | 0.03 | 0.05 | <0.001 | <0.001 |

# **Table S35: Neuroticism PGS. Linear association between standardized PGS score and standardized score of emotional and behavioral difficulties** including parental education as covariate.

| **Characteristic** | $\boldsymbol{\beta}$ | **95% CI Lower 2.5%** | **95% CI**  **Upper 2.5%** | ***p*-value** | ***p-FDR*^2^** |
| --- | --- | --- | --- | --- | --- |
| Emotional difficulties 1.5 yrs | 0.03 | 0.02 | 0.04 | <0.001 | <0.001 |
| Emotional difficulties 3yrs | 0.03 | 0.02 | 0.04 | <0.001 | <0.001 |
| Emotional difficulties 5 yrs | 0.04 | 0.03 | 0.05 | <0.001 | <0.001 |
| Behavioral difficulties 1.5yrs | 0.03 | 0.02 | 0.04 | <0.001 | <0.001 |
| Behavioral difficulties 3yrs | 0.03 | 0.02 | 0.04 | <0.001 | <0.001 |
| Behavioral difficulties 5 yrs | 0.04 | 0.02 | 0.05 | <0.001 | <0.001 |
| Depressive symptoms 8yrs | 0.06 | 0.05 | 0.08 | <0.001 | <0.001 |
| Anxiety symptoms 8yrs | 0.05 | 0.04 | 0.06 | <0.001 | <0.001 |
| Inattention 8yrs | 0.03 | 0.01 | 0.04 | <0.001 | <0.001 |
| Oppositional defiant disorder symptoms 8yrs | 0.04 | 0.03 | 0.05 | <0.001 | <0.001 |
| Hyperactivity 8yrs | 0.02 | 0.01 | 0.03 | 0.002 | 0.004 |
| Conduct disorder symptoms 8yrs | 0.02 | 0.00 | 0.03 | 0.015 | 0.021 |

# Table S36: Anxiety disorder PGS. Linear association between standardized PGS score and standardized score of emotional and behavioral difficulties including parental education as covariate.

| **Characteristic** | $\boldsymbol{\beta}$ | **95% CI Lower 2.5%** | **95% CI**  **Upper 2.5%** | ***p*-value** | ***p-FDR*^2^** |
| --- | --- | --- | --- | --- | --- |
| Emotional difficulties 1.5 yrs | 0.01 | 0.00 | 0.02 | 0.131 | 0.161 |
| Emotional difficulties 3yrs | 0.01 | 0.00 | 0.03 | 0.011 | 0.016 |
| Emotional difficulties 5 yrs | 0.03 | 0.01 | 0.04 | <0.001 | <0.001 |
| Behavioral difficulties 1.5yrs | 0.01 | 0.00 | 0.02 | 0.234 | 0.274 |
| Behavioral difficulties 3yrs | 0.01 | 0.00 | 0.02 | 0.020 | 0.027 |
| Behavioral difficulties 5 yrs | 0.02 | 0.01 | 0.03 | 0.002 | 0.003 |
| Depressive symptoms 8yrs | 0.04 | 0.03 | 0.05 | <0.001 | <0.001 |
| Anxiety symptoms 8yrs | 0.03 | 0.01 | 0.04 | <0.001 | <0.001 |
| Inattention 8yrs | 0.02 | 0.01 | 0.03 | 0.002 | 0.004 |
| Oppositional defiant disorder symptoms 8yrs | 0.03 | 0.01 | 0.04 | 0.000 | 0.000 |
| Hyperactivity 8yrs | 0.02 | 0.01 | 0.03 | 0.003 | 0.004 |
| Conduct disorder symptoms 8yrs | 0.02 | 0.01 | 0.03 | 0.006 | 0.008 |

# **Table S37: Bipolar disorder PGS. Linear association between standardized PGS score and standardized score of emotional and behavioral difficulties** including parental education as covariate

.

| **Characteristic** | $\boldsymbol{\beta}$ | **95% CI Lower 2.5%** | **95% CI**  **Upper 2.5%** | ***p*-value** | ***p-FDR*^2^** |
| --- | --- | --- | --- | --- | --- |
| Emotional difficulties 1.5 yrs | 0.00 | -0.01 | 0.01 | 0.474 | 0.506 |
| Emotional difficulties 3yrs | 0.00 | -0.01 | 0.01 | 0.593 | 0.606 |
| Emotional difficulties 5 yrs | -0.01 | -0.02 | 0.01 | 0.291 | 0.332 |
| Behavioral difficulties 1.5yrs | -0.01 | -0.02 | 0.00 | 0.219 | 0.263 |
| Behavioral difficulties 3yrs | 0.00 | -0.01 | 0.01 | 0.551 | 0.575 |
| Behavioral difficulties 5 yrs | 0.01 | -0.01 | 0.02 | 0.372 | 0.406 |
| Depressive symptoms 8yrs | 0.02 | 0.01 | 0.04 | 0.001 | 0.001 |
| Anxiety symptoms 8yrs | 0.00 | -0.01 | 0.02 | 0.705 | 0.705 |
| Inattention 8yrs | 0.01 | -0.01 | 0.02 | 0.359 | 0.400 |
| Oppositional defiant disorder symptoms 8yrs | 0.03 | 0.02 | 0.04 | <0.001 | <0.001 |
| Hyperactivity 8yrs | 0.02 | 0.01 | 0.03 | 0.001 | 0.001 |
| Conduct disorder symptoms 8yrs | 0.03 | 0.02 | 0.04 | <0.001 | <0.001 |

# Table S38 Model fit for basic linear latent growth models across early childhood including parental education as covariate

| **Measurement** | **CFI** | **TLI** | **RMSEA (95% CI)** | **SRMR** | **Chisq** | ***p*** | **DF** |
| --- | --- | --- | --- | --- | --- | --- | --- |
| Emotional difficulties | 0.996 | 0.958 | 0.024 (0.017, 0.031) | 0.007 | 31.45 | <0.001 | 1 |
| Behavioral difficulties | 0.995 | 0.937 | 0.038 (0.029, 0.043) | 0.009 | 71.781 | <0.001 | 1 |

**Note:** CFI= Comparative fit index; TLI=Tucker Lewis Index and RMSEA=Root Mean Square Error of Approximation. SRMR=Standardized Root Mean Square Residual. Chisq= chi-square (χ2) statistic. DF= Degrees of freedom.

# Table S39 Evaluating best fitting latent growth model for emotional and behavioral difficulties for PGS of depression including parental education as covariate

| Childhood difficulty | Model | Df | AIC | Chisq.diff | P(>Chisq) |
| --- | --- | --- | --- | --- | --- |
| **Emotional difficulties** |  |  |  |  |  |
|  | Age specific PGS effects | 1 | 282581.5 |  |  |
|  | PGS effect on growth factors | 2 | 282579.7 | 0.118 | 0.731 |
|  | PGS effect on growth factors | 2 | 282579.7 |  |  |
|  | **PGS effect on intercept only** | **3** | **282578.7** | **1.073** | **0.300** |
|  | PGS effect on growth factors | 2 | 282579.7 |  |  |
|  | PGS effect on slope only | 3 | 282585.7 | 8.088 | 0.004 |
|  | PGS effect on intercept only | 3 | 282578.7 |  |  |
|  | No PGS effect | 4 | 282592.2 | 15.468 | 0.000 |
| **Behavioral difficulties** |  |  |  |  |  |
|  | Age specific PGS effects | 1 | 391316.4 |  |  |
|  | **PGS effect on growth factors** | **2** | **391315.9** | **1.426** | **0.232** |
|  | PGS effect on growth factors | 2 | 391315.9 |  |  |
|  | PGS effect on intercept only | 3 | 391335.4 | 21.572 | 0.000 |
|  | PGS effect on growth factors | 2 | 391315.9 |  |  |
|  | PGS effect on slope only | 3 | 391319.5 | 5.636 | 0.018 |
|  | PGS effect on growth factors | 2 | 391315.9 |  |  |
|  | No PGS effect | 4 | 391363.0 | 51.159 | 0.000 |

**Note:** Df= Degrees of Freedom, AIC= First-order Akaike Information Criteria, Chisq.diff= Difference in Chi Square Value. P(>Chisq) = P-value for Chi Square Test. The models are compared in a step-wise-manner, thus in this table each model is compared to the model listed immediately above. The final selected model is highlighted in bold.

# Table S40 Evaluating best fitting latent growth model for emotional and behavioral difficulties for PGS of anxiety including parental education as covariate

| Childhood difficulty | Model | Df | AIC | Chisq.diff | P(>Chisq) |
| --- | --- | --- | --- | --- | --- |
| **Emotional difficulties** |  |  |  |  |  |
|  | Age specific PGS effects | 1 | 282576.7 |  |  |
|  | PGS effect on growth factors | 2 | 282574.7 | 0.000 | 0.990 |
|  | PGS effect on growth factors | 2 | 282574.7 |  |  |
|  | PGS effect on intercept only | 3 | 282577.5 | 4.848 | 0.028 |
|  | PGS effect on growth factors | 2 | 282574.7 |  |  |
|  | **PGS effect on slope only** | **3** | **282574.9** | **2.241** | **0.134** |
|  | PGS effect on slope only | 3 | 282574.9 |  |  |
|  | No PGS effect | 4 | 282584.9 | 11.964 | 0.001 |
| **Behavioral difficulties** |  |  |  |  |  |
|  | Age specific PGS effects | 1 | 391349.8 |  |  |
|  | PGS effect on growth factors | 2 | 391347.9 | 0.046 | 0.829 |
|  | PGS effect on growth factors | 2 | 391347.9 |  |  |
|  | PGS effect on intercept only | 3 | 391350.0 | 4.083 | 0.043 |
|  | PGS effect on growth factors | 2 | 391347.9 |  |  |
|  | **PGS effect on slope only** | **3** | **391347.8** | **1.936** | **0.164** |
|  | PGS effect on slope only | 3 | 391347.8 |  |  |
|  | No PGS effect | 4 | 391355.7 | 9.855 | 0.002 |

**Note:** Df= Degrees of Freedom, AIC= First-order Akaike Information Criteria, Chisq.diff= Difference in Chi Square Value. P(>Chisq) = P-value for Chi Square Test. The models are compared in a step-wise-manner, thus in this table each model is compared to the model listed immediately above. The final selected model is highlighted in bold.

# Table S41 Evaluating best fitting latent growth model for emotional and behavioral difficulties and PGS of neuroticism including parental education as covariate

| Childhood difficulty | Model | Df | AIC | Chisq.diff | P(>Chisq) |
| --- | --- | --- | --- | --- | --- |
| **Emotional difficulties** |  |  |  |  |  |
|  | Age specific PGS effects | 1 | 282522.6 |  |  |
|  | PGS effect on growth factors | 2 | 282521.2 | 0.558 | 0.455 |
|  | PGS effect on growth factors | 2 | 282521.2 |  |  |
|  | **PGS effect on intercept only** | **3** | **282522.0** | **2.753** | **0.097** |
|  | PGS effect on growth factors | 2 | 282521.2 |  |  |
|  | PGS effect on slope only | 3 | 282559.4 | 40.213 | 0.000 |
|  | PGS effect on intercept only | 3 | 282522.0 |  |  |
|  | No PGS effect | 4 | 282590.0 | 70.058 | 0.000 |
| **Behavioral difficulties** |  |  |  |  |  |
|  | Age specific PGS effects | 1 | 391312.4 |  |  |
|  | PGS effect on growth factors | 2 | 391310.6 | 0.125 | 0.724 |
|  | PGS effect on growth factors | 2 | 391310.6 |  |  |
|  | **PGS effect on intercept only** | **3** | **391310.7** | **2.093** | **0.148** |
|  | PGS effect on growth factors | 2 | 391310.6 |  |  |
|  | PGS effect on slope only | 3 | 391339.0 | 30.385 | 0.000 |
|  | PGS effect on intercept only | 3 | 391310.7 |  |  |
|  | No PGS effect | 4 | 391360.5 | 51.836 | 0.000 |

**Note:** Df= Degrees of Freedom, AIC= First-order Akaike Information Criteria, Chisq.diff= Difference in Chi Square Value. P(>Chisq) = P-value for Chi Square Test. The models are compared in a step-wise-manner, thus in this table each model is compared to the model listed immediately above. The final selected model is highlighted in bold.

# Table S42 Evaluating best fitting latent growth model for emotional and behavioral difficulties for PGS of bipolar disorder including parental education as covariate

| Childhood difficulty | Model | Df | AIC | Chisq.diff | P(>Chisq) |
| --- | --- | --- | --- | --- | --- |
| **Emotional difficulties** |  |  |  |  |  |
|  | Age specific PGS effects | 1 | 282584.1 |  |  |
|  | PGS effect on growth factors | 2 | 282582.1 | 0.009 | 0.926 |
|  | PGS effect on growth factors | 2 | 282582.1 |  |  |
|  | PGS effect on intercept only | 3 | 282580.3 | 0.142 | 0.706 |
|  | PGS effect on growth factors | 2 | 282582.1 |  |  |
|  | PGS effect on slope only | 3 | 282580.4 | 0.308 | 0.579 |
|  | PGS effect on intercept only | 3 | 282580.3 |  |  |
|  | No PGS effect | 4 | 282579.0 | 0.758 | 0.384 |
|  | PGS effect on slope only | 3 | 282580.4 |  |  |
|  | **No PGS effect** | **4** | **282579.0** | **0.593** | **0.441** |
| **Behavioral difficulties** |  |  |  |  |  |
|  | Age specific PGS effects | 1 | 391351.9 |  |  |
|  | PGS effect on growth factors | 2 | 391350.3 | 0.416 | 0.519 |
|  | PGS effect on growth factors | 2 | 391350.3 |  |  |
|  | PGS effect on intercept only | 3 | 391350.5 | 2.198 | 0.138 |
|  | PGS effect on growth factors | 2 | 391350.3 |  |  |
|  | PGS effect on slope only | 3 | 391350.7 | 2.419 | 0.120 |
|  | PGS effect on intercept only | 3 | 391350.5 |  |  |
|  | No PGS effect | 4 | 391349.4 | 0.881 | 0.348 |
|  | PGS effect on slope only | 3 | 391350.7 |  |  |
|  | **No PGS effect** | **4** | **391349.4** | **0.660** | **0.416** |

**Note:** Df= Degrees of Freedom, AIC= First-order Akaike Information Criteria, Chisq.diff= Difference in Chi Square Value. P(>Chisq) = P-value for Chi Square Test. The models are compared in a step-wise-manner, thus in this table each model is compared to the model listed immediately above. The final selected model is highlighted in bold.

# Table S43 Standardized beta for PGS on best performing latent growth model for emotional and behavioral difficulties across early childhood including parental education as covariate

| **Model** | $\boldsymbol{\beta}$ | **95% CI** | ***p*-value** | ***p*-FDR** |
| --- | --- | --- | --- | --- |
| PGS_DEP_ effect on intercept emotional difficulties | 0.023 | (0.012, 0.035) | <0.001 | <0.001 |
| PGS_DEP_ effect on intercept behavioral difficulties | 0.015 | (0.003, 0.028) | 0.018 | 0.018 |
| PGS_DEP_ effect on slope behavioral difficulties | 0.040 | (0.023, 0.057) | <0.001 | <0.001 |
|  |  |  |  |  |
| PGS_NEUR_ effect on intercept emotional difficulties | 0.049 | (0.038, 0.061) | <0.001 | <0.001 |
| PGS_NEUR_ effect on intercept behavioral difficulties | 0.039 | (0.029, 0.050) | <0.001 | <0.001 |
|  |  |  |  |  |
| No PGS_BD_ effect on emotional difficulties | N/A | N/A | N/A | N/A |
| No PGS_BD_ effect on behavioral difficulties | N/A | N/A | N/A | N/A |
|  |  |  |  |  |
| PGS_ANX_ effect on slope emotional difficulties | 0.026 | (0.011, 0.041) | 0.001 | 0.001 |
| PGS_ANX_ effect on slope behavioral difficulties | 0.023 | (0.009, 0.038) | 0.002 | 0.002 |

**Note:** ANX = anxiety, DEP = depression, BD = bipolar disorder, NEUR = neuroticism. $\beta$ = Standardized Beta. 95% CI = 95% Confidence Interval. p-value = unadjusted p-value. ^p-FDR^ = False discovery rate correction for multiple testing using Benjamini-Hochberg false discovery rate procedure.

# Table S44 Full model output for best fitting latent growth model including trajectories of emotional difficulties and PGS of depression including parental education as a covariate

Latent Variables:

Estimate Std.Err z-value P(>|z|) ci.lower ci.upper Std.lv Std.all

i1 =~

ytime1 1.000 1.000 1.000 0.883 0.726

ytime2 1.000 1.000 1.000 0.883 0.632

ytime3 1.000 1.000 1.000 0.883 0.675

s1 =~

ytime1 0.000 0.000 0.000 0.000 0.000

ytime2 1.500 1.500 1.500 0.464 0.332

ytime3 3.500 3.500 3.500 1.084 0.828

Regressions:

Estimate Std.Err z-value P(>|z|) ci.lower ci.upper Std.lv Std.all

ytime1 ~

sex 0.025 0.012 2.022 0.043 0.001 0.049 0.025 0.010

by_cat 0.000 0.009 0.024 0.981 -0.017 0.017 0.000 0.000

edu_mf -0.308 0.015 -20.654 0.000 -0.338 -0.279 -0.308 -0.113

ytime2 ~

sex 0.047 0.015 3.219 0.001 0.018 0.076 0.047 0.017

by_cat 0.040 0.010 3.816 0.000 0.019 0.060 0.040 0.021

edu_mf -0.202 0.017 -12.015 0.000 -0.235 -0.169 -0.202 -0.065

ytime3 ~

sex -0.002 0.017 -0.103 0.918 -0.035 0.031 -0.002 -0.001

by_cat 0.075 0.016 4.590 0.000 0.043 0.107 0.075 0.043

edu_mf -0.225 0.021 -10.474 0.000 -0.267 -0.183 -0.225 -0.077

i1 ~

adj_pgs 0.021 0.005 3.933 0.000 0.010 0.031 0.023 0.023

s1 ~

adj_pgs 0.000 0.000 0.000 0.000 0.000

Covariances:

Estimate Std.Err z-value P(>|z|) ci.lower ci.upper Std.lv Std.all

.i1 ~~

.s1 -0.100 0.006 -16.390 0.000 -0.112 -0.088 -0.366 -0.366

Intercepts:

Estimate Std.Err z-value P(>|z|) ci.lower ci.upper Std.lv Std.all

.ytime1 0.000 0.000 0.000 0.000 0.000

.ytime2 0.000 0.000 0.000 0.000 0.000

.ytime3 0.000 0.000 0.000 0.000 0.000

.i1 1.817 0.035 52.000 0.000 1.749 1.886 2.058 2.058

.s1 -0.141 0.018 -7.801 0.000 -0.177 -0.106 -0.456 -0.456

Variances:

Estimate Std.Err z-value P(>|z|) ci.lower ci.upper Std.lv Std.all

.ytime1 0.679 0.017 40.129 0.000 0.646 0.712 0.679 0.459

.ytime2 1.250 0.013 97.201 0.000 1.225 1.275 1.250 0.639

.ytime3 0.446 0.028 16.142 0.000 0.392 0.500 0.446 0.260

.i1 0.780 0.017 45.526 0.000 0.746 0.813 0.999 0.999

.s1 0.096 0.004 26.016 0.000 0.089 0.103 1.000 1.000

**Note:** std.err=standard error, std.lv=Standardized estimates on variances of the latent variables. Std.all= Standardized estimates based on variances of observed and latent variables. Ci.lower= lower 2.5% of 95% confidence interval. Ci.upper= upper 2.5% of 95% confidence interval. The models are run with sex coded (1,2) and by_cat (birth year categorized to three levels, coded as 1,2,3). Adj_pgs= polygenic score. Edu_mf (parental education. coded as 1,2).

# Table S45 Full model output for best fitting latent growth model including trajectories of behavioral difficulties and PGS of depression including parental education as a covariate

Latent Variables:

Estimate Std.Err z-value P(>|z|) ci.lower ci.upper Std.lv Std.all

i1 =~

ytime1 1.000 1.000 1.000 1.716 0.760

ytime2 1.000 1.000 1.000 1.716 0.709

ytime3 1.000 1.000 1.000 1.716 0.753

s1 =~

ytime1 0.000 0.000 0.000 0.000 0.000

ytime2 1.500 1.500 1.500 0.790 0.326

ytime3 3.500 3.500 3.500 1.842 0.808

Regressions:

Estimate Std.Err z-value P(>|z|) ci.lower ci.upper Std.lv Std.all

ytime1 ~

sex -0.365 0.022 -16.386 0.000 -0.408 -0.321 -0.365 -0.081

by_cat -0.095 0.015 -6.284 0.000 -0.125 -0.066 -0.095 -0.031

edu_mf -0.535 0.027 -20.129 0.000 -0.588 -0.483 -0.535 -0.106

ytime2 ~

sex -0.235 0.025 -9.365 0.000 -0.284 -0.186 -0.235 -0.048

by_cat -0.033 0.018 -1.827 0.068 -0.068 0.002 -0.033 -0.010

edu_mf -0.462 0.029 -16.001 0.000 -0.519 -0.406 -0.462 -0.085

ytime3 ~

sex -0.382 0.029 -13.307 0.000 -0.438 -0.326 -0.382 -0.084

by_cat -0.029 0.027 -1.060 0.289 -0.083 0.025 -0.029 -0.009

edu_mf -0.679 0.036 -18.669 0.000 -0.750 -0.608 -0.679 -0.133

i1 ~

adj_pgs 0.026 0.011 2.374 0.018 0.005 0.047 0.015 0.015

s1 ~

adj_pgs 0.021 0.005 4.645 0.000 0.012 0.030 0.040 0.040

Covariances:

Estimate Std.Err z-value P(>|z|) ci.lower ci.upper Std.lv Std.all

.i1 ~~

.s1 -0.337 0.018 -18.243 0.000 -0.373 -0.301 -0.373 -0.373

Intercepts:

Estimate Std.Err z-value P(>|z|) ci.lower ci.upper Std.lv Std.all

.ytime1 0.000 0.000 0.000 0.000 0.000

.ytime2 0.000 0.000 0.000 0.000 0.000

.ytime3 0.000 0.000 0.000 0.000 0.000

.i1 5.612 0.062 91.101 0.000 5.491 5.733 3.270 3.270

.s1 -0.354 0.030 -11.754 0.000 -0.413 -0.295 -0.672 -0.672

Variances:

Estimate Std.Err z-value P(>|z|) ci.lower ci.upper Std.lv Std.all

.ytime1 2.055 0.051 40.499 0.000 1.955 2.154 2.055 0.403

.ytime2 3.248 0.035 91.830 0.000 3.179 3.317 3.248 0.554

.ytime3 1.077 0.077 14.034 0.000 0.927 1.228 1.077 0.207

.i1 2.945 0.053 55.810 0.000 2.842 3.049 1.000 1.000

.s1 0.277 0.011 25.721 0.000 0.256 0.298 0.998 0.998

**Note:** std.err=standard error, std.lv=Standardized estimates on variances of the latent variables. Std.all= Standardized estimates based on variances of observed and latent variables. Ci.lower= lower 2.5% of 95% confidence interval. Ci.upper= upper 2.5% of 95% confidence interval. The models are run with sex coded (1,2) and by_cat (birth year categorized to three levels, coded as 1,2,3). Adj_pgs= polygenic score. Edu_mf (parental education. coded as 1,2).

# Table S46 Full model output for best fitting latent growth model including trajectories of emotional difficulties and PGS of anxiety including parental education as a covariate

Latent Variables:

Estimate Std.Err z-value P(>|z|) ci.lower ci.upper Std.lv Std.all

i1 =~

ytime1 1.000 1.000 1.000 0.883 0.726

ytime2 1.000 1.000 1.000 0.883 0.632

ytime3 1.000 1.000 1.000 0.883 0.675

s1 =~

ytime1 0.000 0.000 0.000 0.000 0.000

ytime2 1.500 1.500 1.500 0.464 0.332

ytime3 3.500 3.500 3.500 1.084 0.828

Regressions:

Estimate Std.Err z-value P(>|z|) ci.lower ci.upper Std.lv Std.all

ytime1 ~

sex 0.025 0.012 2.028 0.043 0.001 0.049 0.025 0.010

by_cat -0.000 0.009 -0.006 0.995 -0.017 0.017 -0.000 -0.000

edu_mf -0.310 0.015 -20.793 0.000 -0.340 -0.281 -0.310 -0.114

ytime2 ~

sex 0.047 0.015 3.216 0.001 0.018 0.076 0.047 0.017

by_cat 0.040 0.010 3.797 0.000 0.019 0.060 0.040 0.021

edu_mf -0.202 0.017 -12.065 0.000 -0.235 -0.170 -0.202 -0.065

ytime3 ~

sex -0.002 0.017 -0.111 0.912 -0.035 0.031 -0.002 -0.001

by_cat 0.075 0.016 4.600 0.000 0.043 0.107 0.075 0.043

edu_mf -0.224 0.021 -10.450 0.000 -0.267 -0.182 -0.224 -0.077

i1 ~

adj_pgs 0.000 0.000 0.000 0.000 0.000

s1 ~

adj_pgs 0.008 0.002 3.459 0.001 0.004 0.013 0.026 0.026

Covariances:

Estimate Std.Err z-value P(>|z|) ci.lower ci.upper Std.lv Std.all

.i1 ~~

.s1 -0.100 0.006 -16.396 0.000 -0.112 -0.088 -0.366 -0.366

Intercepts:

Estimate Std.Err z-value P(>|z|) ci.lower ci.upper Std.lv Std.all

.ytime1 0.000 0.000 0.000 0.000 0.000

.ytime2 0.000 0.000 0.000 0.000 0.000

.ytime3 0.000 0.000 0.000 0.000 0.000

.i1 1.821 0.035 52.112 0.000 1.752 1.889 2.062 2.062

.s1 -0.143 0.018 -7.870 0.000 -0.178 -0.107 -0.461 -0.461

Variances:

Estimate Std.Err z-value P(>|z|) ci.lower ci.upper Std.lv Std.all

.ytime1 0.679 0.017 40.126 0.000 0.646 0.712 0.679 0.459

.ytime2 1.250 0.013 97.200 0.000 1.225 1.275 1.250 0.639

.ytime3 0.446 0.028 16.148 0.000 0.392 0.500 0.446 0.261

.i1 0.780 0.017 45.534 0.000 0.746 0.814 1.000 1.000

.s1 0.096 0.004 26.002 0.000 0.089 0.103 0.999 0.999

**Note:** std.err=standard error, std.lv=Standardized estimates on variances of the latent variables. Std.all= Standardized estimates based on variances of observed and latent variables. Ci.lower= lower 2.5% of 95% confidence interval. Ci.upper= upper 2.5% of 95% confidence interval. The models are run with sex coded (1,2) and by_cat (birth year categorized to three levels, coded as 1,2,3). Adj_pgs= polygenic score. Edu_mf (parental education. coded as 1,2).

# Table S47 Full model output for best fitting latent growth model including trajectories of behavioral difficulties and PGS of anxiety including parental education as a covariate

Latent Variables:

Estimate Std.Err z-value P(>|z|) ci.lower ci.upper Std.lv Std.all

i1 =~

ytime1 1.000 1.000 1.000 1.716 0.760

ytime2 1.000 1.000 1.000 1.716 0.709

ytime3 1.000 1.000 1.000 1.716 0.753

s1 =~

ytime1 0.000 0.000 0.000 0.000 0.000

ytime2 1.500 1.500 1.500 0.790 0.326

ytime3 3.500 3.500 3.500 1.843 0.809

Regressions:

Estimate Std.Err z-value P(>|z|) ci.lower ci.upper Std.lv Std.all

ytime1 ~

sex -0.364 0.022 -16.376 0.000 -0.408 -0.321 -0.364 -0.081

by_cat -0.096 0.015 -6.299 0.000 -0.125 -0.066 -0.096 -0.031

edu_mf -0.538 0.027 -20.232 0.000 -0.590 -0.485 -0.538 -0.106

ytime2 ~

sex -0.235 0.025 -9.364 0.000 -0.284 -0.186 -0.235 -0.048

by_cat -0.033 0.018 -1.865 0.062 -0.068 0.002 -0.033 -0.010

edu_mf -0.467 0.029 -16.154 0.000 -0.523 -0.410 -0.467 -0.086

ytime3 ~

sex -0.381 0.029 -13.265 0.000 -0.437 -0.325 -0.381 -0.084

by_cat -0.031 0.027 -1.134 0.257 -0.085 0.023 -0.031 -0.010

edu_mf -0.686 0.036 -18.868 0.000 -0.757 -0.615 -0.686 -0.135

i1 ~

adj_pgs 0.000 0.000 0.000 0.000 0.000

s1 ~

adj_pgs 0.012 0.004 3.140 0.002 0.005 0.020 0.023 0.023

Covariances:

Estimate Std.Err z-value P(>|z|) ci.lower ci.upper Std.lv Std.all

.i1 ~~

.s1 -0.337 0.018 -18.223 0.000 -0.373 -0.300 -0.373 -0.373

Intercepts:

Estimate Std.Err z-value P(>|z|) ci.lower ci.upper Std.lv Std.all

.ytime1 0.000 0.000 0.000 0.000 0.000

.ytime2 0.000 0.000 0.000 0.000 0.000

.ytime3 0.000 0.000 0.000 0.000 0.000

.i1 5.616 0.062 91.205 0.000 5.495 5.736 3.272 3.272

.s1 -0.351 0.030 -11.650 0.000 -0.409 -0.292 -0.666 -0.666

Variances:

Estimate Std.Err z-value P(>|z|) ci.lower ci.upper Std.lv Std.all

.ytime1 2.054 0.051 40.486 0.000 1.955 2.154 2.054 0.403

.ytime2 3.249 0.035 91.820 0.000 3.180 3.319 3.249 0.554

.ytime3 1.075 0.077 14.000 0.000 0.925 1.226 1.075 0.207

.i1 2.946 0.053 55.821 0.000 2.843 3.050 1.000 1.000

.s1 0.277 0.011 25.750 0.000 0.256 0.298 0.999 0.999

**Note:** std.err=standard error, std.lv=Standardized estimates on variances of the latent variables. Std.all= Standardized estimates based on variances of observed and latent variables. Ci.lower= lower 2.5% of 95% confidence interval. Ci.upper= upper 2.5% of 95% confidence interval. The models are run with sex coded (1,2) and by_cat (birth year categorized to three levels, coded as 1,2,3). Adj_pgs= polygenic score. Edu_mf (parental education. coded as 1,2).

# Table S48 Full model output for best fitting latent growth model including trajectories of emotional difficulties and PGS of neuroticism including parental education as a covariate

Latent Variables:

Estimate Std.Err z-value P(>|z|) ci.lower ci.upper Std.lv Std.all

i1 =~

ytime1 1.000 1.000 1.000 0.883 0.726

ytime2 1.000 1.000 1.000 0.883 0.632

ytime3 1.000 1.000 1.000 0.883 0.675

s1 =~

ytime1 0.000 0.000 0.000 0.000 0.000

ytime2 1.500 1.500 1.500 0.464 0.332

ytime3 3.500 3.500 3.500 1.083 0.828

Regressions:

Estimate Std.Err z-value P(>|z|) ci.lower ci.upper Std.lv Std.all

ytime1 ~

sex 0.025 0.012 2.013 0.044 0.001 0.049 0.025 0.010

by_cat 0.000 0.009 0.040 0.968 -0.017 0.017 0.000 0.000

edu_mf -0.305 0.015 -20.455 0.000 -0.335 -0.276 -0.305 -0.112

ytime2 ~

sex 0.047 0.015 3.209 0.001 0.018 0.076 0.047 0.017

by_cat 0.040 0.010 3.835 0.000 0.020 0.061 0.040 0.021

edu_mf -0.199 0.017 -11.839 0.000 -0.231 -0.166 -0.199 -0.064

ytime3 ~

sex -0.002 0.017 -0.111 0.912 -0.035 0.031 -0.002 -0.001

by_cat 0.076 0.016 4.636 0.000 0.044 0.108 0.076 0.043

edu_mf -0.223 0.021 -10.372 0.000 -0.265 -0.181 -0.223 -0.076

i1 ~

adj_pgs 0.044 0.005 8.374 0.000 0.033 0.054 0.049 0.049

s1 ~

adj_pgs 0.000 0.000 0.000 0.000 0.000

Covariances:

Estimate Std.Err z-value P(>|z|) ci.lower ci.upper Std.lv Std.all

.i1 ~~

.s1 -0.100 0.006 -16.395 0.000 -0.112 -0.088 -0.366 -0.366

Intercepts:

Estimate Std.Err z-value P(>|z|) ci.lower ci.upper Std.lv Std.all

.ytime1 0.000 0.000 0.000 0.000 0.000

.ytime2 0.000 0.000 0.000 0.000 0.000

.ytime3 0.000 0.000 0.000 0.000 0.000

.i1 1.812 0.035 51.865 0.000 1.744 1.881 2.052 2.052

.s1 -0.141 0.018 -7.803 0.000 -0.177 -0.106 -0.457 -0.457

Variances:

Estimate Std.Err z-value P(>|z|) ci.lower ci.upper Std.lv Std.all

.ytime1 0.680 0.017 40.174 0.000 0.646 0.713 0.680 0.459

.ytime2 1.250 0.013 97.222 0.000 1.224 1.275 1.250 0.639

.ytime3 0.447 0.028 16.180 0.000 0.393 0.501 0.447 0.261

.i1 0.778 0.017 45.472 0.000 0.745 0.812 0.998 0.998

.s1 0.096 0.004 25.997 0.000 0.089 0.103 1.000 1.000

**Note:** std.err=standard error, std.lv=Standardized estimates on variances of the latent variables. Std.all= Standardized estimates based on variances of observed and latent variables. Ci.lower= lower 2.5% of 95% confidence interval. Ci.upper= upper 2.5% of 95% confidence interval. The models are run with sex coded (1,2) and by_cat (birth year categorized to three levels, coded as 1,2,3). Adj_pgs= polygenic score. Edu_mf (parental education. coded as 1,2).

# Table S49 Full model output for best fitting latent growth model including trajectories of behavioral difficulties and PGS of neuroticism including parental education as a covariate

Latent Variables:

Estimate Std.Err z-value P(>|z|) ci.lower ci.upper Std.lv Std.all

i1 =~

ytime1 1.000 1.000 1.000 1.717 0.760

ytime2 1.000 1.000 1.000 1.717 0.709

ytime3 1.000 1.000 1.000 1.717 0.753

s1 =~

ytime1 0.000 0.000 0.000 0.000 0.000

ytime2 1.500 1.500 1.500 0.790 0.326

ytime3 3.500 3.500 3.500 1.843 0.809

Regressions:

Estimate Std.Err z-value P(>|z|) ci.lower ci.upper Std.lv Std.all

ytime1 ~

sex -0.364 0.022 -16.381 0.000 -0.408 -0.321 -0.364 -0.081

by_cat -0.095 0.015 -6.264 0.000 -0.125 -0.065 -0.095 -0.031

edu_mf -0.530 0.027 -19.947 0.000 -0.582 -0.478 -0.530 -0.105

ytime2 ~

sex -0.235 0.025 -9.366 0.000 -0.284 -0.186 -0.235 -0.048

by_cat -0.033 0.018 -1.833 0.067 -0.068 0.002 -0.033 -0.010

edu_mf -0.461 0.029 -15.953 0.000 -0.518 -0.404 -0.461 -0.085

ytime3 ~

sex -0.381 0.029 -13.267 0.000 -0.437 -0.325 -0.381 -0.084

by_cat -0.030 0.027 -1.108 0.268 -0.084 0.023 -0.030 -0.010

edu_mf -0.683 0.036 -18.786 0.000 -0.754 -0.612 -0.683 -0.134

i1 ~

adj_pgs 0.068 0.009 7.202 0.000 0.049 0.086 0.039 0.039

s1 ~

adj_pgs 0.000 0.000 0.000 0.000 0.000

Covariances:

Estimate Std.Err z-value P(>|z|) ci.lower ci.upper Std.lv Std.all

.i1 ~~

.s1 -0.337 0.018 -18.253 0.000 -0.373 -0.301 -0.373 -0.373

Intercepts:

Estimate Std.Err z-value P(>|z|) ci.lower ci.upper Std.lv Std.all

.ytime1 0.000 0.000 0.000 0.000 0.000

.ytime2 0.000 0.000 0.000 0.000 0.000

.ytime3 0.000 0.000 0.000 0.000 0.000

.i1 5.603 0.062 90.975 0.000 5.482 5.723 3.263 3.263

.s1 -0.349 0.030 -11.597 0.000 -0.408 -0.290 -0.663 -0.663

Variances:

Estimate Std.Err z-value P(>|z|) ci.lower ci.upper Std.lv Std.all

.ytime1 2.054 0.051 40.499 0.000 1.955 2.153 2.054 0.403

.ytime2 3.249 0.035 91.845 0.000 3.180 3.318 3.249 0.554

.ytime3 1.076 0.077 14.012 0.000 0.925 1.226 1.076 0.207

.i1 2.943 0.053 55.790 0.000 2.840 3.047 0.998 0.998

.s1 0.277 0.011 25.771 0.000 0.256 0.298 1.000 1.000

**Note:** std.err=standard error, std.lv=Standardized estimates on variances of the latent variables. Std.all= Standardized estimates based on variances of observed and latent variables. Ci.lower= lower 2.5% of 95% confidence interval. Ci.upper= upper 2.5% of 95% confidence interval. The models are run with sex coded (1,2) and by_cat (birth year categorized to three levels, coded as 1,2,3). Adj_pgs= polygenic score. Edu_mf (parental education. coded as 1,2).

# Table S50 Full model output for best fitting latent growth model including trajectories of emotional difficulties and PGS of bipolar disorder including parental education as a covariate

Latent Variables:

Estimate Std.Err z-value P(>|z|) ci.lower ci.upper Std.lv Std.all

i1 =~

ytime1 1.000 1.000 1.000 0.883 0.726

ytime2 1.000 1.000 1.000 0.883 0.632

ytime3 1.000 1.000 1.000 0.883 0.675

s1 =~

ytime1 0.000 0.000 0.000 0.000 0.000

ytime2 1.500 1.500 1.500 0.464 0.332

ytime3 3.500 3.500 3.500 1.083 0.828

Regressions:

Estimate Std.Err z-value P(>|z|) ci.lower ci.upper Std.lv Std.all

ytime1 ~

sex 0.025 0.012 2.030 0.042 0.001 0.050 0.025 0.010

by_cat -0.000 0.009 -0.007 0.994 -0.017 0.017 -0.000 -0.000

edu_mf -0.310 0.015 -20.767 0.000 -0.339 -0.281 -0.310 -0.114

ytime2 ~

sex 0.047 0.015 3.221 0.001 0.018 0.076 0.047 0.017

by_cat 0.040 0.010 3.785 0.000 0.019 0.060 0.040 0.021

edu_mf -0.203 0.017 -12.108 0.000 -0.236 -0.170 -0.203 -0.065

ytime3 ~

sex -0.001 0.017 -0.086 0.932 -0.035 0.032 -0.001 -0.001

by_cat 0.074 0.016 4.556 0.000 0.042 0.107 0.074 0.042

edu_mf -0.226 0.021 -10.537 0.000 -0.268 -0.184 -0.226 -0.077

i1 ~

adj_pgs 0.000 0.000 0.000 0.000 0.000

s1 ~

adj_pgs 0.000 0.000 0.000 0.000 0.000

Covariances:

Estimate Std.Err z-value P(>|z|) ci.lower ci.upper Std.lv Std.all

.i1 ~~

.s1 -0.100 0.006 -16.369 0.000 -0.112 -0.088 -0.365 -0.365

Intercepts:

Estimate Std.Err z-value P(>|z|) ci.lower ci.upper Std.lv Std.all

.ytime1 0.000 0.000 0.000 0.000 0.000

.ytime2 0.000 0.000 0.000 0.000 0.000

.ytime3 0.000 0.000 0.000 0.000 0.000

.i1 1.820 0.035 52.090 0.000 1.752 1.889 2.061 2.061

.s1 -0.141 0.018 -7.801 0.000 -0.177 -0.106 -0.457 -0.457

Variances:

Estimate Std.Err z-value P(>|z|) ci.lower ci.upper Std.lv Std.all

.ytime1 0.679 0.017 40.135 0.000 0.646 0.713 0.679 0.459

.ytime2 1.250 0.013 97.189 0.000 1.224 1.275 1.250 0.639

.ytime3 0.446 0.028 16.142 0.000 0.392 0.500 0.446 0.260

.i1 0.780 0.017 45.530 0.000 0.746 0.813 1.000 1.000

.s1 0.096 0.004 26.005 0.000 0.089 0.103 1.000 1.000

**Note:** std.err=standard error, std.lv=Standardized estimates on variances of the latent variables. Std.all= Standardized estimates based on variances of observed and latent variables. Ci.lower= lower 2.5% of 95% confidence interval. Ci.upper= upper 2.5% of 95% confidence interval. The models are run with sex coded (1,2) and by_cat (birth year categorized to three levels, coded as 1,2,3). Adj_pgs= polygenic score. Edu_mf (parental education. coded as 1,2).

# Table S51 Full model output for best fitting latent growth model including trajectories of behavioral difficulties and PGS of bipolar disorder including parental education as a covariate

Latent Variables:

Estimate Std.Err z-value P(>|z|) ci.lower ci.upper Std.lv Std.all

i1 =~

ytime1 1.000 1.000 1.000 1.717 0.760

ytime2 1.000 1.000 1.000 1.717 0.709

ytime3 1.000 1.000 1.000 1.717 0.753

s1 =~

ytime1 0.000 0.000 0.000 0.000 0.000

ytime2 1.500 1.500 1.500 0.790 0.326

ytime3 3.500 3.500 3.500 1.843 0.808

Regressions:

Estimate Std.Err z-value P(>|z|) ci.lower ci.upper Std.lv Std.all

ytime1 ~

sex -0.364 0.022 -16.374 0.000 -0.408 -0.321 -0.364 -0.081

by_cat -0.096 0.015 -6.298 0.000 -0.125 -0.066 -0.096 -0.031

edu_mf -0.537 0.027 -20.220 0.000 -0.589 -0.485 -0.537 -0.106

ytime2 ~

sex -0.235 0.025 -9.360 0.000 -0.284 -0.186 -0.235 -0.048

by_cat -0.034 0.018 -1.877 0.060 -0.068 0.001 -0.034 -0.010

edu_mf -0.468 0.029 -16.199 0.000 -0.525 -0.411 -0.468 -0.086

ytime3 ~

sex -0.380 0.029 -13.240 0.000 -0.437 -0.324 -0.380 -0.083

by_cat -0.032 0.027 -1.170 0.242 -0.086 0.022 -0.032 -0.010

edu_mf -0.689 0.036 -18.960 0.000 -0.761 -0.618 -0.689 -0.135

i1 ~

adj_pgs 0.000 0.000 0.000 0.000 0.000

s1 ~

adj_pgs 0.000 0.000 0.000 0.000 0.000

Covariances:

Estimate Std.Err z-value P(>|z|) ci.lower ci.upper Std.lv Std.all

.i1 ~~

.s1 -0.337 0.018 -18.215 0.000 -0.373 -0.300 -0.372 -0.372

Intercepts:

Estimate Std.Err z-value P(>|z|) ci.lower ci.upper Std.lv Std.all

.ytime1 0.000 0.000 0.000 0.000 0.000

.ytime2 0.000 0.000 0.000 0.000 0.000

.ytime3 0.000 0.000 0.000 0.000 0.000

.i1 5.615 0.062 91.190 0.000 5.495 5.736 3.271 3.271

.s1 -0.349 0.030 -11.587 0.000 -0.408 -0.290 -0.662 -0.662

Variances:

Estimate Std.Err z-value P(>|z|) ci.lower ci.upper Std.lv Std.all

.ytime1 2.054 0.051 40.481 0.000 1.954 2.153 2.054 0.403

.ytime2 3.249 0.035 91.812 0.000 3.180 3.318 3.249 0.554

.ytime3 1.076 0.077 14.006 0.000 0.925 1.226 1.076 0.207

.i1 2.947 0.053 55.826 0.000 2.843 3.050 1.000 1.000

.s1 0.277 0.011 25.752 0.000 0.256 0.298 1.000 1.000

**Note:** std.err=standard error, std.lv=Standardized estimates on variances of the latent variables. Std.all= Standardized estimates based on variances of observed and latent variables. Ci.lower= lower 2.5% of 95% confidence interval. Ci.upper= upper 2.5% of 95% confidence interval. The models are run with sex coded (1,2) and by_cat (birth year categorized to three levels, coded as 1,2,3). Adj_pgs= polygenic score. Edu_mf (parental education. coded as 1,2).

Table S52: Relative odds of any emotional disorder given assignment to specific developmental profile including parental education as a covariate.

| **Comparison** | **Odds ratio (OR)** | **95% CI** | |
| --- | --- | --- | --- |
|  |  | **Lower 2.5%** | **Upper 2.5%** |
| Profile 1 vs. Reference | 2.669 | 2.213 | 3.124 |
| Profile 3 vs. Reference | 1.577 | 1.274 | 1.880 |
| Profile 4 vs. Reference | 1.744 | 1.317 | 2.170 |
| Profile 5 vs. Reference | 2.922 | 2.083 | 3.761 |
| Profile 3 vs. Profile 1 | 0.591 | 0.452 | 0.729 |
| Profile 4 vs. Profile 1 | 0.653 | 0.466 | 0.841 |
| Profile 5 vs. Profile 1 | 1.095 | 0.743 | 1.447 |
| Profile 4 vs. Profile 3 | 1.106 | 0.774 | 1.437 |
| Profile 5 vs. Profile 3 | 1.853 | 1.249 | 2.458 |
| Profile 5 vs. Profile 4 | 1.676 | 1.020 | 2.333 |

**Note:** All profiles were compared to each other.

# Table S53: Relative odds of assignment to specific developmental profile per standard deviation increase in polygenic score for depression including parental education as a covariate.

| **Comparison** | **Odds ratio (OR)** | **95% CI** | |
| --- | --- | --- | --- |
|  |  | **Lower 2.5%** | **Upper 2.5%** |
| Profile 1 vs. Reference | 1.093 | 1.027 | 1.165 |
| Profile 3 vs. Reference | 1.108 | 1.047 | 1.171 |
| Profile 4 vs. Reference | 1.121 | 1.032 | 1.216 |
| Profile 5 vs. Reference | 1.182 | 1.063 | 1.313 |
| Profile 3 vs. Profile 1 | 1.013 | 0.936 | 1.096 |
| Profile 4 vs. Profile 1 | 1.025 | 0.925 | 1.135 |
| Profile 5 vs. Profile 1 | 1.081 | 0.958 | 1.219 |
| Profile 4 vs. Profile 3 | 1.012 | 0.917 | 1.116 |
| Profile 5 vs. Profile 3 | 1.067 | 0.950 | 1.198 |
| Profile 5 vs. Profile 4 | 1.054 | 0.914 | 1.216 |

**Note**: All profiles were compared to each other.

# Table S54: Relative odds of assignment to specific developmental profile per standard deviation increase in polygenic score for anxiety including parental education as a covariate.

| **Comparison** | **Odds ratio (OR)** | **95% CI** | |
| --- | --- | --- | --- |
|  |  | **Lower 2.5%** | **Upper 2.5%** |
| Profile 1 vs. Reference | 1.083 | 1.019 | 1.150 |
| Profile 3 vs. Reference | 1.070 | 1.012 | 1.131 |
| Profile 4 vs. Reference | 1.026 | 0.948 | 1.110 |
| Profile 5 vs. Reference | 1.072 | 0.967 | 1.189 |
| Profile 3 vs. Profile 1 | 0.988 | 0.915 | 1.067 |
| Profile 4 vs. Profile 1 | 0.947 | 0.858 | 1.046 |
| Profile 5 vs. Profile 1 | 0.991 | 0.881 | 1.114 |
| Profile 4 vs. Profile 3 | 0.959 | 0.872 | 1.054 |
| Profile 5 vs. Profile 3 | 1.002 | 0.894 | 1.123 |
| Profile 5 vs. Profile 4 | 1.046 | 0.912 | 1.199 |

**Note:** All profiles were compared to each other.

# Table S55: Relative odds of assignment to specific developmental profile per standard deviation increase in polygenic score for bipolar disorder including parental education as a covariate.

| **Comparison** | **Odds ratio (OR)** | **95% CI** | |
| --- | --- | --- | --- |
|  |  | **Lower 2.5%** | **Upper 2.5%** |
| Profile 1 vs. Reference | 1.008 | 0.948 | 1.071 |
| Profile 3 vs. Reference | 1.033 | 0.978 | 1.091 |
| Profile 4 vs. Reference | 1.022 | 0.944 | 1.107 |
| Profile 5 vs. Reference | 1.209 | 1.087 | 1.345 |
| Profile 3 vs. Profile 1 | 1.025 | 0.949 | 1.107 |
| Profile 4 vs. Profile 1 | 1.014 | 0.919 | 1.120 |
| Profile 5 vs. Profile 1 | 1.200 | 1.063 | 1.354 |
| Profile 4 vs. Profile 3 | 0.990 | 0.900 | 1.089 |
| Profile 5 vs. Profile 3 | 1.171 | 1.042 | 1.316 |
| Profile 5 vs. Profile 4 | 1.183 | 1.027 | 1.362 |

**Note**: All profiles were compared to each other.

# Table S56: Relative odds of assignment to specific developmental profile per standard deviation increase in polygenic score for neuroticism including parental education as a covariate.

| **Comparison** | **Odds ratio (OR)** | **95% CI** | |
| --- | --- | --- | --- |
|  |  | **Lower 2.5%** | **Upper 2.5%** |
| Profile 1 vs. Reference | 1.184 | 1.113 | 1.261 |
| Profile 3 vs. Reference | 1.055 | 0.998 | 1.114 |
| Profile 4 vs. Reference | 1.149 | 1.063 | 1.242 |
| Profile 5 vs. Reference | 1.032 | 0.927 | 1.149 |
| Profile 3 vs. Profile 1 | 0.891 | 0.824 | 0.963 |
| Profile 4 vs. Profile 1 | 0.970 | 0.879 | 1.071 |
| Profile 5 vs. Profile 1 | 0.872 | 0.772 | 0.985 |
| Profile 4 vs. Profile 3 | 1.089 | 0.992 | 1.196 |
| Profile 5 vs. Profile 3 | 0.979 | 0.870 | 1.101 |
| Profile 5 vs. Profile 4 | 0.898 | 0.781 | 1.034 |

**Note:** All profiles were compared to each other.

# SUPPLEMENTARY REFERENCES

Achenbach, T. M., & Ruffle, T. M. (2000). The Child Behavior Checklist and related forms for assessing behavioral/emotional difficulties and competencies. Pediatrics in Review, 21(8), 265-271.

Akdeniz BC, Frei O, Hagen E, Filiz TT, [Karthikeyan](https://arxiv.org/search/q-bio?searchtype=author&query=Karthikeyan%2C+S) S, et al. (2022). COGEDAP: A Comprehensive Genomic Data Analysis Platform. [arXiv:2212.14103](https://arxiv.org/abs/2212.14103) [q-bio.GN]

Angold, A., Costello, E. J., Messer, S. C., & Pickles, A. (1995). Development of a short questionnaire for use in epidemiological studies of depression in children and adolescents. International Journal of Methods in Psychiatric Research, 5, 237-249.

Bakken, N. R., Hannigan, L. J., Shadrin, A., Hindley, G., Ask, H., Reichborn‐Kjennerud, T., Tesli, M., Andreassen, O. A. & Havdahl, A. (2023). Childhood temperamental, emotional, and behavioral characteristics associated with mood and anxiety disorders in adolescence: A prospective study. Acta Psychiatrica Scandinavica. 147, 217-228.

Birmaher, B., Brent, D. A., Chiappetta, L., Bridge, J., Monga, S., & Baugher, M. (1999). Psychometric Properties of the Screen for Child Anxiety Related Emotional Disorders (SCARED): A Replication Study. Journal of the American Academy of Child & Adolescent Psychiatry, 38, 1230-1236.

Frei O, Jangmo A, Hagen E, Akdeniz BC, Zetterberg R, Filiz TT, Shorter J. (2023). comorment/containers: CoMorMent-Containers-v1.1 (v1.2). Zenodo. <https://doi.org/10.5281/zenodo.7924337>

Hannigan, L. J., Askeland, R. B., Ask, H., Tesli, M., Corfield, E., Ayorech, Z., Helgeland, Ø., Magnus, P., Njølstad, P. R., Øyen, A. S., Stoltenberg, C., Andreassen, O. A., Davey Smith, G., Reichborn-Kjennerud, T. & Havdahl, A. (2021). Genetic Liability for Schizophrenia and Childhood Psychopathology in the General Population. Schizophrenia Bulletin, 47, 1179-1189.

Howard, D. M., Adams, M. J., Shirali, M., Clarke, T.-K., Marioni, R. E., Davies, G., Coleman, J. R. I., Alloza, C., Shen, X., Barbu, M. C., Wigmore, E. M., Gibson, J., Hagenaars, S. P., Lewis, C. M., Ward, J., Smith, D. J., Sullivan, P. F., Haley, C. S., Breen, G., Deary, I. J. & Mcintosh, A. M. (2018). Genome-wide association study of depression phenotypes in UK Biobank identifies variants in excitatory synaptic pathways. Nature Communications, 9(1), 1470.

Howard, D. M., Adams, M. J., Clarke, T.-K., Hafferty, J. D., Gibson, J., Shirali, M., Coleman, J. R. I., Hagenaars, S. P., Ward, J., Wigmore, E. M., Alloza, C., Shen, X., Barbu, M. C., Xu, E. Y., Whalley, H. C., Marioni, R. E., Porteous, D. J., Davies, G., Deary, I. J., Hemani, G., Berger, K., Teismann, H., Rawal, R., Arolt, V., Baune, B. T., Dannlowski, U., Domschke, K., Tian, C., Hinds, D. A., Trzaskowski, M., Byrne, E. M., Ripke, S., Smith, D. J., Sullivan, P. F., Wray, N. R., Breen, G., Lewis, C. M. & Mcintosh, A. M. (2019). Genome-wide meta-analysis of depression identifies 102 independent variants and highlights the importance of the prefrontal brain regions. Nature Neuroscience, 22, 343-352.

Hyde, C. L., Nagle, M. W., Tian, C., Chen, X., Paciga, S. A., Wendland, J. R., Tung, J. Y., Hinds, D. A., Perlis, R. H. & Winslow, A. R. (2016). Identification of 15 genetic loci associated with risk of major depression in individuals of European descent. Nature Genetics, 48, 1031-1036.

Massey, F. J. (1951). The Kolmogorov-Smirnov Test for Goodness of Fit. Journal of the American Statistical Association, 46(253), 68-78.

Mullins, N., Forstner, A. J., O’connell, K. S., Coombes, B., Coleman, J. R. I., Qiao, Z., Als, T. D., Bigdeli, T. B., Børte, S., Bryois, J., Charney, A. W., Drange, O. K., Gandal, M. J., Hagenaars, S. P., Ikeda, M., Kamitaki, N., Kim, M., Krebs, K., Panagiotaropoulou, G., Schilder, B. M., Sloofman, L. G., Steinberg, S., Trubetskoy, V., Winsvold, B. S., Won, H.-H., Abramova, L., Adorjan, K., Agerbo, E., Al Eissa, M., Albani, D., Alliey-Rodriguez, N., Anjorin, A., Antilla, V., Antoniou, A., Awasthi, S., Baek, J. H., Bækvad-Hansen, M., Bass, N., Bauer, M., Beins, E. C., Bergen, S. E., Birner, A., Bøcker Pedersen, C., Bøen, E., Boks, M. P., Bosch, R., Brum, M., Brumpton, B. M., Brunkhorst-Kanaan, N., Budde, M., Bybjerg-Grauholm, J., Byerley, W., Cairns, M., Casas, M., Cervantes, P., Clarke, T.-K., Cruceanu, C., Cuellar-Barboza, A., Cunningham, J., Curtis, D., Czerski, P. M., Dale, A. M., Dalkner, N., David, F. S., Degenhardt, F., Djurovic, S., Dobbyn, A. L., Douzenis, A., Elvsåshagen, T., Escott-Price, V., Ferrier, I. N., Fiorentino, A., Foroud, T. M., Forty, L., Frank, J., Frei, O., Freimer, N. B., Frisén, L., Gade, K., Garnham, J., Gelernter, J., Giørtz Pedersen, M., Gizer, I. R., Gordon, S. D., Gordon-Smith, K., Greenwood, T. A., Grove, J., Guzman-Parra, J., Ha, K., Haraldsson, M., Hautzinger, M., Heilbronner, U., Hellgren, D., Herms, S., Hoffmann, P., Holmans, P. A., Huckins, L., Jamain, S., Johnson, J. S., Kalman, J. L., Kamatani, Y., Kennedy, J. L., Kittel-Schneider, S., Knowles, J. A., Kogevinas, M., Koromina, M., Kranz, T. M., Kranzler, H. R., Kubo, M., Kupka, R., Kushner, S. A., Lavebratt, C., Lawrence, J., Leber, M., Lee, H.-J., Lee, P. H., Levy, S. E., Lewis, C., Liao, C., Lucae, S., Lundberg, M., Macintyre, D. J., Magnusson, S. H., Maier, W., Maihofer, A., Malaspina, D., Maratou, E., Martinsson, L., Mattheisen, M., Mccarroll, S. A., Mcgregor, N. W., Mcguffin, P., Mckay, J. D., Medeiros, H., Medland, S. E., Millischer, V., Montgomery, G. W., Moran, J. L., Morris, D. W., Mühleisen, T. W., O’brien, N., O’donovan, C., Olde Loohuis, L. M., Oruc, L., Papiol, S., Pardiñas, A. F., Perry, A., Pfennig, A., Porichi, E., Potash, J. B., Quested, D., Raj, T., Rapaport, M. H., Depaulo, J. R., Regeer, E. J., Rice, J. P., Rivas, F., Rivera, M., Roth, J., Roussos, P., Ruderfer, D. M., Sánchez-Mora, C., Schulte, E. C., Senner, F., Sharp, S., Shilling, P. D., Sigurdsson, E., Sirignano, L., Slaney, C., Smeland, O. B., Smith, D. J., Sobell, J. L., Søholm Hansen, C., Soler Artigas, M., Spijker, A. T., Stein, D. J., Strauss, J. S., Świątkowska, B., Terao, C., Thorgeirsson, T. E., Toma, C., Tooney, P., Tsermpini, E.-E., Vawter, M. P., Vedder, H., Walters, J. T. R., Witt, S. H., Xi, S., Xu, W., Yang, J. M. K., Young, A. H., Young, H., Zandi, P. P., Zhou, H., Zillich, L., Adolfsson, R., Agartz, I., Alda, M., Alfredsson, L., Babadjanova, G., Backlund, L., Baune, B. T., Bellivier, F., Bengesser, S., Berrettini, W. H., Blackwood, D. H. R., Boehnke, M., Børglum, A. D., Breen, G., Carr, V. J., Catts, S., Corvin, A., Craddock, N., Dannlowski, U., Dikeos, D., Esko, T., Etain, B., Ferentinos, P., Frye, M., Fullerton, J. M., Gawlik, M., Gershon, E. S., Goes, F. S., Green, M. J., Grigoroiu-Serbanescu, M., Hauser, J., Henskens, F., Hillert, J., Hong, K. S., Hougaard, D. M., Hultman, C. M., Hveem, K., Iwata, N., Jablensky, A. V., Jones, I., Jones, L. A., Kahn, R. S., Kelsoe, J. R., Kirov, G., Landén, M., Leboyer, M., Lewis, C. M., Li, Q. S., Lissowska, J., Lochner, C., Loughland, C., Martin, N. G., Mathews, C. A., Mayoral, F., Mcelroy, S. L., Mcintosh, A. M., Mcmahon, F. J., Melle, I., Michie, P., Milani, L., Mitchell, P. B., Morken, G., Mors, O., Mortensen, P. B., Mowry, B., Müller-Myhsok, B., Myers, R. M., Neale, B. M., Nievergelt, C. M., Nordentoft, M., Nöthen, M. M., O’donovan, M. C., Oedegaard, K. J., Olsson, T., Owen, M. J., Paciga, S. A., Pantelis, C., Pato, C., Pato, M. T., Patrinos, G. P., Perlis, R. H., Posthuma, D., Ramos-Quiroga, J. A., Reif, A., Reininghaus, E. Z., Ribasés, M., Rietschel, M., Ripke, S., Rouleau, G. A., Saito, T., Schall, U., Schalling, M., Schofield, P. R., Schulze, T. G., Scott, L. J., Scott, R. J., Serretti, A., Shannon Weickert, C., Smoller, J. W., Stefansson, H., Stefansson, K., Stordal, E., Streit, F., Sullivan, P. F., Turecki, G., Vaaler, A. E., Vieta, E., Vincent, J. B., Waldman, I. D., Weickert, T. W., Werge, T., Wray, N. R., Zwart, J.-A., Biernacka, J. M., Nurnberger, J. I., Cichon, S., Edenberg, H. J., Stahl, E. A., Mcquillin, A., Di Florio, A., Ophoff, R. A. & Andreassen, O. A. (2021). Genome-wide association study of more than 40,000 bipolar disorder cases provides new insights into the underlying biology. Nature Genetics, 53, 817-829.

Nagel, M., Jansen, P. R., Stringer, S., Watanabe, K., De Leeuw, C. A., Bryois, J., Savage, J. E., Hammerschlag, A. R., Skene, N. G., MuñOz-Manchado, A. B., White, T., Tiemeier, H., Linnarsson, S., Hjerling-Leffler, J., Polderman, T. J. C., Sullivan, P. F., Van Der Sluis, S. & Posthuma, D. (2018). Meta-analysis of genome-wide association studies for neuroticism in 449,484 individuals identifies novel genetic loci and pathways. Nature Genetics, 50, 920-927.

Nagelkerke, N. J. D. (1991). A note on a general definition of the coefficient of determination. Biometrika, 78(3), 691-692.

Privé, F., Aschard, H., Ziyatdinov, A., & Blum, M. G. B. (2018). Efficient analysis of large-scale genome-wide data with two R packages: bigstatsr and bigsnpr. Bioinformatics, 34, 2781-2787.

Privé, F., Arbel, J., & Vilhjálmsson, B. J. (2021). LDpred2: better, faster, stronger. Bioinformatics, 36, 5424-5431.

Privé F (2022). LD reference for HapMap3+. figshare. Dataset. <https://doi.org/10.6084/m9.figshare.21305061.v2>

Purves, K. L., Coleman, J. R. I., Meier, S. M., Rayner, C., Davis, K. a. S., Cheesman, R., Bækvad-Hansen, M., Børglum, A. D., Wan Cho, S., Jürgen Deckert, J., Gaspar, H. A., Bybjerg-Grauholm, J., Hettema, J. M., Hotopf, M., Hougaard, D., Hübel, C., Kan, C., Mcintosh, A. M., Mors, O., Bo Mortensen, P., Nordentoft, M., Werge, T., Nicodemus, K. K., Mattheisen, M., Breen, G. & Eley, T. C. (2020). A major role for common genetic variation in anxiety disorders. Molecular Psychiatry, 25, 3292-3303.

Silva, R. R., Alpert, M., Pouget, E., Silva, V., Trosper, S., Reyes, K., & Dummit, S. (2005). A Rating Scale for Disruptive Behavior Disorders, Based on the DSM-IV Item Pool. Psychiatric Quarterly, 76, 327-339.

Wray, N. R., Ripke, S., Mattheisen, M., Trzaskowski, M., Byrne, E. M., Abdellaoui, A., Adams, M. J., Agerbo, E., Air, T. M., Andlauer, T. M. F., Bacanu, S.-A., Bækvad-Hansen, M., Beekman, A. F. T., Bigdeli, T. B., Binder, E. B., Blackwood, D. R. H., Bryois, J., Buttenschøn, H. N., Bybjerg-Grauholm, J., Cai, N., Castelao, E., Christensen, J. H., Clarke, T.-K., Coleman, J. I. R., Colodro-Conde, L., Couvy-Duchesne, B., Craddock, N., Crawford, G. E., Crowley, C. A., Dashti, H. S., Davies, G., Deary, I. J., Degenhardt, F., Derks, E. M., Direk, N., Dolan, C. V., Dunn, E. C., Eley, T. C., Eriksson, N., Escott-Price, V., Kiadeh, F. H. F., Finucane, H. K., Forstner, A. J., Frank, J., Gaspar, H. A., Gill, M., Giusti-Rodríguez, P., Goes, F. S., Gordon, S. D., Grove, J., Hall, L. S., Hannon, E., Hansen, C. S., Hansen, T. F., Herms, S., Hickie, I. B., Hoffmann, P., Homuth, G., Horn, C., Hottenga, J.-J., Hougaard, D. M., Hu, M., Hyde, C. L., Ising, M., Jansen, R., Jin, F., Jorgenson, E., Knowles, J. A., Kohane, I. S., Kraft, J., Kretzschmar, W. W., Krogh, J., Kutalik, Z., Lane, J. M., Li, Y., Li, Y., Lind, P. A., Liu, X., Lu, L., Macintyre, D. J., Mackinnon, D. F., Maier, R. M., Maier, W., Marchini, J., Mbarek, H., Mcgrath, P., Mcguffin, P., Medland, S. E., Mehta, D., Middeldorp, C. M., Mihailov, E., Milaneschi, Y., Milani, L., Mill, J., Mondimore, F. M., Montgomery, G. W., Mostafavi, S., Mullins, N., Nauck, M., Ng, B., Nivard, M. G., Nyholt, D. R., O’reilly, P. F., Oskarsson, H., Owen, M. J., Painter, J. N., Pedersen, C. B., Pedersen, M. G., Peterson, R. E., Pettersson, E., Peyrot, W. J., Pistis, G., Posthuma, D., Purcell, S. M., Quiroz, J. A., Qvist, P., Rice, J. P., Riley, B. P., Rivera, M., Saeed Mirza, S., Saxena, R., Schoevers, R., Schulte, E. C., Shen, L., Shi, J., Shyn, S. I., Sigurdsson, E., Sinnamon, G. B. C., Smit, J. H., Smith, D. J., Stefansson, H., Steinberg, S., Stockmeier, C. A., Streit, F., Strohmaier, J., Tansey, K. E., Teismann, H., Teumer, A., Thompson, W., Thomson, P. A., Thorgeirsson, T. E., Tian, C., Traylor, M., Treutlein, J., Trubetskoy, V., Uitterlinden, A. G., Umbricht, D., Van Der Auwera, S., Van Hemert, A. M., Viktorin, A., Visscher, P. M., Wang, Y., Webb, B. T., Weinsheimer, S. M., Wellmann, J., Willemsen, G., Witt, S. H., Wu, Y., Xi, H. S., Yang, J., Zhang, F., Arolt, V., Baune, B. T., Berger, K., Boomsma, D. I., Cichon, S., Dannlowski, U., De Geus, E. C. J., Depaulo, J. R., Domenici, E., Domschke, K., Esko, T., Grabe, H. J., Hamilton, S. P., Hayward, C., Heath, A. C., Hinds, D. A., Kendler, K. S., Kloiber, S., Lewis, G., Li, Q. S., Lucae, S., Madden, P. F. A., Magnusson, P. K., Martin, N. G., Mcintosh, A. M., Metspalu, A., Mors, O., Mortensen, P. B., Müller-Myhsok, B., Nordentoft, M., Nöthen, M. M., O’donovan, M. C., Paciga, S. A., Pedersen, N. L., Penninx, B. W. J. H., Perlis, R. H., Porteous, D. J., Potash, J. B., Preisig, M., Rietschel, M., Schaefer, C., Schulze, T. G., Smoller, J. W., Stefansson, K., Tiemeier, H., Uher, R., Völzke, H., Weissman, M. M., Werge, T., Winslow, A. R., Lewis, C. M., Levinson, D. F., Breen, G., Børglum, A. D. & Sullivan, P. F. (2018). Genome-wide association analyses identify 44 risk variants and refine the genetic architecture of major depression. Nature Genetics, 50, 668-681.
